# Synthesis, antiplasmodial activity and in silico molecular docking study of pinocembrin and its analogs

**DOI:** 10.1186/s13065-022-00831-z

**Published:** 2022-05-24

**Authors:** Yadessa Melaku, Melat Solomon, Rajalakshmanan Eswaramoorthy, Uwe Beifuss, Vladimir Ondrus, Yalemtsehay Mekonnen

**Affiliations:** 1 Chemistry Department, Adama Science and Technology University, 1888 Adama, Ethiopia; 2 Biology Department, Addis Ababa University, Addis Ababa, Ethiopia; 3 Bioorganische Chemie, Institut Für Chemie, Universität Hohenheim, Garbenstraße 30, 70599 Stuttgart, Germany; 4 Department of Chemical Engineering, FH Münster-University of Applied Sciences, Stegerwaldstrasse 39, 48565 Steinfurt, Germany; 5 Department of Biomaterials, Saveetha Dental College and Hospitals, Saveetha Institute of Meidcal and Technical Sciences (SIMATS), Saveetha University, Chennai, 600 077 India

**Keywords:** Catalytic hydrogenation, Claisen-Schmidt Condensation, Malaria, *Plasmodium berghei*, Pinocembrin, Chloropinocembrin

## Abstract

**Background:**

Malaria remains the major health problem responsible for many mortality and morbidity in developing countries. Because of the development of resistance by *Plasmodium* species, searching effective antimalarial agents becomes increasingly important. Pinocembrin is a flavanone previously isolated as the most active antiplasmodial compound from the leaves of *Dodonaea angustifolia*. For a better understanding of the antiplasmodial activity, the synthesis of pinocembrin and a great number of analogs was undertaken.

**Methods:**

Chalcones **5a-r** were synthesized via Claisen-Schmidt condensation using 2,4-dibenzyloxy-6-hydroxyacetophenone and aromatic aldehydes as substrates under basic conditions. Cyclization of compounds **5a-r** to the corresponding dibenzylated pinocembrin analogs **6a-r** was achieved using NaOAc in EtOH under reflux. Catalytic hydrogenation using 10% Pd/C as catalyst in an H-Cube Pro was used for debenzylation to deliver **7a**-**l**. The structures of the synthesized compounds were characterized using various physical and spectroscopic methods, including mp, UV, IR, NMR, MS and HRMS. The synthesized dibenzylated flavanones **6a-d, 6i** and **7a** were evaluated for their in vivo antiplasmodial activities against *Plasmodium berghei* infected mice. Molecular docking simulation and drug likeness properties of compounds **7a-l** were assessed using AutoDock Vina and SwissADME, respectively.

**Results:**

A series of chalcones **5a-r** has been synthesized in yields ranging from 46 to 98%. Treatment of the chalcones **5a-r** with NaOAc refluxing in EtOH afforded the dibenzylated pinocembrin analogs **6a-r** with yields up to 54%. Deprotection of the dibenzylated pinocembrin analogs delivered the products **7a-l** in yields ranging from 78 to 94%. The dibenzylated analogs of pinocembrin displayed percent inhibition of parastaemia in the range between 17.4 and 87.2% at 30 mg/kg body weight. The parastaemia inhibition of 87.2 and 55.6% was obtained on treatment of the infected mice with pinocembrin (**7a**) and 4’-chloro-5,7-dibenzylpinocembrin (**6e**), respectively. The mean survival times of those infected mice treated with these two compounds were beyond 14 days indicating that the samples suppressed *P. berghei* and reduced the overall pathogenic effect of the parasite. The molecular docking analysis of the chloro derivatives of pinocembrin revealed that compounds **7a-l** show docking affinities ranging from – 8.1 to – 8.4 kcal/mol while it was -7.2 kcal/mol for chloroquine.

**Conclusion:**

Pinocembrin (**7a**) and 4’-chloro-5,7-dibenzyloxyflavanone (**6e**) displayed good antiplasmodial activity. The in silico docking simulation against *P. falciparum* dihydrofolate reductase-thymidylate synthase revealed that pinocembrin (**7a**) and its chloro analogs **7a-l** showed better binding affinity compared with chloroquine that was used as a standard drug. This is in agreement with the drug-like properties of compounds **7a-l** which fulfill Lipinski's rule of five with zero violations. Therefore, pinocembrin and its chloro analogs could serve as lead compounds for further antiplasmodial drug development.

**Supplementary Information:**

The online version contains supplementary material available at 10.1186/s13065-022-00831-z.

## Introduction

Malaria remains one of the major health problems in developing countries with high recorded rates of mortality and morbidity [1]. The most predominant species responsible for 90% of malarial cases worldwide is *Plasmodium falciparum* [2]. Nearly half of the world’s population resides in regions where malaria is endemic and are thus at risk of infection [3]. The burden is severe in sub-Saharan Africa which account for 90% of the deaths where 5% of children die from the disease before reaching 5 years of age [4]. Efforts to reduce the spread have been exacerbated by the increasing resistance of the mosquito to insecticides, and of the parasite to the currently available drugs [4]. This necessitates search for alternative drugs at reasonable cost for use against malaria.

Flavonoids are phenolic compounds possessing enormous pharmacological activities. They play significant roles in promoting health and preventing chronic degenerative diseases. Among flavonoids, pinocembrin (**7a**) is a flavanone with wide arrays of biological activities including antimicrobial, antiinflammatory, antioxidant, anticancer [5] and against ischemic stroke [6]. Pinocembrin (**7a**) has also been reported having antifibrotic effects in addition to its ability to decrease proinflammatory cytokines production [7] and protective capacity against gastric tissue damage [8]. In our previous antiplasmodial study, we isolated pinocembrin (**7a**) as the most active compound from the leaves of *D. angustifolia* [9]. To establish its antiplasmodial activity large quantities of this compound are required which however is present only as a minor constituent in the leaves of *D. angustifolia*. The synthesis of pinocembrin analogs with improved antimalarial activity are therefore encouraged for further advances. Hence, to further optimize the antiplasmodial activities of pinocembrin (**7a**), the synthesis of this natural product and analogs thereof were undertaken. In this paper, we report the synthesis and antiplasmodial activities of pinocembrin (**7a**) and its analogs against *Plasmodium berghei* in infected mice. Furthermore, the in silico molecular docking analysis against *P. falciparum* dihydrofolate reductase-thymidylate synthase and the drug-like properties of compounds **7a-l** are also presented herein.

## Results and discussion

### Synthesis of Pinocembrin (7a) and its analogs 7b-l

**Fig. 1:**
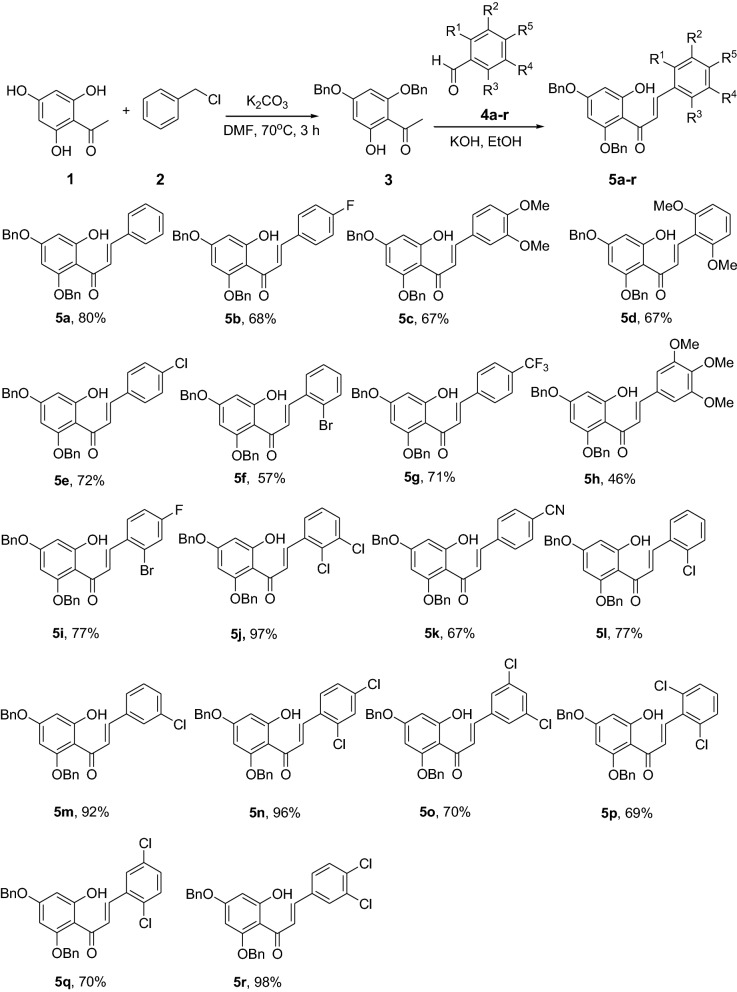
Synthesis of chalcones **5a-r**

**Fig. 2:**
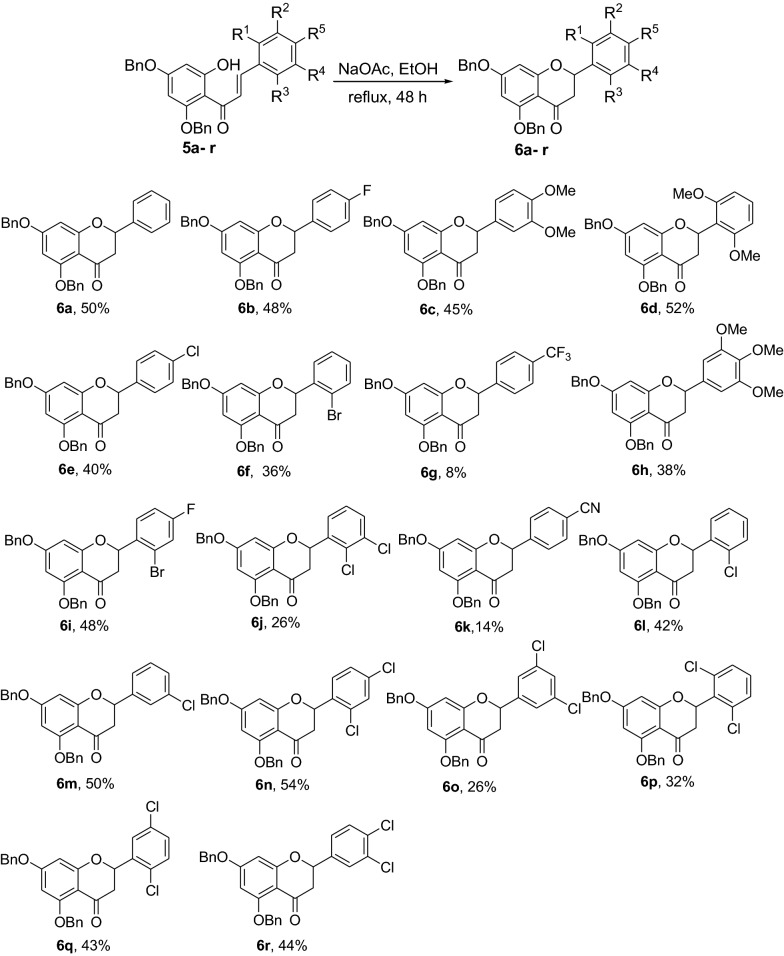
Synthesis of benzylated flavanones **6a-r**

The synthesis of various chalcones was performed after protection of trihydroxyacetophenone (**1**) with benzyl chloride (**2**) using potassium carbonate as base in DMF (Fig. 1) [10]. The product **3** was obtained as a white solid in 79% yield. In the next step, the desired chalcones **5a-r** were prepared via Claisen-Schmidt condensation [11, 12] of 2,4-dibenzyloxy-6-hydroxyacetophenone (**3**) with various aromatic aldehydes **4a-r** with electron deficient or electron donating properties using KOH as base in EtOH. The chalcones **5a-r** was isolated from 46 to 98% in yields (Fig. 2). The lowest yields were obtained with 2-bromobenzaldehyde **4f** and 3,4,5-trimethoxybenzaldehyde **4 h** as substrates. Low yields of chalcones with benzaldehydes containing a methoxy group in the substrate are documented in the literature [13].

Having secured the synthesis of the intended chalcones, we focused our attention towards the synthesis of pinocembrin and its analogs. Previous work has shown that the cyclization of chalcones to flavanones can be effected employing various methods using acids, bases and celite supported potassium fluoride in methanol [14, 15]. In the present work, the transformation of chalcones to flavanones was attempted with acetic acid, methane sulphonic acid and sodium acetate as catalysts. The latter reagent was found to deliver the desired products in higher yields than with the two other reagents. Upon refluxing the chalcones **5a-r** (1 mmol) with sodium acetate (8 mmol) in ethanol, they were transformed into an equilibrium mixture made up of the chalcones **5a-r** and the flavanones **6a-r** (Fig. 2). This is evident as approximately 40% of the starting material chalcone was also recuperated. Therefore, the low yields of the flavanones obtained in the present work can therefore be attributed to the reversibility of the chalcone cyclization. This is in agreement with the finding reported by Urgaonkar et al., (2005) [16].

**Fig. 3:**
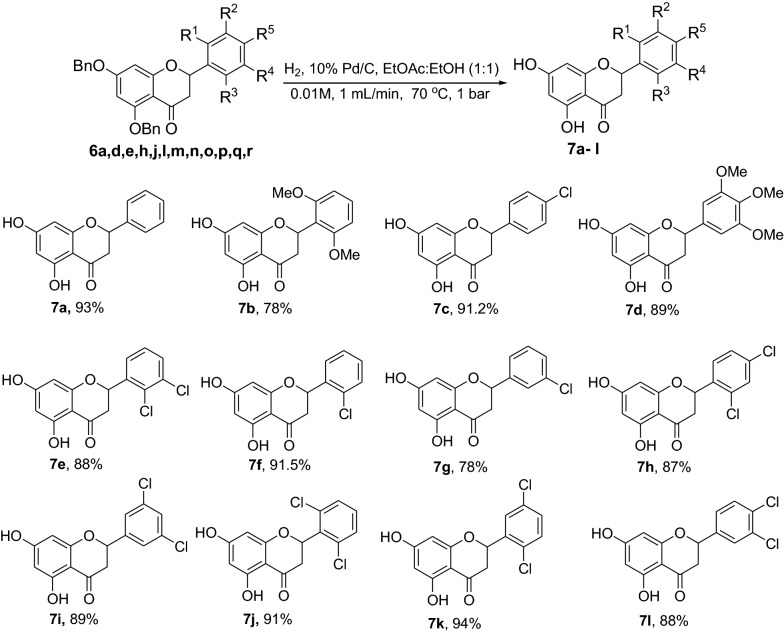
Synthesis of flavanones **7a-l**

Compounds **6a-e** and **6i** were subjected to in vivo antiplasmodial activity with the better result achieved with compounds **6a** and **6e**. The activity displayed by the latter compound might be due to the presence of a chlorine atom. The antiplasmodial activity of dibenzylated pinocembrin **6a** was compared with pinocembrin (**7a**). The result showed a dramatic decrease in activity of **6a** compared with **7a**. This is probably accounted to the presence of benzyl group. Hence, we found it necessary to undertake the debenzylation of the pinocembrin analogs. To achieve this goal, the debenzylation of the dibenzylated flavanones **6a-r** (Fig. 3) was achieved by catalytic hydrogenation using an H-Cube. To find out optimal parameters for the deprotection, different solvents including EtOH, CHCl_3_, EtOAc and EtOH:EtOAc (1:1) were tested using the conversion of **6a** to **7a** as an example. The latter solvent system was found efficient in furnishing the product in 93% yield. The reaction temperature of the deprotection was also optimized. It was found that the debenzylation of both benzyl groups occurred at 70 °C while the undesired monobenzylated product was obtained at 60 °C exclusively. Hence, the debenzylation of compounds **6b-r** to pinocembrin analogs **7b-r** was performed using an H-Cube Pro with 10% Pd/C as catalyst and a flow rate of 1 mL/min at 70 °C and 1 bar. This method was found highly attractive as it furnished the corresponding debenzylated products in yields ranging between 78 and 94% and in short reaction times (Fig. 3). The use of the H-Cube Pro has also eliminated the dangers associated with hydrogenation by generating hydrogen in situ and the handling of pyrophoric catalysts by filling them in sealed catalyst cartridges. This is advantageous over the conventional debenzylation protocol which succeeded to give the desired product neither with Pd/C (5%, 10% and Pd black) nor Pd(OH)_2_/C (20%) under various conditions (Up to 15 kg hydrogen pressure and 70 °C in temperature and varieties of solvents) [17].

The structures of the compounds synthesized in the present work were characterized using various spectroscopic methods, including UV, IR and NMR as well as by mass spectrometry.

### Antiplasmodial activity of pinocembrin and its dibenzylated analogs

The in vivo antiplasmodial activities of pinocembrin (**7a**) and some of its dibenzylated analogs (**6a, 6b, 6c, 6e** and **6i**) were evaluated at different doses using Peter’s four day suppressive assay against *P. berghei* infected mice [18]. The ability to reduce parasitaemia density is an indicator of the presence of antimalarial activities in a sample [19]. A drug/sample which suppresses the parasitaemia beyond 30% is considered as active against the parasites [20]. In view of this, pinocembrin and its analogs showed antiplasmodial activities against *P. berghei* infected mice as evidenced from the percentage of parasite inhibition (Table 1). Pinocembrin (**7a**) had shown significant in vivo antiplasmodial activities compared with both control groups (P < 0.05). It inhibits the parasitaemia by 74.4% and 87.2% at 20 mg/kg and 30 mg/kg, respectively. The result obtained herein is superior to the suppression of parasitaemia observed for pinocembrin isolated from *D. angustifolia* which inhibited the parasites by 80.0% at 40 mg/kg [9]. A group of mice treated by **6b** and **6e** showed 25.5% and 55.6% suppression of parasitaemia at a dose 20 mg/kg and the results were turned out to be significant compared with the NC group (P < 0.05).

**Table 1:** In vivo suppressive effect of pinocembrin and its analogs against *P. berghei* in *Swiss albino* mice

No.	Doses (mg/kg)	Parasitological effect		MST ± SEM	Body Weight ± SEM	
		%Parasitaemia ± SEM	%Suppression		D0	D4
**6a**	NC	59.00 ± 0.78^a^	0.00	7.00 ± 0.32	24.00 ± 0.63	22.92 ± 0.84
	15	57.00 ± 1.28^b^	21.69 ^b^	9.60 ± 0.40	24.38 ± 0.91	23.38 ± 0.93
	20	40.00 ± 0.79 ^bc^	32.20 ^bc^	10.40 ± 0.87	22.98 ± 0.80	22.38 ± 0.57
	25	46.92 ± 1.17^c^	35.92^c^	12.00 ± 0.55	23.90 ± 0.78	23.36 ± 0.54
	30	38.08 ± 0.56^c^	38.04^c^	13.60 ± 0.87	23.44 ± 0.63	22.72 ± 0.53
	35	34.80 ± 0.86^d^	49.09^d^	11.60 ± 0.40	23.70 ± 0.56	22.87 ± 0.69
**6b**	NC	39.85 ± 0.80^a^	0.00	7.60 ± 0.25	24.38 ± 0.56	23.60 ± 0.58
	20	29.62 ± 1.21^b^	25.52^b^	7.60 ± 0.68	23.92 ± 0.74	22.74 ± 0.64
**6c**	NC	77.40 ± 1.60^a^	0.00	6.80 ± 0.66	23.68 ± 0.98	22.26 ± 0.93
	20	51.50 ± 1.14^b^	33.46^b^	11.80 ± 0.37	23.44 ± 0.78	22.74 ± 0.87
**6d**	NC	39.85 ± 0.80a	0.00	7.60 ± 0.25	24.38 ± 0.56	23.60 ± 0.58
	30	36.23 ± 2.20a	8.90 a	6.60 ± 0.30	24.32 ± 0.45	22.60 ± 0.65
**6e**	NC	59.00 ± 0.78^a^	0.00	7.00 ± 1.28	24.00 ± 0.63	22.92 ± 0.84
	20	26.14 ± 0.93^b^	55.57^b^	14.60 ± 0.51	24.52 ± 0.58	24.52 ± 0.56
**6i**	NC	73.22 ± 1.25^a^	0.00	7.80 ± 0.37	23.66 ± 0.57	22.04 ± 0.46
	15	60.46 ± 0.78^b^	17.42^b^	8.80 ± 0.49	23.06 ± 0.60	22.06 ± 0.59
	25	51.30 ± 0.44^c^	29.05^c^	8.00 ± 0.45	23.12 ± 0.47	22.90 ± 1.02
	30	43.58 ± 0.62^d^	40.45^d^	13.6 ± 1.03	23.70 ± 0.68	23.50 ± 0.65
**7a**	NC	33.25 ± 1.16^a^	0.00	7.40 ± 0.51	24.24 ± 0.64	23.50 ± 0.65
	20	8.5 0 ± 0.41^b^	74.41^b^	13.60 ± 1.25	23.70 ± 0.47	23.30 ± 0.49
	30	4.26 ± 0.13^c^	87.19^c^	14.40 ± 0.87	24.54 ± 0.58	24.06 ± 0.63
C**Q**^1^	30	0.00	100.00	21 <		

***NC*** Negative control; Means with different letters are significantly different (P < 0.05); P-value is set for the comparison between treated and NC groups; CQ: Chloroquine; ^1^has been used as positive control for the test of all compounds

In some cases, compounds isolated from natural products may show instability on exposure to high temperature, hence synthesizing analogs could help to increase the chemical stability and biological activities of the compounds. However, the results in the present study showed that the benzylated analogs exhibited less biological activity compared with pinocembrin (**7a**). Therefore, this study is parallel with the work of Werbel and Degnan (1987) in which the original quinazoline amino group depicted a better antiplasmodial activity compared with its analogs [21]. In contrast, Silva et al. [22] reported that the analogs of 4-nerolidylcatechol showed a better parastaemia inhibition effect (72% suppression at dose 600 mg/kg b. wt) compared with isolated compound (60% suppression at dose 600 mg/kg b. wt) [22]. Furthermore, flavanone**7a** inhibits the parasites in a dose dependent manner with the higher dose causing a higher parastaemia inhibition (Table 1). This is in close agreement with the work of Silva et al. [23] whereby some indole alkaloids displayed better activity as the dose increases [23].

Significant (p < 0.05) parasitaemia suppression was also observed on mice treated with compound **6i** with percent inhibition of 40.4% at 35 mg/kg with the effect statistically significant compared with NC (P < 0.05). At day four of post infection, compound **6c** exerted 33.5% parasitaemia inhibition. The parasitaemia suppression in all treated groups was significant (P < 0.05) compared with the NC groups.

Body weight loss is another common feature in *P*. *berghi* infected mice with the effect reduced by an effective antimalarial agent. A significant body weight reduction was not observed in most of the group treated with the samples (Table 1) compared with NC groups (p < 0.005). Compound **6e**, **6a** (20, 25 and 30 mg/kg b. wt), **6i** (25 and 35 mg/kg), **6c** and **7a** were shown to be significant in preventing body weight reduction (p < 0.005) of the mice (Table 1). However, significant body weight reduction was observed on mice treated with 6i and 6a at dose 15 mg/kg indicating that these two compounds couldn’t prevent a body weight loss as the dose decreases. Furthermore, those treated with compound **6a** at dose 35 mg/kg (highest given dose) also showed significant body weight reduction (p < 0.05). Thus, this compound likely has a property that causes depression and loss of appetite following the dose increment.

A material which induces longer survival time in *P*. *berghei* infected mice compared with the NC group is considered as a good antimalarial agent [24]. In this study, mice treated with pinocembrin and its benzylated analogs showed prolonged MST compared with the NC groups (P < 0.05) indicating that the samples suppressed *P. berghei* and reduced the overall pathogenic effect of the parasite. The survival time in compound **7a** treated groups were found to be in a dose dependent manner with the group treated in a higher dose (30 mg/kg) survived longer (14.40 days) compared with the one treated at 20 mg/kg which survived for 13.60 days. A better MST was observed in a group treated with **6e** (14.60) at dose 20 mg/kg. These two compounds also induced a better parasitaemia inhibition compared with the other compounds tested in this study. This result is in agreement with the work of Nardos and Mekonnen (2017) in which a group treated by hydro-ethanol crude extract of leaves of *Ajuga remota* at dose 100 mg/kg showed a higher parasitemia inhibition (77.54%) and with a longer survival time [25].

*P. berghei* infected mice suffer from anaemia because of RBC destruction, either by parasite multiplication or by spleen reticuloendotelial cell action as the presence of many abnormal RBC stimulates the spleen to produce many phagocytes [19]. An effective antimalarial agent prevents reduction of packed cell volume (PCV) which is caused by hemolysis of RBC following the rise of parasitaemia [26]. Those mice treated with most of the samples showed a slight decrease in PCV at D4 (Table 2). However those mice treated with **6e** at dose 30 mg/kg and **7a** at dose 20 mg/kg and 30 mg/kg showed no significant change in PCV reduction compared with the positive control indicating the ability of the samples in reducing the parasites.

**Table 2:** Effect of pinocembrin and its analogs on PCV of *P. berghei* infected mice

No.	Dose	PCV ± SME		% Change	P value
		D 0	D 4		
**6a**	NC	51.11 ± 0.63	48.47 ± 0.42	− 5.16	0.01
	15	51.25 ± 0.21	50.83 ± 0.52	− 0.82	0.10
	25	51.13 ± 0.15	49.93 ± 0.41	− 2.35	0.06
	30	51.02 ± 0.13	50.04 ± 0.31	− 1.92	0.10
	35	50.75 ± 0.24	50.55 ± 0.32	− 0.40	0.30
**6b**	NC	51.38 ± 0.25	48.76 ± 0.40	− 5.10	0.01
	20	51.20 ± 0.20	50.19 ± 0.27	− 2.00	0.08
**6c**	NC	51.11 ± 0.63	48.47 ± 0.42	− 5.16	0.01
	20	50.84 ± 0.40	51.00 ± 0.31	0.32	0.31
**6d**	NC	51.38 ± 0.25	48.76 ± 0.31	− 5.10	0.01
	30	51.16 ± 0.35	49.79 ± 0.40	− 2.67	0.04
**6i**	NC	51.38 ± 0.31	47.98 ± 0.56	− 6.62	0.00
	15	51.09 ± 0.19	49.89 ± 0.24	− 2.35	0.06
	25	50.78 ± 0.29	50.00 ± 0.35	− 1.54	0.10
	35	51.26 ± 0.21	51.09 ± 0.23	− 0.33	0.37
**7a**	NC	50.98 ± 0.64	49.68 ± 0.20	− 2.55	0.04
	20	50.38 ± 0.40	50.66 ± 0.45	0.56	0.21
	30	50.76 ± 0.16	51.00 ± 0.12	0.47	0.30

### Molecular docking simulation of compounds 7a-i against *Plasmodium falciparum* dihydrofolate reductase-thymidylate synthase (PfDHFR-TS) (PDB ID 1J3I)

*P. falciparum *has an unmatched track record of gaining resistance to drugs currently existing in the market. Hence, it is necessary to search for compounds that can halt enzyme that play a key role in the biosynthesis of precursors for the DNA of the parasites. One of the enzymes responsible for the production of folates as well as thymidylate required for DNA synthesis is *P. falciparum* dihydrofolate reductase-thymidylate synthase [27]. This enzyme is a key target for searching for antimalarial drugs. Molecular docking is used in drug design because of its ability to substantially predict the conformation of ligands within the appropriate target binding site [28]. Among the synthesized dibenzylated analogs of pinocembrin in this work, the chloroderivatives exhibited better antiplasmodial activity. Hence in the present investigation, a molecular docking study was carried out using AutoDock Vina in order to elucidate which of the chloro derivatives of pinocembrin has the best binding affinity against the *P. falciparum* dihydrofolate reductase-thymidylate synthase. The synthesized compounds **7a-l** were found to have minimum binding energy varied from − 8.1 to − 8.4 kcal/mol (Table 3). The results demonstrated that the compounds have a better docking affinity within the binding pocket of PfDHFR-TS than the standard drug. The key amino acid residues within the active sites of PfDHFR-TS are Ala16, Ser-108, Phe-58, Asp-54, Ile-14, Met-55, Trp-48, and Thr-185. The compounds **7a**, **7b**, **7c**, **7d**, **7f**, **7g**, **7i**, **7j** and **7l** have shown at least one hydrogen bonding interaction within the active site of PfDHFR-TS with the key amino acid residues. The compounds **7e** (Gly-44), **7h** (Ser-111, Ser-167), **7k** (Gly-44 Ser-167), and **7l** (Ser-167) displayed hydrogen bond interaction with non-key residual amino acids within the active site of PfDHFR-TS. On the other hand, compound **7i** had no hydrogen bonding interaction with residual amino acids. Overall, the in silico docking analysis revealed that the synthesized compounds shown better binding affinity compared with chloroquine used as standard clinical drug indicating that compounds **7a-l** are potential antimalarial agents. The binding affinity, H-bond and hydrophobic, pi-cation and Van der Waals interactions of the synthesized compounds were summarized in Table 3.

**Table 3:** Molecular docking simulation of compounds 7a-l against *P. falciparum* dihydrofolate reductase-thymidylate synthase

No.	Formula	Binding affinity (Kcal/mol)	H-bond	Residual interactions	
				Hydrophobic/Pi-Cation	Van der Waals
**7a**	C_15_H_12_O_4_	− 8.1	Ala-16	Leu-46, Leu-40, Phe-58, Met-55, Leu-119, Ile-112,	Cys-15, Ser-167, Gly-166, Tyr-170
**7b**	C_17_H_16_O_6_	− 8.1	Asp-54, Ala-16	Leu-40, Leu-46, Phe-58, Met-55	Cys-15, Gly-44, Val-45, Ile-164, Ser-111, Gly-41, Ser-167, Tyr-170
**7c**	C_15_H_11_ClO_4_	− 8.2	Ser-111, Ser-167, Ile-164	Ala-16, Ile-14, Cys-15, Leu-40, Phe-58	Asp-54, Gly-44, Ser-108, Gly-41, Gly-166, Tyr-170
**7d**	C_18_H_18_O_7_	− 8.2	Ala-16, Leu-40, Ser-108, Asn-42, Gly-44, Thr-107	Leu-46, Ile-112, Val-195	Lys-43, Val-45, Gly-41, Val-168, Ser-167, Ile-164, Tyr-170
**7e**	C_15_H_10_Cl_2_O_4_	− 8.2	Gly-44	Phe-58, Ser-167	Ala-16, Asp-54, Ile-164, Ser-108, Thr-107, Val-45, Gly-166, Tyr-170
**7f**	C_15_H_11_ClO_4_	− 8.4	Ser-111, Ile-164	Ala-16, Cys-15, Leu-46, Trp-48, Leu-40	Ile-14, Phe-58, Asp-54, Ser-108, Gly-44, Gly-166, Ser-167, Val-195, Tyr-170
**7g**	C_15_H_11_ClO_4_	− 8.3	Ser-108, Ser-111, Ser-167	Ala-16, Leu-40, Phe-58, Met-55	Asp-54, Cys-15, Leu-46, Thr-107, Gly-41, Gly-166
**7h**	C_15_H_10_Cl_2_O_4_	− 8.3	Ser-111, Ser-167	Ala-16, Leu-46, Trp-48, Ile-14, Cys-15, Phe-58, Leu-40	Gl-41, Gly-166, Ser-108, Ile-164, Thr-185, Tyr-170
**7i**	C_15_H_10_Cl_2_O_4_	− 8.3	–	Ala-16, Phe-58, Ser-167	Asp-54, Leu-46, Cys-15, Ser-108, Ser-111, Gly-44, Thr-107, Val-195, Gly-166, Leu-40, Ile-164, Tyr-170
**7j**	C_15_H_12_O_4_	− 8.1	Ser-108, Ile-164	Val-195, Thr-107	Asn-42, Val-45, Gly-41, Ser-111, leu-40, Gly-165, Gly-166, Ser-167, Val-168
**7k**	C_17_H_16_O_6_	− 8.4	Gly-44, Ser-167	Ala-16, Leu-46, Phe-58, Ile-112, Leu-119, Ile-164	Ser-108, Ser-111, Thr-107, Val-45, Val-195, Ggly-166, Tyr-170
**7l**	C_15_H_11_ClO_4_	− 8.4	Ser-167	Ala-16, Leu-46, Phe-58, Ile-165	Cys-15, Asp-54, Ser-108, Ser-111, Gly-166, Tyr-170
**CQ**	C_18_H_26_ClN_3_	− 7.2	Ile-164	Ala-16, Ile-14, Phe-58	Asp-54, Trp-48, Cys-15, Leu-46, Ser-108, Ser-111, Val-45, Gly-44, Asn-42, Gly-41, Leu-40, Gly-166, Gly-165, Tyr-170

**Fig. 4:**
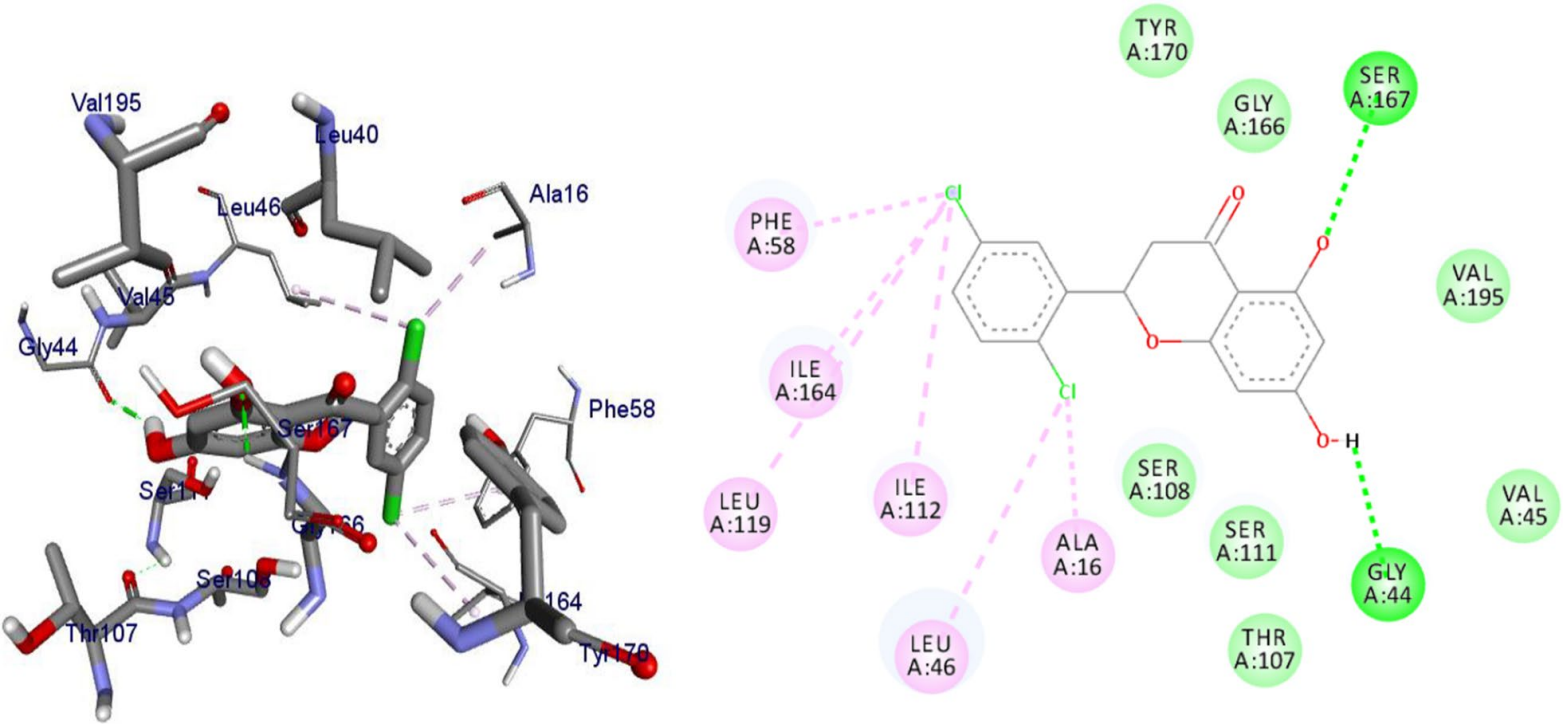
2D and 3D binding interactions of **7k** against *Plasmodium falciparum* dihydrofolate reductase-thymidylate synthase

**Fig. 5:**
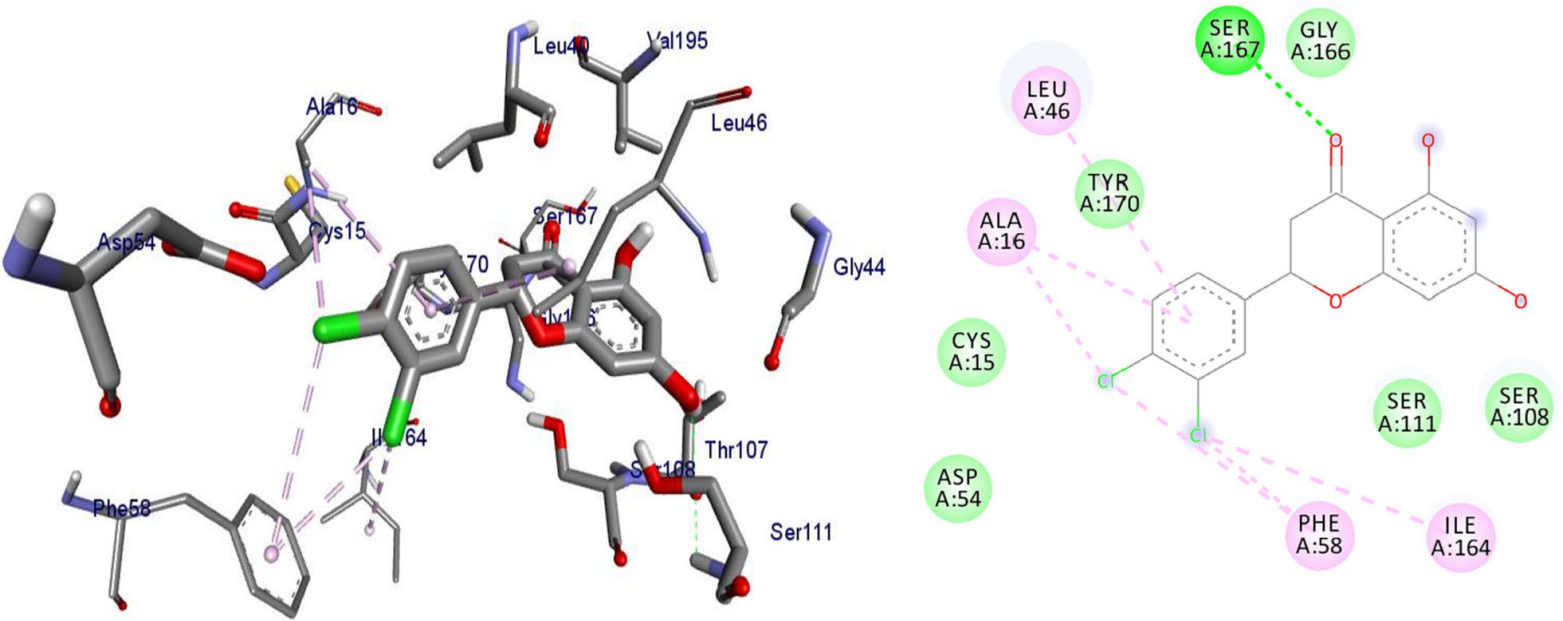
2D and 3D binding interactions of **7l** against *Plasmodium falciparum* dihydrofolate reductase-thymidylate synthase

**Fig. 6:**
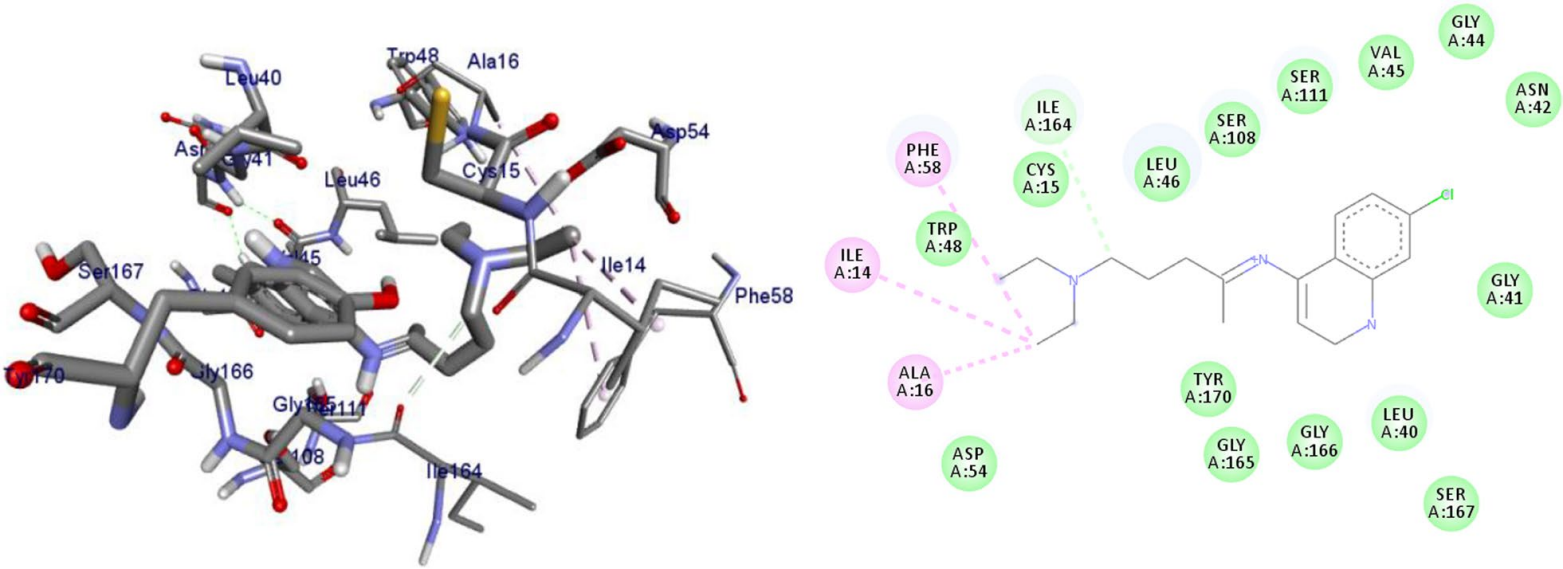
2D and 3D binding interactions of CQ against *Plasmodium falciparum* dihydrofolate reductase-thymidylate synthase

The 2D and 3D binding interactions of compounds **7k, 7l** and chloroquine against *P. falciparum* dihydrofolate reductase-thymidylate synthase were depicted in Figs. 4, 5, 6. Ribbon model shows the binding pocket structure of PfDHFR-TS with compounds **7f, 7k and 7l**. Hydrogen bonds between compounds and amino acids are shown as green dash lines, hydrophobic interactions are shown as pink lines. The molecular docking analysis of compounds **7a-j** are given as supplementary material (Additional file 2).

### In silico pharmacokinetics (drug-likeness) properties

In order to get a potential drug for drug development, forecasting of ADME (absorption, distribution, metabolism and excretion) profiles of compounds including their pharmacokinetic and drug-like properties using Swiss ADME is vital. In this investigation, compounds **7a-l** were assessed for their drug-like properties using SwissADME. The results indicates that compounds **7a-l** satisfy Lipinski's rule of five with zero violations (Table 4) [29]. The Kp values of the synthesized compounds were within the range of − 5.29 to − 6.35 cm/s while it was − 4.96 cm/s for chloroquine inferring low skin permeability (Table 5). The predicted logP values of the compounds synthesized also indicate that all the compounds had optimal lipophilicity (ranging from 2.11 to 2.9). This was inferior to chloroquine which had logP value of 3.95. The SwissADME prediction parameters showed that all the compounds have high GI absorption, high blood brain barrier (BBB) permeation (except compounds **7b** and **7d**) and compound **7d** is a substrate of permeability glycoprotein (P-gp) (Table 5). Overall, these prediction results indicate that the compounds **7e, 7f, 7h, 7i, 7j** and **7l** can be better active pharmacological agent compared to the other reported compounds in this study. This is in agreement from the observed good binding affinity of these compounds with *P. falciparum* dihydrofolate reductase-thymidylate synthase.

**Table 4:** Drug-likeness predictions of compounds 7a-l computed by SwissADME

S. No.	Formula	Mol.Wt. (g/mol)	NHD	NHA	NRB	TPSA (A^°2^)	LogP (cLogP)	Lipinski’s rule of Five Violation
7a	C_15_H_12_O_4_	256.25	2	4	1	66.76	2.11	0
7b	C_17_H_16_O_6_	316.31	2	6	3	85.22	2.71	0
7c	C_15_H_11_ClO_4_	290.7	2	4	1	66.76	2.37	0
7d	C_18_H_18_O_7_	346.33	2	7	4	94.45	2.9	0
7e	C_15_H_10_Cl_2_O_4_	325.14	2	4	1	66.76	2.43	0
7f	C_15_H_11_ClO_4_	290.7	2	4	1	66.76	2.36	0
7g	C_15_H_11_ClO_4_	290.7	2	4	1	66.76	2.39	0
7h	C_15_H_10_Cl_2_O_4_	325.14	2	4	1	66.76	2.58	0
7i	C_15_H_10_Cl_2_O_4_	325.14	2	4	1	66.76	1.91	0
7j	C_15_H_12_O_4_	325.14	2	4	1	66.76	2.53	0
7k	C_17_H_16_O_6_	325.14	2	4	1	66.76	2.57	0
7l	C_15_H_11_ClO_4_	325.14	2	4	1	66.76	2.56	0
CQ	C_18_H_26_ClN_3_	319.87	1	2	8	28.16	3.95	0

*NHD* Number of Hydrogen donor, *NHA* Number of Hydrogen acceptor, *NRB* Number of rotatable bonds, *TPSA* total polar surface area, *CQ* chloroquine

**Table 5:** ADME predictions of compounds 7a-l computed by SwissADME and PreADMET

No.	Chemical Formula	Skin Permeation Value (log Kp) cm/s	GI Absorption	BBB Permeability	Inhibitor Interaction (SwissADME/PreADMET)					
					P-gp substrate	CYP1A2 inhibitor	CYP2C19 inhibitor	CYP2C9 inhibitor	CYP2D6 inhibitor	CYP3A4 inhibitor
**7a**	C_15_H_12_O_4_	− 5.82	High	Yes	No	Yes	Yes	No	No	No
**7b**	C_17_H_16_O_6_	− 6.23	High	No	No	Yes	No	Yes	No	Yes
**7c**	C_15_H_11_ClO_4_	− 5.59	High	Yes	No	Yes	Yes	Yes	Yes	Yes
**7d**	C_18_H_18_O_7_	− 6.35	High	No	Yes	Yes	No	Yes	No	Yes
**7e**	C_15_H_10_Cl_2_O_4_	− 5.29	High	Yes	No	Yes	Yes	Yes	Yes	Yes
**7f**	C_15_H_11_ClO_4_	− 5.52	High	Yes	No	Yes	Yes	Yes	Yes	Yes
**7g**	C_15_H_11_ClO_4_	− 5.52	High	Yes	No	Yes	Yes	Yes	Yes	Yes
**7h**	C_15_H_10_Cl_2_O_4_	− 5.35	High	Yes	No	Yes	Yes	Yes	Yes	Yes
**7i**	C_15_H_10_Cl_2_O_4_	− 5.29	High	Yes	No	Yes	Yes	Yes	Yes	Yes
**7j**	C_15_H_12_O_4_	− 5.35	High	Yes	No	Yes	Yes	Yes	Yes	Yes
**7k**	C_17_H_16_O_6_	− 5.29	High	Yes	No	Yes	Yes	Yes	Yes	Yes
**7l**	C_15_H_11_ClO_4_	− 5.45	High	Yes	No	Yes	Yes	Yes	Yes	Yes
**CQ**	C_18_H_26_ClN_3_	− 4.96	High	Yes	No	Yes	No	No	Yes	Yes

*CQ* Chloroquine, *GI* Gastro-Intestinal, *BBB* Blood Brain Barrier, *P-gp* P-glycoprotein, *CYP* Cytochrome-P

## Experimental

### General

All reagents used in the present work were used without further purification. Glassware used was dried for 24 h at 120 °C in an oven. Solvents used in reactions were distilled over appropriate drying agents prior to use while those used for extraction and purification were distilled prior to use. Thin-layer chromatography (TLC) was performed on precoated aluminum plates (silica gel Merck 60 F) and visualized under UV light (254 nm) and/or by dipping in vanillin/H_2_SO_4_ followed by heating. Products were purified by column chromatography over silica gel (MN 60, 0.04–0.063 mm; Marcherey & Nagel). Melting points were determined in capillary tubes with a digital electrothermal melting point apparatus. IR spectra were measured on a Bruker Alpha FT-IR spectrometer. UV/Vis spectra were recorded on a Cary 4E spectrophotometer. ^1^H and ^13^C NMR spectra were recorded at 300 (75) MHz and 500 (125) MHz on Varian Unity Inova spectrometers. Coupling constants *J* [Hz] were directly taken from the spectra and are not averaged. Splitting patterns are designated as s (singlet), d (doublet), t (triplet), q (quartet), and m (multiplet). Low-resolution electron impact mass spectra [MS (EI)] and exact mass electron impact mass spectra [HRMS (EI)] were obtained at 70 eV using a double focusing sector field mass spectrometer Finnigan MAT 95, for the measurement of exact mass electrospray ionization mass spectra [ESI (HRMS)] a Bruker Daltonik spectrometer micrOTOF-Q was used.

### Synthesis and characterization of starting materials and chalcones

#### Synthesis and analytical data of 2,4-dibenzyloxy-6-hydroxyacetophenone (3) [10]


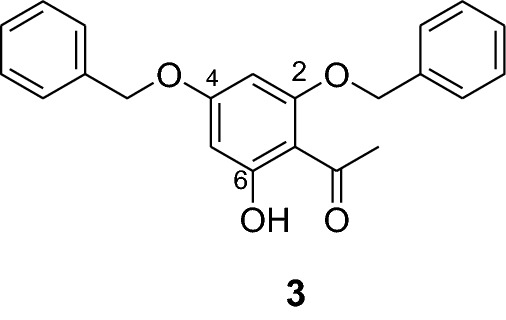


A 250 mL 2-neck round bottom flask was charged with *N*,*N*-dimethylformamide (24 mL) under argon, heated to 35 °C, then trihydroxyacetophenone (**1**) (5 g, 29.7 mmol) was added in one portion, followed by more DMF (16 mL). Potassium carbonate (8.5 g, 55.9 mmol) was added and the mixture was heated to 65 °C. Benzyl chloride (**2**) (7.5 g, 59.4 mmol) was added in one portion and the mixture was heated to 70 °C for 3 h, cooled to room temperature and filtered. The filter cake was rinsed with dichloromethane. The combined filtrates were concentrated *in vacuo* and the residual orange oil was taken up in dichloromethane (50 mL), stirred for five min, and filtered. The filtrate was column filtered over silica gel with dichloromethane as eluent. It was concentrated, dichloromethane (5 mL) and cyclohexane (8 mL) were added and the mixture was stirred for 20 min. The resultant white crystalline solid was collected by suction filtration, washed with cyclohexane and air dried to yield 5.7 g (79%) of a white crystalline solid which was identified as 2,4-dibenzyloxy-6-hydroxyacetophenone (**3**). ^1^H NMR (CDCl_3_, 300 MHz) *δ* 2.56 (3H, s, H-2’), 5.06 (4H, br s, benzylic H), 6.09 (1H, d, *J* = 2.3 Hz, H-3), 6.16 (1H, d, *J* = 2.3 Hz, H-5), 7.34–7.43 (10H, m, aromatic H), 14.01 (1H, s, OH); ^13^C NMR (CDCl_3_, 75 MHz) *δ* 33.1 (C-2’), 70.3 (C-1’’’), 71.1 (C-1’’), 92.3 (C-5), 94.6 (C-3), 106.3 (C-1), 127.6 (C-2’’’, C-6’’’, C-4’’’,), 127.9 (C-2’’’’, C-4’’’’, C-6’’’’), 128.3 (C-3’’’, C-5’’’), 128.4 (C-3’’’’, C-5’’’’), 135.5 (C-1’’’), 135.8 (C-1’’’’), 161.9 (C-2), 165.0 (C-6), 167.5 (C-4), 203.2 (C-2’).

#### General procedure I for the synthesis of chalcones 5a-r [30]

2,4-Dibenzyloxy-6-hydroxyacetophenone (**3**) (2.8 mmol) was condensed with substituted benzaldehydes **4a-r** (2.8 mmol) in the presence of aqueous KOH (60%, 1.5 mL) in ethanol (8.5 mL). The reaction mixture was stirred for 24 h at room temperature and poured into water (30 mL). After neutralization with 10% HCl, ethanol was removed using a rotary evaporator and the reaction mixture was extracted with EtOAc (3 × 30 mL). The combined organic phases were dried over anhydrous MgSO_4_, filtered and concentrated in vacuo. Column chromatography of the crude product on silica gel using petroleum ether:EtOAc (4:1) as eluent furnished the corresponding chalcones **5a-r** as yellow solids. Compounds **5a-r** were synthesized according to general procedure I.

#### Synthesis and analytical data of 2’,4’-dibenzyloxy-6’-hydroxychalcone (5a)


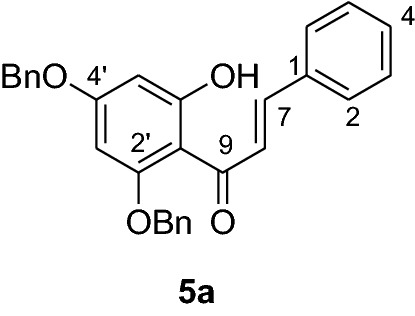


According to general procedure I, 2,4-dibenzyloxy-6-hydroxyacetophenone (**3**) (1 g, 2.8 mmol) was condensed with benzaldehyde (**4a**) (0.3 g, 2.8 mmol) in the presence of aqueous KOH (60%, 1.5 mL) in ethanol (8.5 mL). The product **5a** was obtained in 80% yield as a yellow solid. mp 124–125 °C); UV (MeOH) λ_max_: 278, 345 nm; ^1^H NMR (CDCl_3_, 300 MHz) *δ* 5.01 (2H, s, OCH_2_), 5.06 (2H, s, OCH_2_), 6.18 (1H, d, *J* = 2.3 Hz, H-5’), 6.23 (1H, d, *J* = 2.3 Hz, H-3’), 7.06 (2H, d, *J* = 7.6 Hz, H-2,6), 7.19 (3H, t, *J* = 8.1 Hz, H-3,4,5), 7.25–7.52 (10H, m, H-2’’– H-6’’, H-2’’’– H-6’’’), 7.71 (1H, d, *J* = 16 Hz, H-8), 7.89 (1H, d, *J* = 16 Hz, H-9), 14.50 (1H, s, OH); ^13^C NMR (CDCl_3_, 75 MHz) *δ* 70.3 (OCH_2_), 71.4 (OCH_2_), 92.5 (C-3’), 95.0 (C-5’), 106.3 (C-1’), 127.4 (C-8), 127.6 (C-2, C-6), 128.31 (C-2’’’, C-6’’’), 128.4 (C-2’’, C-6’’), 128.5 (C-4’’’), 128.62 (C-4’’), 128.64 (C-4), 128.7 (C-3’’’,C-5’’’), 128.8 (C-3, C-5), 128.9 (C-3’’,C-5’’),129.8 (C-1), 135.2 (C-1’’’), 135.3 (C-1’’), 135.8 (C-7), 161.7 (C-2’), 165.2 (C-6’), 168.8 (C-4’), 192.6 (C-9).

#### Synthesis and analytical data of 2’,4’-dibenzyloxy-6’-hydroxy-4-fluorochalcone (5b)


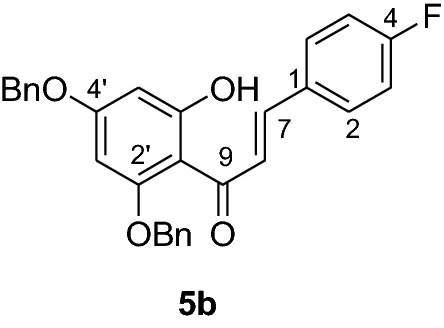


According to general procedure I, 2,4-dibenzyloxy-6-hydroxyacetophenone (**3**) (0.4 g, 1.6 mmol) was condensed with 4-fluorobenzaldehyde (**4b)** (0.19 g, 1.6 mmol) in the presence of aqueous KOH (60%, 1.5 mL) in ethanol (8.5 mL). The product was obtained in 68% yield. mp 126–127 °C; UV(MeOH) λ_max_: 276, 337 nm; IR *ν̃* [cm^−1^]: 1660 (conjugated C = O), 1571 (arom. C = C), and 1023 (C-O); ^1^H NMR (CDCl_3_, 300 MHz) *δ* 5.0 (2H, s, OCH_2_), 5.1 (2H, s), 5.56 (1H, s, OCH_2_), 6.18 (1H, d, *J* = 2.4 Hz, H-5’), 6.2 (1H, d, *J* = 2.4 Hz, H-3’), 6.8 (2H, m, H-3,H-5), 7.01 (2H, m, H-2,H-6), 7.50–7.39 (10H, m, H-2’’– H-6’’, H-2’’’– H-6’’’), 7.64 (1H, d, *J* = 15 Hz, H-8), 7.78 (1H, d, *J* = 15 Hz, H-7), 14.5 (1H, s, 6’-OH);^13^C NMR (CDCl_3_, 75 MHz) *δ* 70.3 (OCH_2_), 71.4 (OCH_2_), 92.5 (C-3’), 95.0 (C-5’), 106.2 (C-1’), 115.5 (C-3,C-5), 115.8 (C-8), 127.2 (C-2’’’, C-6’’’), 127.6 (C-2’’, C-6’’), 128.3 (C-4’’’), 128.7 (C-4’’), 128.9 (C-2, C-6), 129.3 (C-3’’’, C-5’’’), 130.2 (C-3’’, C-5’’), 131.5 (C-1), 132.7 (C-1’’’), 135.4 (C-1’’), 135.8 (C-7), 141.3 (d, C-4), 161.7 (C-2’), 165.3 (C-6’), 168.8 (C-4’), 192.4 (C-9).

#### Synthesis of 2’,4’-dibenyloxy-6’-hydroxy-3,4-dimethoxychalcone (5c)


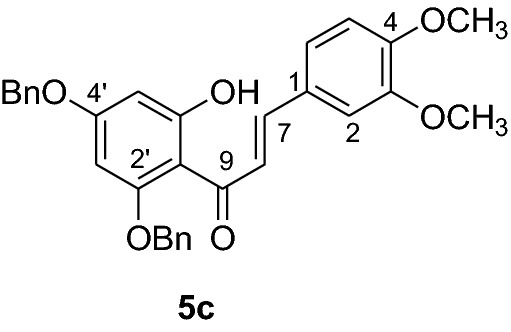


According to general procedure I, 2,4-dibenzyloxy-6-hydroxyacetophenone (**3**) (0.4 g, 1.1 mmol) was condensed with 3′4’-dimethoxybenzaldehyde (**4c**) (0.19 g, 1.1 mmol) in the presence of aqueous KOH (60%, 1.5 mL) in ethanol (8.5 mL). The product **5c** was obtained in 67% as a yellowish solid. mp 102–103 °C; UV(MeOH) λ_max_: 276, 331 nm; IR *ν̃* [cm^−1^]: 1677 (conjugated C = O), 1630 (C = C), and 1040 (C-O); ^1^H NMR (CDCl_3_, 300 MHz) *δ* 3.62 (1H, s, OMe), 3.91 (1H, s, OMe), 5.10 (4H, s, OCH_2_), 6.17 (1H, d, *J* = 2.4 Hz, H-5’), 6.23 (1H, d, *J* = 2.4 Hz, H-3’), 6.72 (1H, d, *J* = 9 Hz, H-5), 6.77 (1H, d, *J* = 3 Hz, H-2), 6.81 (1H, dd, *J* = 3 Hz and 9 Hz, H-6), 7.47–7.31 (10H, m, H-2’’’– H-6’’’, H-2’’– H-6’’), 7.70 (1H, d, *J* = 15.6 Hz, H-8), 7.80 (1H, d, *J* = 15.6 Hz, H-7), 14.4 (1H, s, 6’OH); ^13^C NMR (CDCl_3_, 75 MHz) *δ* 55.6 (OMe), 55.9 (OMe), 70.2 (OCH_2_), 71.1 (OCH_2_), 92.7 (C-3’), 95.0 (C-5’), 106.6 (C-1’), 110.8 (C-2), 110.9 (C-5), 122.4 (C-6), 125.5 (C-8), 127.6 (C-2’’’, C-6’’’), 127.8 (C-2’’, C-6’’), 128.2 (C-4’’’), 128.3 (C-4’’), 128.6 (C-1), 128.7 (3’’’, C-5’’’), 128.8 (C-3’’, C-5’’),, 135.7 (C-1’’’), 135.8 (C-1’’), 142.8 (C-7), 148.8 (C-4), 150.8 (C-3), 161.5 (C-2’), 165.0 (C-6’), 168.3 (C-4’), 192.5 (C-9).

#### Synthesis and analytical data of 2’,4’-dibenyloxy-6’-hydroxy-2,6-dimethoxychalcone (5d)


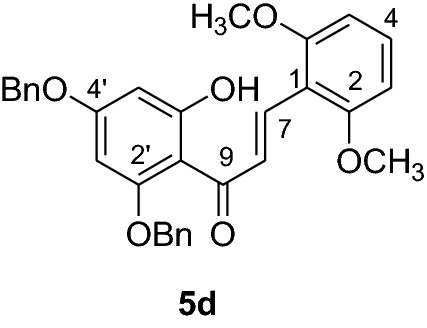


According to general procedure I, 2,4-dibenzyloxy-6-hydroxyacetophenone (3) (0.4 g, 1.1 mmol) was condensed with 3′4’-dimethoxybenzaldehyde (**4d**) (0.19 g, 1.1 mmol) in the presence of aqueous KOH (60%, 1.5 mL) in ethanol (8.5 mL). The product **5d** was obtained in 67%. mp 99–100 °C; UV(MeOH) λ_max_: 275, 332 nm; IR *ν̃* [cm^−1^]: 1671 (conjugated C = O), 1603 (C = C), 1560 (C = C), 1347, 1206, and 1027 (C-O); ^1^H NMR (CD_3_COCD_3_, 300 MHz) *δ* 3.6 (3H, s, OMe), 3.8 (3H, s, OMe), 5.22 (2H, s, OCH_2_), 5.29 (2H, s, OCH_2_), 6.23 (1H, d, *J* = 2.2 Hz, H-5’), 6.36 (1H, d, *J* = 2.2 Hz, H-3’), 6.96 (1H, m, H-4), 6.98 (2H, br d, H-3, H-5), 7.54–7.30 (10H, m, H-2’’’– H-6’’’, H-2’’– H-6’’), 14.1 (1H, s, 6’OH);^13^C NMR (CD_3_COCD_3_, 75 MHz) *δ* 55.8 (OMe), 56.3 (OMe), 70.8 (OCH_2_), 71.8 (OCH_2_), 93.5 (C-3’), 95.8 (C-5’), 107.3 (C-1’), 113.3 (C-3), 113.5 (C-5), 118.0 (C-1), 125.2 (C-8), 128.5 (C-2’’’, C-6’’’), 128.6 (C-2’’, C-6’’), 128.7 (C-4’’’), 128.8 (C-4’’), 128.9 (C-3’’’, C-5’’’), 128.91 (C-3’’, C-5’’), 129.3 (C-4), 129.4 (C-1’’’), 137.1 (C-1’’), 137.8 (C-7), 153.8 (C-2), 154.5 (C-6), 162.6 (C-2’), 166.2 (C-6’), 168.7 (C-4’), 193.7 (C-9).

#### Synthesis and analytical data of 2’,4’-dibenzyloxy-6’-hydroxy-4-chlorochalcone (5e)


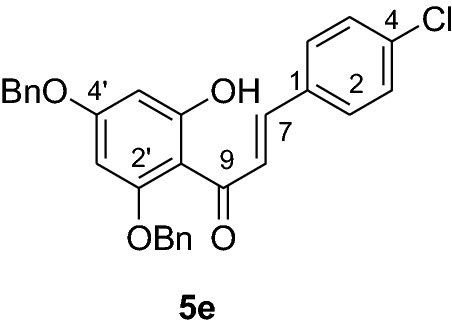


According to general procedure I, 2,4-dibenzyloxy-6-hydroxyacetophenone (**3**) (0.5 g, 1.4 mmol) was condensed with 4-chlorobenzaldehyde (**4e**) (0.21 g, 1.4 mmol) in the presence of aqueous KOH (60%, 1.5 mL) in ethanol (8.5 mL). The product **5e** was obtained as a white solid in 72% yield. mp 142–144 °C; *R*_f_ 0.63 (petroleum ether:EtOAc 4:1); UV(MeOH) λ_max_: 274, 336 nm; IR *ν̃* [cm^−1^]: 1613 (C = C), 1487, 1162, 1114, and 1023 (C-O); ^1^H NMR (CDCl_3_, 300 MHz) *δ* 5.05 (2H, s, OCH_2_), 5.11 (2H, s, OCH_2_), 6.18 (1H, d, *J* = 2.2 Hz, H-5’), 6.23 (1H, d, *J* = 2.2 Hz, H-3’), 6.91 (2H, d, *J* = 7.0 Hz, H-3, H-5), 7.13 (2H, d, *J* = 7.0 Hz, H-2, H-6), 7.4 (10H, m, H-2’’’– H-6’’’, H-2’’– H-6’’), 7.62 (1H, d, *J* = 15.4 Hz, H-8), 7.82 (1H, d, *J* = 15.4 Hz, H-7); ^13^C-NMR (CDCl_3_, 75 MHz) *δ* 70.3 (OCH_2_), 71.2 (OCH_2_), 92.6 (C-3’), 95.2 (C-5’), 106.2 (C-1’), 127.2 (C-8), 127.3 (C-2’’’, C-6’’’), 127.6 (C-2’’, C-6’’), 128.0 (C-4’’’), 128.3 (C-4’’), 128.7 (C-2, C-6), 128.8 (C-3, C-5), 128.9 (C-3’’’, C-5’’’), 129.4 (C-3’’, C-5’’), 133.8 (C-1), 135.3 (C-4), 135.5 (C-1’’’), 135.7 (C-1’’), 141.0 (C-7), 161.6 (C-2’), 165.4 (C-6’), 168.8 (C-4’), 192.3 (C-9).

#### Synthesis and analytical data of 2’,4’-dibenyloxy-6’-hydroxy-2-bromochalcone (5f)


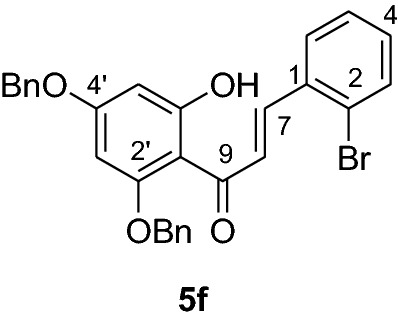


According to general procedure I, 2,4-dibenzyloxy-6-hydroxyacetophenone (**3**) (0.6 g, 1.7 mmol) was condensed with 2-bromobenzaldehyde (**4f**) (0.32 g, 1.7 mmol) in the presence of aqueous KOH (60%, 1.5 mL) in ethanol (8.5 mL). The product **5f** was obtained in 57% yield. mp 111–112 °C; UV(MeOH) λ_max_: 276, 338 nm; IR *ν̃* [cm^−1^]:_:_ 1673 (conjugated C = O), 1618 (C = C), 1552 (C = C), 1453, 1226, 1156, and 1021 (C-O); ^1^H NMR (CDCl_3_, 300 MHz) *δ* 5.04 (2H, s, OCH_2_), 5.10 (2H, s, OCH_2_), 6.17 (1H, d, *J* = 2.2 Hz, H-5’), 6.22 (1H, d, *J* = 2.2 Hz, H-3’), 6.96 (1H, t, *J* = 7.5 Hz, H-4), 7.14 (1H, dd, *J* = 7.2 Hz, 1.8 Hz, H-3), 7.41 (12H, m, H-5, H-6, H-2’’’– H-6’’’, H-2’’– H-6’’), 7.77 (1H, d, *J* = 15.4 Hz, H-8), 8.05 (1H, d, *J* = 15.4 Hz, H-7); ^13^C NMR (CDCl_3_, 75 MHz) *δ* 70.2 (OCH_2_), 71.2 (OCH_2_), 92.2 (C-3’), 95.1 (C-5’), 106.2 (C-1’), 125.8 (C-2), 127.4 (C-8), 127.6 (C-2’’’, C-6’’’), 128.3 (C-2’’, C-6’’), 128.5 (C-4’’’), 128.6 (C-4’’), 128.7 (C-5), 128.8 (C-6), 128.9 (C-3’’’, C-5’’’), 130.0 (C-3’’, C-5’’), 130.5 (C-4), 133.2 (C-3), 135.2 (C-1), 135.5 (C-1’’’), 135.7 (C-1’’), 140.5 (C-7), 161.6 (C-2’), 164.4 (C-6’), 168.7 (C-4’), 192.2 (C-9).

#### Synthesis of 2’,4’-dibenyloxy-6’-hydroxy-4-(trifluoromethyl)chalcone (5g)


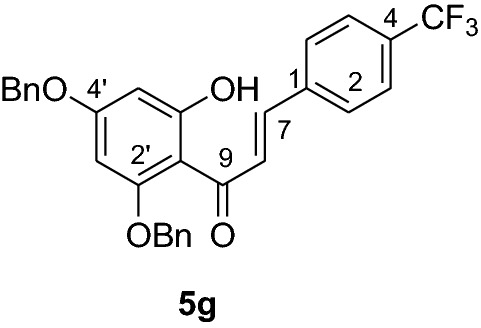


According to general procedure I, 2,4-dibenzyloxy-6-hydroxyacetophenone (**3**) (0.6 g, 1.7 mmol) was condensed with 4-(trifluoromethyl)benzaldehyde (**4 g)** (0.32 g, 1.7 mmol) in the presence of aqueous KOH (60%, 1.5 mL) in ethanol (8.5 mL). The product **5 g** was obtained in 71% yield. ^1^H NMR (CDCl_3_, 300 MHz) *δ* 5.05 (2H, s, OCH_2_), 5.10 (2H, s, OCH_2_), 6.18 (1H, d, *J* = 2.2 Hz, H-5’), 6.23 (1H, d, *J* = 2.2 Hz, H-3’), 7.09 (2H, d, *J* = 7.0, H-2, H-6), 7.48 (2H, d, *J* = 7.0 Hz, H-3, H-5), 7.42 (10H, m, H-2’’’– H-6’’’, H-2’’– H-6’’), 7.63 (1H, d, *J* = 15.4 Hz, H-8), 7.88 (1H, d, *J* = 15.4 Hz, H-7); ^13^C NMR (CDCl_3_, 75 MHz) *δ* 70.2 (OCH_2_), 71.3 (OCH_2_), 92.6 (C-3’), 95.0 (C-5’), 106.2 (C-1’), 125.4 (C-7), 125.5 (q, CF_3_), 127.6 (C-3, C-5), 128.2 (C-2, C-6), 128.5 (C-2’’’, C-6’’’), 128.7 (C-2’’, C-6’’), 128.9 (C-4’’’), 129.0 (C-4’’), 130.1 (C-3’’’, C-5’’’), 130.8 (C-3’’, C-5’’), 131.2 (C-4), 135.2 (C-1), 135.7 (C-1’’’), 138.7 (C-1’’), 140.2 (C-7), 161.8 (C-2’), 165.8 (C-6’), 168.8 (C-4’), 192.3 (C-9).

#### Synthesis of 2’,4’-dibenyloxy-6’-hydroxy-3,4,5-trimethoxychalcone (5h)


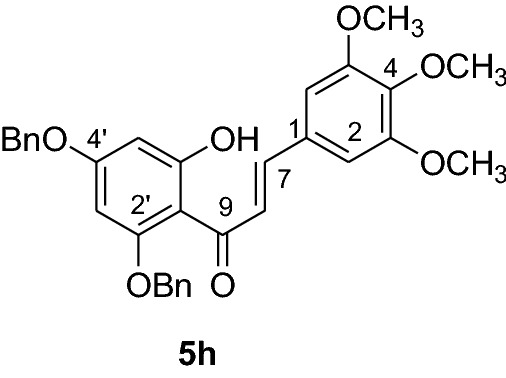


According to general procedure I, 2,4-dibenzyloxy-6-hydroxyacetophenone (**3**) (0.6 g, 1.7 mmol) was condensed with 3,4,5-trimethoxybenzaldehyde (**4 h**) (0.345 g, 1.7 mmol) in the presence of aqueous KOH (60%, 1.5 mL) in ethanol (8.5 mL). The product **5 h** was obtained in 46% yield. mp 94–96 °C; UV(MeOH) λ_max_: 275, 324 nm; IR *ν̃* [cm^−1^]: 1611 (C = C), 1577 (C = C), 1266, 1168, and 1095 (C-O); ^1^H NMR (CDCl_3_, 300 MHz) *δ* 3.64 (6H, s, OMe), 3.87 (3H, s, OMe), 5.10 (2H, s, OCH_2_), 5.11 (2H, s, OCH_2_), 6.16 (1H, d, *J* = 2.2 Hz, H-5’), 6.23 (1H, d, *J* = 2.2 Hz, H-3’), 7.44 (12H, m, H-2, H-6, H-2’’’– H-6’’’, H-2’’– H-6’’), 7.68 (1H, d, *J* = 15.4 Hz, H-8), 7.79 (1H, d, *J* = 15.4 Hz, H-7); ^13^C NMR (CDCl_3_, 75 MHz) *δ* 56.0 (OMe), 60.9 (OMe), 70.3 (OCH_2_), 71.3 (OCH_2_), 92.9 (C-3’), 95.6 (C-5’), 105.7 (C-1’), 106.8 (C-2, C-6), 127.1 (C-8), 127.3 (C-2’’’, C-6’’’), 127.6 (C-2’’, C-6’’), 127.9 (C-4’’’), 128.2 (C-4’’), 128.3 (C-3’’’, C-5’’’), 128.7 (C-3’’, C-5’’), 130.7 (C-1), 135.6 (C-1’’’), 135.8 (C-1’’), 140.0 (C-4), 142.5 (C-7), 153.1 (C-3,C-5), 161.4 (C-2’), 165.2 (C-6’), 168.1 (C-4’), 192.5 (C-9).

#### Synthesis and analytical data of 2’,4’-dibenyloxy-6’-hydroxy-2-bromo-4-fluorochalcone (5i)


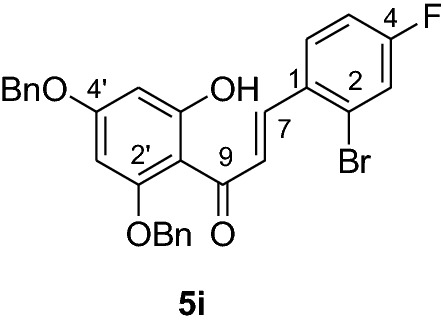


According to general procedure I, 2,4-dibenzyloxy-6-hydroxyacetophenone (**3**) (0.5 g, 1.4 mmol) was condensed with 2-bromo-4-chlorobenzaldehyde (**4i**) (0.32 g, 1.4 mmol) in the presence of aqueous KOH (60%, 1.5 mL) in ethanol (8.5 mL). The product **5i** was obtained in 77% yield. mp 132–133 °C; UV(MeOH) λ_max_: 271, 333 nm; IR *ν̃* [cm^−1^]: 1670 (conjugated C = O), 1603 (C = C), 1154, and 1025 (C-O);^1^H NMR (CDCl_3_, 300 MHz) *δ* 5.01 (2H, s, OCH_2_), 5.11 (2H, s, OCH_2_), 6.17 (1H, d, *J* = 2.2 Hz, H-5’), 6.22 (1H, d, *J* = 2.2 Hz, H-3’), 6.57 (1H, dd, *J* = 9.3 Hz and *J* = 3 Hz, H-3), 6.87 (1H, m, H-5), 7.44 (11H, m, H-6, H-2’’’– H-6’’’, H-2’’– H-6’’), 7.71 (1H, d, *J* = 15.4 Hz, H-8), 7.96 (1H, d, *J* = 15.4 Hz, H-7); ^13^C NMR (CDCl_3_, 75 MHz) *δ* 70.3 (OCH_2_), 71.6 (OCH_2_), 92.6 (C-3’), 95.0 (C-5’), 106.3 (C-1’), 113.9 (C-5), 114.2 (C-2), 117.8 (C-3), 119.9 (C-8), 127.6 (C-2’’’, C-6’’’), 128.7 (C-2’’, C-6’’), 128.8 (C-4’’’), 129.0 (C-4’’), 131.0 (C-3’’’, C-5’’’), 134.2 (C-3’’, C-5’’), 134.3 (C-6), 135.0 (C-1), 135.7 (C-1’’’), 136.7 (C-1’’), 139.3 (C-7), 159.9 (d, C-4), 161.6 (C-2’), 165.5 (C-6’), 168.5 (C-4’), 192.3 (C-9).

#### Synthesis of 2’,4’-dibenyloxy-6’-hydroxy-2,3-dichlorochalcone (5j)


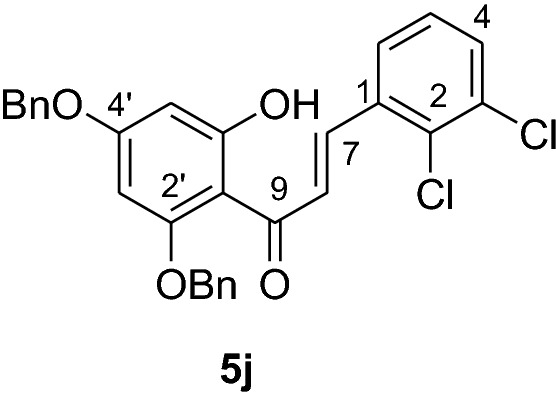


According to general procedure I, 2,4-dibenzyloxy-6-hydroxyacetophenone (**3**) (0.5 g, 1.4 mmol) was condensed with 2,3-dichlorobenzaldehyde (**4j**) (0.25 g, 1.4 mmol) in the presence of aqueous KOH (60%, 1.5 mL) in ethanol (8.5 mL). The product **5j** was obtained in 97% yield. mp 121–123 °C; *R*_f_ 0.60 (petroleum ether:EtOAc 4:1); UV(MeOH); λ_max_: 274, 328 nm; IR *ν̃* [cm^−1^]: 1605 (C = C), 1560 (C = C), 1337, 1204, 1154, and 1044 (C-O); ^1^H NMR (CDCl_3_, 300 MHz) *δ* 5.01 (2H, s, OCH_2_), 5.12 (2H, s, OCH_2_), 6.18 (1H, d, *J* = 2.2 Hz, H-5’), 6.23 (1H, d, *J* = 2.2 Hz, H-3’), 6.62 (1H, dd, *J* = 7.5 Hz and *J* = 2.4 Hz, H-6), 6.84 (1H, t, *J* = 8.1 Hz, H-5), 7.40 (11H, m, H-4, H-2’’’– H-6’’’, H-2’’– H-6’’), 7.75 (1H, d, *J* = 15.4 Hz), 8.06 (1H, d, *J* = 15.4 Hz); ^13^C NMR (CDCl_3_, 75 MHz) *δ* 70.3 (OCH_2_), 71.3 (OCH_2_), 92.5 (C-3’), 95.1 (C-5’), 106.3 (C-1’), 125.5 (C-8), 127.1, 127.6 (C-2’’’, C-6’’’), 128.3 (C-2’’, C-6’’), 128.4 (C-4’’’), 128.5 (C-4’’), 128.6 (C-2), 128.7 (C-5), 128.8 (3’’’, C-5’’’), 130.8 (C-3’’, C-5’’), 133.1 (C-4), 133.6 (C-3), 135.2 (C-1), 135.7 (C-1’’’), 135.8 (C-1’’), 137.6 (C-7), 161.6 (C-2’), 165.5 (C-6’), 168.7 (C-4’), 192.1 (C-9).

#### Synthesis of 2’,4’-dibenzyloxy-6’-hydroxy-4-cyanochalcone (5k)


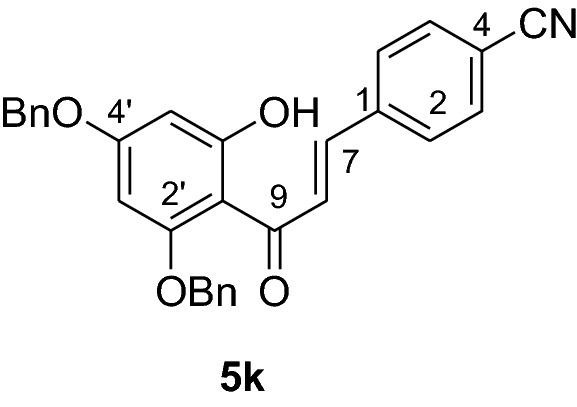


According to general procedure I, 2,4-dibenzyloxy-6-hydroxyacetophenone (**3**) (0.5 g, 1.4 mmol) was condensed with 4-cyanobenzaldehyde (**4 k**) (0.19 g, 1.4 mmol) in the presence of aqueous KOH (60%, 1.5 mL) in ethanol (8.5 mL). The product **5 k** was obtained in 67% yield. mp 148–150 °C; ^1^H NMR (CDCl_3_, 300 MHz) *δ* 5.03 (2H, s, OCH_2_), 5.11 (2H, s, OCH_2_), 6.18 (1H, d, *J* = 2.2 Hz, H-5’), 6.23 (1H, d, *J* = 2.2 Hz, H-3’), 7.02 (2H, d, *J* = 7.0 Hz, H-3, H-5), 7.43 (12H, m, H-2, H-6, H-2’’’– H-6’’’, H-2’’– H-6’’), 7.58 (1H, d, *J* = 15.4 Hz), 7.88 (1H, d, *J* = 15.4 Hz); ^13^C NMR (CDCl_3_, 75 MHz) *δ* 70.5 (OCH_2_), 71.4 (OCH_2_), 92.6 (C-3’), 95.2 (C-5’), 106.3 (C-1’), 112.4 (C-4), 118.6 (CN), 127.6 (C-8), 127.8 (C-2, C-6), 127.9 (C-2’’’, C-6’’’), 128.7 (C-2’’, C-6’’), 128.8 (C-4’’’), 129.0 (C-4’’), 129.1 (C-3’’’, C-5’’’), 130.9 (C-3’’, C-5’’), 132.3 (C-3, C-5), 135.2 (C-1), 135.7 (C-1’’’), 139.5 (C-1’’), 139.7 (C-7), 161.6 (C-2’), 165.8 (C-6’), 168.8 (C-4’), 192.3 (C-9).

#### Synthesis and analytical data of 2’,4’-dibenzyloxy-6’-hydroxy-2-chlorochalcone (5l)


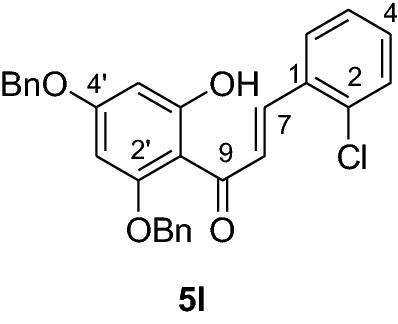


According to general procedure I, 2,4-dibenzyloxy-6-hydroxyacetophenone (**3**) (1 g, 2.8 mmol) was condensed with 2-chlorobenzaldehyde (**4 l**) (0.4 g, 2.8 mmol) in the presence of aqueous KOH (60%, 3 mL) in ethanol (15 mL). The product **5 l** was obtained in 77% yield. mp 138–139 °C; *R*_f_ 0.59 (petroleum ether:EtOAc 4:1); UV(MeOH) λ_max_: 270, 338 nm; IR *ν̃* [cm^−1^]: 1608 (C = C),1578 (C = C), 1554, 1425, 11, 1156, and 1040 (C-O); ^1^H NMR (CDCl_3_, 300 MHz) *δ* 5.04 (2H, s, OCH_2_), 5.10 (2H, s, OCH_2_), 6.17 (1H, d*, J* = 2.4 Hz, H-5’), 6.23 (1H, d, *J* = 2.4 Hz, H-3’), 6.75 (1H, dd*, J* = 7.8 and 2.4 Hz, H-3), 6.92 (1H, d, *J* = 8.7 Hz, H-5), 7.19 (2H, m, H-4, H-6), 7.41 (10H, m, H-2’’’– H-6’’’, H-2’’– H-6’’), 7.81 (1H, d*, J* = 15.6 Hz, H-8), 8.10 (1H, d*, J* = 15.6 Hz, H-7); ^13^C NMR (CDCl_3_, 75 MHz) *δ* 70.3 (OCH_2_), 71.4 (OCH_2_), 92.6 (C-3’), 95.0 (C-5’), 106. 3 (C-1’), 126.1 (C-8), 127.6 (C-5), 128.2 (C-2’’’, C-6’’’), 128.3 (C-2’’, C-6’’), 128.4 (C-4’’’), 128.7 (C-4’’), 128.8 (C-6), 128.9 (C-3), 129.6 (C-3’’’-C-5’’’), 129.8 (C-3’’-C-5’’), 128.85 (C-4), 134.5(C-2), 135.2 (C-1), 135.7 (C-1’’’), 137.1 (C-1’’), 140.7 (C-7), 161.6 (C-2’), 165.4 (C-6’), 168.6 (C-4’), 192.3 (C-9).

#### Synthesis of 2’,4’-dibenzyloxy-6’-hydroxy-3-chlorochalcone (5m)


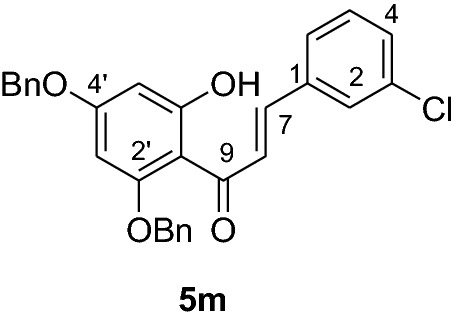


According to general procedure I, 2,4-dibenzyloxy-6-hydroxyacetophenone (**3**) (0.7 g, 2.0 mmol) was condensed with 3-chlorobenzaldehyde (**4 m**) (0.28 g, 2.0 mmol) in the presence of aqueous KOH (60%, 3 mL) in ethanol (15 mL). The product **5 m** was obtained in 92% yield. mp 135–136 °C; *R*_f_ 0.60 (petroleum ether:EtOAc 4:1); UV(MeOH) λ_max_: 280, 334 nm; IR *ν̃* [cm^−1^]: 1607 (C = C), 1573 (C = C), 1269, 1226, 1116, and 1024 (C-O); ^1^H NMR (CDCl_3_, 300 MHz) *δ* 5.0 (2H, s, OCH_2_), 5.1 (2H, s, OCH_2_), 6.18 (1H, d, *J* = 2.4 Hz, H-5’), 6.22 (1H, d, *J* = 2.4 Hz, H-3’), 6.89 (1H, d, *J* = 7.8 Hz, H-4), 7.10 (1H, t, *J* = 7.8 Hz, H-5), 7.2 (1H, br s, H-2), 7.25 (1H, dd, *J* = 7.8, *J* = 3.6 Hz, H-6), 7.45 (10H, m, H-2’’’– H-6’’’, H-2’’– H-6’’), 7.62 (1H, d, *J* = 15.9, H-8), 7.83 (1H, d, *J* = 15.9, H-7), 14.3 (1H, s, 6’OH); ^13^C NMR (CDCl_3_, 75 MHz) *δ* 70.3 (OCH_2_), 71.4 (OCH_2_), 92.6 (C-3’), 95.0 (C-5’), 106.3 (C-1’), 126.1, 127.6 (C-6), 128.2 (C-2), 128.3 (C-2’’’-C-6’’’), 128.4 (C-2’’-C-6’’), 128.7 (C-4’’’), 128.8 (C-4’’), 128.85 (C-4), 128.9 (C-3’’’, C-5’’’), 129.6 (C-3’’, C-5’’), 129.8 (C-5), 134.5 (C-3), 135.2 (C-1), 135.7 (C-1’’’), 137.1 (C-1’’), 140.7 (C-7), 161.6 (C-2’), 165.4 (C-6’), 168.6 (C-4’), 192.3 (C-9).

#### Synthesis and analytical data of 2’,4’-dibenzyloxy-6’-hydroxy-2,4-dichlorochalcone (5n)


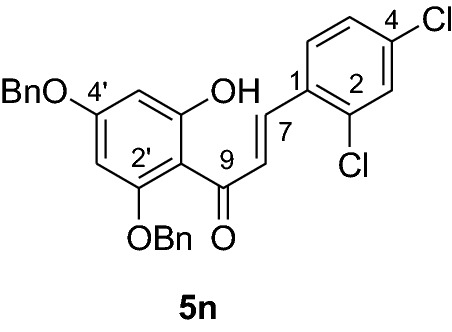


According to general procedure I, 2,4-dibenzyloxy-6-hydroxyacetophenone (**3**) (0.5 g, 1.4 mmol) was condensed with 2,4-dichlorobenzaldehyde (**4n**) (0.25 g, 1.4 mmol) in the presence of aqueous KOH (60%, 3 mL) in ethanol (15 mL). The product **5n** was obtained in 97% yield. mp 127–129 °C; *R*_f_ 0.65 (petroleum ether:EtOAc 4:1); UV(MeOH) λ_max_: 281, 330 nm; IR *ν̃* [cm^−1^]: 1623 (C = C), 1559 (C = C), 1464, 1299, 1101, and 1049 (C-O); ^1^H NMR (CDCl_3_, 300 MHz) *δ* 5.03 (2H, s, OCH_2_), 5.10 (2H, s, OCH_2_), 6.17 (1H, dd, *J* = 2.1 Hz, H-5’), 6.22 (1H, dd, *J* = 2.1 Hz, H-3’), 6.59 (1H, d*, J* = 8.4 Hz, H-6), 6.85 (1H, dd, *J* = 8.7, *J* = 2.4 Hz, H-5), 7.32 (1H, d, *J* = 2.4 Hz, H-3), 7.45 (10H, m, H-2’’’– H-6’’’, H-2’’–H-6’’), 7.75 (1H, d*, J* = 15.6 Hz, H-8), 7.99 (1H, d*, J* = 15.6 Hz, H-7), 14.3 (1H, s, 6’OH); ^13^C NMR (CDCl_3_, 75 MHz) *δ* 70.4 (OCH_2_), 71.5 (OCH_2_), 92.7 (C-3’), 95.0 (C-5’), 106.4 (C-1’), 126.3 (C-8), 127.6 (C-5), 128.0 (C-2’’’,C-6’’’), 128.2 (C-2’’,C-6’’), 128.4 (C-4’’’), 128.6 (C-4’’), 128.7 (C-3’’’,C-5’’’), 128.9 (C-3’’, C-5’’), 129.0 (C-6), 129.4 (C-3), 130.1 (C-1), 135.0 (C-2), 135.2 (C-4), 135.7 (C-1’’’), 138.2 (C-1’’), 139.0 (C-7), 161.6 (C-2’), 165.6 (C-6’), 168.5 (C-4’), 192.0 (C-9).

#### Synthesis and analytical data of 2’,4’-dibenzyloxy-6’-hydroxy-3,5-dichlorochalcone (5o)


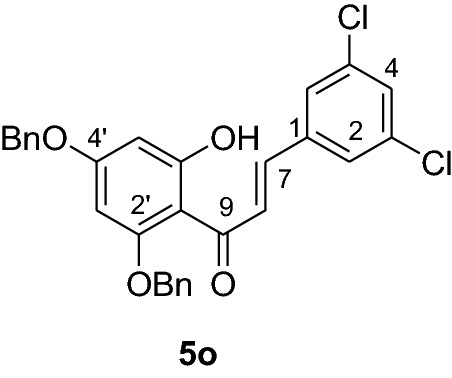


According to general procedure I, 2,4-dibenzyloxy-6-hydroxyacetophenone (**3**) (1 g, 2.8 mmol) was condensed with 3,5-dichlorobenzaldehyde (**4o**) (0.54 g, 2.8 mmol) in the presence of aqueous KOH (60%, 3 mL) in ethanol (15 mL). The product **5o** was obtained in 70% yield. mp: 125–127 °C; *R*_f_ 0.66 (petroleum ether:EtOAc 4:1); UV(MeOH) λ_max_: 278, 329 nm; IR *ν̃* [cm^−1^]: 1620 (C = C), 1574 (C = C), 1552, 1366, 1201, 1156, and 1045 (C-O); ^1^H NMR (CDCl_3_, 300 MHz) *δ* 5.05 (2H, s, OCH_2_), 5.10 (2H, s, OCH_2_), 6.18 (1H, d*, J* = 3.3 Hz, H-5’), 6.22 (1H, d*, J* = 2.4 Hz, H-3’), 7.05 (2H, br s, H-2, H-6), 7.28 (1H, m, H-4), 7.42 (10H, m, H-2’’’– H-6’’’, H-2’’– H-6’’), 7.50 (1H, d*, J* = 15.9 Hz, H-8), 7.75 (1H, d*, J* = 15.6 Hz, H-7); ^13^C NMR (CDCl_3_, 75 MHz) *δ* 70.4 (OCH_2_), 71.5 (OCH_2_), 92.7 (C-3’), 95.0 (C-5’), 106.4 (C-1’), 126.4 (C-8), 127.6 (C-2, C-6), 127.6 (C-2’’’, C-6’’’), 128.0 (C-2’’, C-6’’), 128.4 (C-4’’’), 128.7 (C-4’’), 128.9 (C-3’’’, C-5’’’), 129.0 (C-3’’, C-5’’), 130.1 (C-4), 135.0 (C-3, C-5), 135.2 (C-1), 135.7 (C-1’’’), 138.2 (C-1’’), 139.0 (C-7), 161.6 (C-2’), 165.6 (C-6’), 168.5 (C-4’), 192.0 (C-9).

#### Synthesis and analytical data of 2’,4’-dibenzyloxy-6’-hydroxy-2,6-dichlorochalcone (5p)


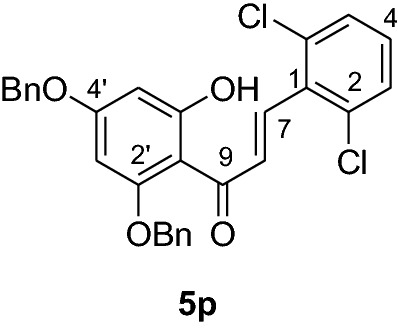


According to general procedure I, 2,4-dibenzyloxy-6-hydroxyacetophenone (**3**) (1 g, 2.8 mmol) was condensed with 2,6-dichlorobenzaldehyde (**4p**) (0.54 g, 2.8 mmol) in the presence of aqueous KOH (60%, 3 mL) in ethanol (15 mL). The product **5p** was obtained in 69% yield. mp 121–122 °C; *R*_f_ 0.59 (petroleum ether:EtOAc 4:1); UV(MeOH) λ_max_: 276, 331 nm; IR *ν̃* [cm^−1^]: 1618 (C = C), 1577(C = C), 1497, 1453, 1284, 1174, and 1026 (C-O); ^1^H NMR (CDCl_3_, 300 MHz) *δ* 5.01 (2H, s, OCH_2_), 5.07 (2H, s, OCH_2_), 6.09 (1H, d*, J* = 2.1 Hz, H-5’), 6.21 (1H, d*, J* = 2.1 Hz, H-3’), 7.14 (3H, m, H-3, H-4, H-5), 7.28 (10H, m, H-2’’’– H-6’’’, H-2’’– H-6’’), 7.71 (1H, d*, J* = 16.2 Hz, H-8), 7.94 (1H, d*, J* = 16.2, H-7), 14.0 (1H, s); ^13^C NMR (CDCl_3_, 75 MHz) *δ* 70.3 (OCH_2_), 71.1 (OCH_2_), 92.8 (C-3’), 94.9 (C-5’), 106.6 (C-1’), 127.5 (C-8), 127.6 (C-3, C-5), 128.0 (C-2’’’, C-6’’’), 128.3 (C-2’’, C-6’’), 128.4 (C-4’’’), 128.5 (C-4’’), 128.7 (C-3’’’, C-5’’’), 129.2 (C-3’’, C-5’’), 133.0 (C-4), 134.8 (C-2, C-6), 135.0 (C-1), 135.3 (C-1’’’), 135.7 (C-1’’), 136.1 (C-7), 161.6 (C-2’), 165.5 (C-6’), 168.2 (C-4’), 192.5 (C-9).

#### Synthesis and analytical data of 2’,4’-dibenzyloxy-6’-hydroxy-2,5-dichlorochalcone (5q)


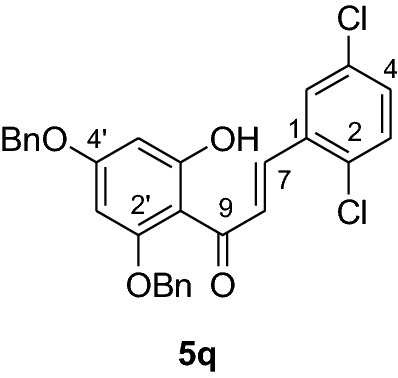


According to general procedure I, 2,4-dibenzyloxy-6-hydroxyacetophenone (**3**) (0.7 g, 2.0 mmol) was condensed with 2,5-dichlorobenzaldehyde (**4q**) (0.37 g, 2.0 mmol) in the presence of aqueous KOH (60%, 3 mL) in ethanol (15 mL). The product **5q** was obtained in 96% yield. mp 128–129 °C; *R*_f_ 0.63 (petroleum ether:EtOAc 4:1); UV(MeOH) λ_max_: 275, 338 nm; IR *ν̃* [cm^−1^]: 1617 (C = C), 1574 (C = C), 1552, 1423, 1201, 1165, and 1045 (C-O); ^1^H NMR (CDCl_3_, 300 MHz) *δ* 5.06 (2H, s, OCH_2_), 5.10 (2H, s, OCH_2_), 6.17 (1H, d*, J* = 2.4 Hz, H-5’), 6.22 (1H, d*, J* = 2.4 Hz, H-3’), 7.08 (1H, d, *J* = 2.4 Hz, H-6), 7.20 (1H, dd*, J* = 8.1, *J* = 2.4 Hz, H-4), 7.29 (1H, m, H-3), 7.30–7.41 (10H, m, H-2’’’– H-6’’’, H-2’’– H-6’’), 7.73 (1H, d*, J* = 15.9 Hz, H-8), 7.98 (1H, d*, J* = 15.9 Hz, H-7), 14.1 (1H, s, 6’OH); ^13^C NMR (CDCl_3_, 75 MHz) *δ* 70.3 (OCH_2_), 71.4 (OCH_2_), 92.7 (C-3’), 95.0 (C-5’), 106.4 (C-1’), 127.0 (C-8), 127.6 (C-2’’’, C-6’’’), 127.7 (C-2’’, C-6’’), 127.9 (C-4’’’), 128.3 (C-4’’), 128.7 (C-6), 128.8 (C-3’’’, C-5’’’), 130.3 (C-3’’, C-5’’), 130.9 (C-2), 131.1 (C-4), 132.7 (C-3), 133.4 (C-5), 134.9 (C-1), 135.0 (C-1’’’), 135.7 (C-1’’), 136.2 (C-7), 161.6 (C-2’), 165.6 (C-6’), 168.4 (C-4’), 192.1 (C-9).

#### Synthesis and analytical data of 2’,4’-dibenzyloxy-6’-hydroxy-3,4-dichlorochalcone (5r)


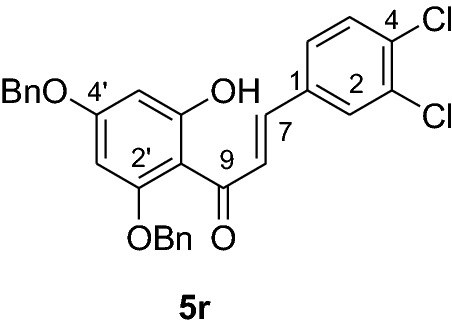


According to general procedure I, 2,4-dibenzyloxy-6-hydroxyacetophenone (**3**) (0.7 g, 2.0 mmol) was condensed with 3,4-dichlorobenzaldehyde (**4r**) (0.37 g, 2.0 mmol) in the presence of aqueous KOH (60%, 3 mL) in ethanol (15 mL). The product **5r** was obtained in 98% yield. mp 130–131 °C; *R*_f_ 0.64 (petroleum ether:EtOAc 4:1); UV(MeOH) λ_max_: 276, 329 nm; IR *ν̃* [cm^−1^]: 1618 (C = C), 1578 (C = C), 1420, 1202, 1160, and 1022(C-O); ^1^H NMR (CDCl_3_, 300 MHz) *δ* 5.05 (2H, s, OCH_2_), 5.11 (2H, s, OCH_2_), 6.18 (1H, d*, J* = 2.4 Hz, H-5’), 6.22 (1H, d, *J* = 2.4 Hz, H-3’), 6.74 (1H, dd*, J* = 8.4, *J* = 1.8 Hz, H-6), 7.20 (1H, d*, J* = 8.1 Hz, H-5), 7.28 (1H, d*, J* = 2.4 Hz, H-2), 7.43 (10H, m, H-2’’’–H-6’’’, H-2’’– H-6’’), 7.54 (1H, d*, J* = 15.6 Hz, H-8), 7.79 (1H, d*, J* = 15.6 Hz, H-7); ^13^C NMR (CDCl_3_, 75 MHz) *δ* 70.2 (OCH_2_), 70.6 (OCH_2_), 92.6 (C-3’), 95.0 (C-5’), 106.5 (C-1’), 126.7 (C-8), 127.6 (C-6), 127.7 (C-2’’’, C-6’’’), 127.9 (C-2’’, C-6’’), 128.4 (C-4’’’), 128.5 (C-4’’), 128.8 (C-2), 128.9 (C-3’’’, C-5’’’), 129.2 (C-3’’, C-5’’), 130.1 (C-5), 130.5 (C-4), 132.7 (C-3), 135.2 (C-1), 135.3 (C-1’’’), 135.7 (C-1’’), 139.4 (C-7), 161.9 (C-2’), 165.9 (C-6’), 192.8 (C-9).

### Synthesis of the dibenzylated flavanones 6a-r

#### General procedure II for synthesis of the dibenzylated flavanones 6a-r [16, 30]

A solution of a chalcone **5** (1 mmol) in ethanol was treated with sodium acetate (8 mmol) and the solution was refluxed for 48 h, cooled to room temperature and then diluted with water (60 mL/mmol). After extraction with CH_2_Cl_2_ (3 × 30 mL/mmol), the combined organic extracts were dried over anhydrous MgSO_4_, filtered and concentrated *in vacuo*. Purification was achieved by column chromatography over silica gel using petroleum ether:EtOAc (4:1) as eluent to furnish the product **6** as a white solid.

#### Synthesis and analytical data of 5,7-dibenzyloxyflavanone (6a)


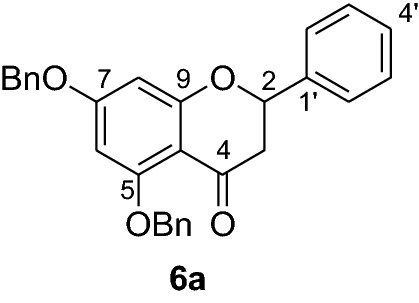


According to the general procedure II, 2′4’-dibenzyloxy-6’-hydroxychalcone (**5a**) (700 mg, 1.6 mmol) was dissolved in ethanol (75 mL). Sodium acetate (1.05 g, 12.8 mmol) was added and the solution was refluxed for 48 h. After workup, the product **6a** was obtained as a white solid in 50% yield. mp 119–120 °C; *R*_f_ 0.70 (petroleum ether:EtOAc 4:1); UV(MeOH) λ_max_: 270, 330 nm; IR *ν̃* [cm^−1^]: 1674 (conjugated C = O), 1599 (C = C), 1551(C = C), 1062, and 1028 (C-O); ^1^H NMR (CDCl_3_, 300 MHz) *δ* 2.82 (1H, dd, *J* = 2.7, *J* = 16.5 Hz, H-3), 3.06 (1H, dd, *J* = 13.2, *J* = 16.5 Hz, H-3), 5.0 (2H, s, OCH_2_), 5.2 (2H, s, OCH_2_), 5.43 (1H, dd, *J* = 2.7, *J* = 13.2 Hz, H-2), 6.25 (1H, s, H-8), 6.26 (1H, s, H-6), 7.59–7.30 (15H, m, H-2’–H-6’, H-2’’–H-6’’, H-2’’’–H-6’’’); ^13^C NMR (CDCl_3_, 75 MHz) *δ* 45.7 (C-3), 70.2 (OCH_2_), 70.3 (OCH_2_), 79.3 (C-2), 94.7 (C-6), 95.1 (C-8), 106.4 (C-10), 126.1 (C-2’,C-6’), 126.5 (C-2’’’, C-6’’’), 127.5 (C-2’’, C-6’’), 127.6 (C-4’), 128.3 (C-4’’’), 128.5 (C-4’’), 128.6 (C-3’,C-5’), 128.7 (C-3’’’, C-5’’’), 128.8 (C-3’’, C-5’’), 135.7 (C-1’), 136.3 (C-1’’’), 138.7 (C-1’’), 161.0 (C-9), 164.8 (C-5), 164.9 (C-7), 188.8 (C-4); MS (EI, 70 eV) *m/z* (%) 436 (19) [M]^+^, 346 (7), 317 (4), 240 (3), 91 (100).

#### Synthesis and analytical data of 5,7-dibenzyloxy-4’-fluoroflavanone (6b)


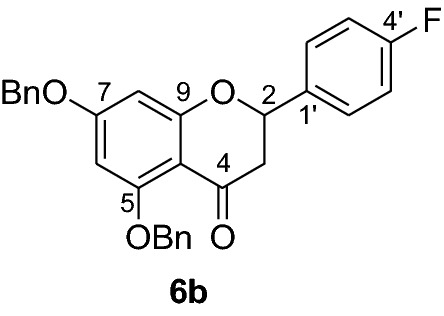


According to the general procedure II, 2′4’-dibenzyloxydibenzyloxy-6’-hydroxy-4-fluorochalcone (**5b**) (250 mg, 0.5 mmol) was dissolved in ethanol (60 mL). Sodium acetate (0.33 g, 4 mmol) was added and the solution was refluxed for 48 h. After workup, the product **6b** was obtained in 48% yield. mp 76–78 °C; UV(MeOH) λ_max_: 272, 335 nm; ^1^H NMR (CDCl_3_, 300 MHz) *δ* 2.69 (1H, dd, *J* = 2.7, *J* = 16.2 Hz, H-3), 3.00 (1H, dd, *J* = 12.6, *J* = 16.2 Hz, H-3), 5.20 (2H, s, OCH_2_), 5.22 (2H, s, OCH_2_), 5.56 (1H, dd, *J* = 2.7, *J* = 12.6 Hz, H-2), 6.32 (1H, d, *J* = 2.4 Hz, H-8), 6.42 (1H, d, *J* = 2.4 Hz, H-6), 7.69–7.18 (14H, m, H-2’,H-6’, H-3’, H-5’, H-2’’’–H-6’’’, H-2’’–H-6’’);^13^C NMR (CDCl_3_, 75 MHz) *δ* 46.2 (C-3), 70.7 (OCH_2_), 70.8 (OCH_2_), 79.2 (C-2), 95.6 (C-6), 95.7 (C-8), 107.1 (C-10), 115.9 (C-3’, C-5’), 116.2 (C-2’, C-6’), 127.4 (C-2’’’, C-6’’’), 128.1 (C-2’’, C-6’’), 128.5 (C-4’’’), 128.9 (C-4’’), 129.0 (C-3’’’, C-5’’’), 129.3 (C-3’’, C-5’’), 129.4 (C-1’), 137.4 (C-1’’’), 138.8 (C-1’’), 161.8 (d, C-4’), 165.0 (C-9), 165.4 (C-5), 165.6 (C-7), 187.8 (C-4).

#### Synthesis and analytical data of 5,7-dibenzyloxy-3′4’-dimethoxyflavanone (6c)


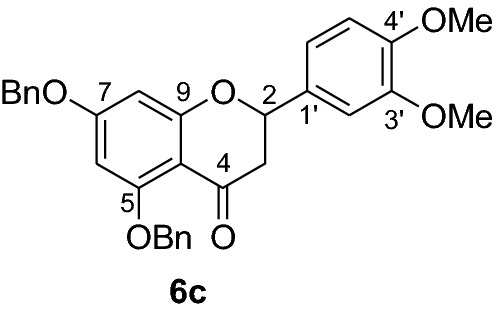


According to the general procedure II, 2’,4’-dibenyloxy-6’-hydroxy-3,4-dimethoxychalcone (**5c**) (200 mg, 0.4 mmol) was dissolved in ethanol (70 mL). Sodium acetate (0.26 g, 3.2 mmol) was added and the solution was refluxed for 48 h. The product **6c** was obtained in 40% yield. mp 91–92 °C; UV(MeOH) λ_max_: 276, 345 nm; IR *ν̃* [cm^−1^]: 1672 (conjugated C = O), 1595 (C = C), 1571 (C = C), 1257, 1117, 1075, and 1012 (C-O); ^1^H NMR(CDCl_3_, 300 MHz) *δ* 2.77 (1H, dd, *J* = 2.7, *J* = 16.5 Hz, H-3), 3.08 (1H, dd, *J* = 13.2 Hz, *J* = 16.5 H-3)*,* 3.90 (1H, s, OMe), 3.92 (1H, s, OMe), 5.04 (2H, s, OCH_2_), 5.10 (2H, s, OCH_2_), 5.38 (1H, dd, 2.7, *J* = 16.5 Hz, H-2), 6.24 (2H, br s, H-8), 6.90 (1H, br s, H-6), 7.0 (2H, m, H-2’, H-5’), 7.18 (1H, m, H-6’), 7.40–7.58 (10H, m, H-2’’’–H-6’’’, H-2’’–H-6’’);^13^C NMR (CDCl_3_, 75 MHz) *δ* 45.6 (C-3), 55.8 (OMe), 55.9 (OMe), 70.2 (OCH_2_), 70.4 (OCH_2_), 79.3 (C-2), 94.7 (C-6), 95.1 (C-8), 106.6 (C-10), 109.4 (C-2’), 118.8 (C-5’), 126.5 (C-6’), 127.2 (C-2’’’, C-6’’’), 127.6 (C-2’’, C-6’’), 128.0 (C-4’’’), 128.3 (C-4’’), 128.6 (C-3’’’, C-5’’’), 128.7 (C-3’’, C-5’’), 131.1(C-1’), 135.7 (C-1’’’), 136.3 (C-1’’), 149.2 (C-4’), 149.4 (C-3’), 158.2 (C-9), 161.0 (C-5), 164.9 (C-7), 189.0 (C-4); MS (EI, 70 eV) *m/z* (%) 496 (16) [M]^+^, 406 (24), 348 (9), 227 (13), 151 (27), 91 (100); HRMS (EI, M^+^) calculated for C_31_H_28_O_6_ 496.1886 found 496.1880.

#### Synthesis and analytical data of 5,7-dibenzyloxy-2′6’-dimethoxyflavanone (6d)


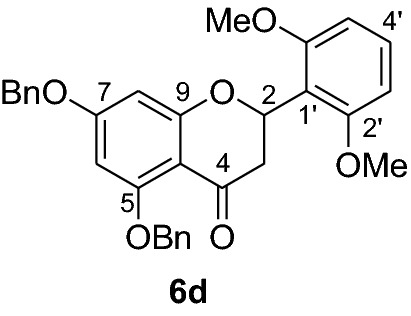


According to the general procedure II, 2’,4’-dibenyloxy-6’-hydroxy-2,6-dimethoxychalcone (**5d**) (250 mg, 0.5 mmol) was dissolved in ethanol (75 mL). Sodium acetate (0.32 g, 4 mmol) was added and the solution was refluxed for 48 h. The product **6d** was obtained in 52%. mp 87 °C; UV(MeOH) λ_max_: 275, 344 nm; IR *ν̃* [cm^−1^]: 1667 (conjugated C = O), 1607 (C = C), 1572 (C = C), 1051, and 1025 (C-O); ^1^H NMR (CDCl_3_, 300 MHz) *δ* 2.88–2.80 (2H, m, H-3), 3.7 (3H, s, OMe), 3.8 (3H, s, OMe), 5.0 (2H, s, OCH_2_), 5.1 (2H, s, OCH_2_), 5.77 (1H, dd, *J* = 4.8, *J* = 11.1 Hz, H-2), 6.24 (1H, d, *J* = 2.4 Hz, H-8), 6.28 (1H, d, *J* = 2.4 Hz, H-6), 6.83 (1H, br d, H-4), 7. 20–7.29 (10H, m, H-2’’’–H-6’’’, H-2’’–H-6’’), 7.60 (2H, br d, *J* = 7.5 Hz, H-3’, H-5’); ^13^C NMR (CDCl_3_, 75 MHz) *δ* 44.8 (C-3), 55.8 (OMe), 55.9 (OMe), 70.2 (OCH_2_), 70.3 (OCH_2_), 74.3 (C-2), 94.7 (C-6), 95.0 (C-8), 106.5 (C-10), 111.5 (C-1’), 112.5 (C-3’), 113.5 (C-5’), 126.5 (C-2’’’, C-6’’’), 127.5 (C-2’’, C-6’’), 128.3 (C-4’’), 128.5 (C-4’’), 128.6 (C-3’’’, C-5’’’), 128.7 (C-3’’, C-5’’), 128.9 (C-4’), 135.8 (C-4’’’), 136.6 (C-4’’), 149.9 (C-2’), 153.5 (C-6’), 161.1 (C-5), 164.1 (C-9), 165.2 (C-7), 189.5 (C-4); MS (EI, 70 eV) *m/z* (%) 496 (24) [M]^+^, 465 (14), 405 (29), 348 (9), 91 (100); HRMS (EI, M^+^) calculated for C_31_H_28_O_6_ 496.1886 found 496.1870.

#### Synthesis and analytical data of 5,7-dibenzyloxy-4’-chloroflavanone (6e)


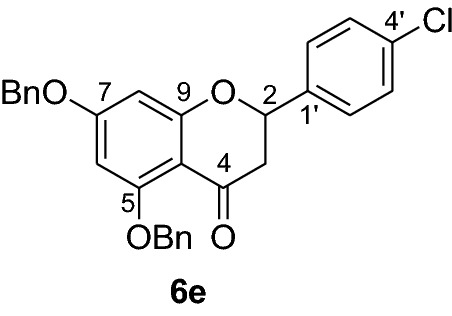


According to the general procedure II, 2’,4’-dibenzyloxy-6’-hydroxy-4-chlorochalcone (**5e**) (300 mg, 0.6 mmol) was dissolved in ethanol (60 mL). Sodium acetate (0.39 g, 4.8 mmol) was added and the solution was refluxed for 48 h. The product **6e** was obtained in 40% yield. mp 129–131 °C; *R*_f_ 0.56 (petroleum ether:EtOAc 4:1); UV(MeOH) λ_max_: 271, 336 nm; IR *ν̃* [cm^−1^]:1671 (conjugate C = O), 1606 (C = C), 1571(C = C), 1064, and 1035 (C-O); ^1^H NMR (CD_3_COCD_3_, 300 MHz) *δ* 2.75 (1H, dd, *J* = 3.0 Hz, *J* = 16.2 Hz, H-3), 3.00 (1H, dd, *J* = 12.6 Hz, *J* = 16.2 Hz, H-3), 5.20 (1H, s, OCH_2_), 5.21 (1H, s, OCH_2_), 5.57 (1H, dd, *J* = 3 Hz, *J* = 12.6 Hz, H-2), 6.23 (1H, d, *J* = 2.2 Hz, H-8), 6.43 (1H, d, *J* = 2.2 Hz, H-6), 7.40 (10H, m, H-2’’’–H-6’’’, H-2’’–H-6’’), 7.59 (2H, d, *J* = 7.0, H-3’,H-5’), 7.6 (2H, d, *J* = 7.0 Hz, H-2’, H-6’); ^13^C NMR (CD_3_COCD_3_, 75 MHz) *δ* 46.1 (C-3), 70.7 (OCH_2_), 70.8 (OCH_2_), 79.1 (C-2), 95.7 (C-6), 95.8 (C-8), 107.0 (C-10), 127.4 (C-2’’’, C-6’’’), 128.1 (C-2’’, C-6’’), 128.5 (C-4’’’), 128.9 (C-4’’), 129.0, (C-3’’’, C-5’’’) 129.1 (C-3’’, C-5’’), 129.3 (C-2’, C-6’), 129.4 (C-3’, C-5’), 134.4 (C-4’), 137.4 (C-1’’’), 138.0 (C-1’’), 139.3 (C-1’), 161.9 (C-9), 165.3 (C-5), 165.5 (C-7), 188.8 (C-4); MS (EI,70 eV) *m/z* (%) 470 (11) [M]^+^, 379 (8), 348 (11), 257 (6), 180 (4), 91 (100); HRMS (EI, M^+^) calculated for C_29_H_23_O_4_Cl 470.1285 found 470.1260.

#### Synthesis and analytical data of 5,7-dibenzyloxy-2’-bromoflavanone (6f)


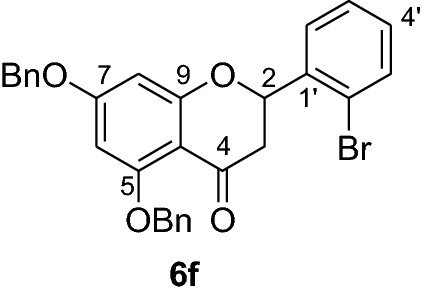


According to the general procedure II, 2’,4’-dibenyloxy-6’-hydroxy-2-bromochalcone (**5f**) (500 mg, 0.97 mmol) was dissolved in ethanol (75 mL). Sodium acetate (0.64 g, 7.76 mmol) was added and the solution was refluxed for 48 h. The product **6f** was obtained in 36% yield. mp 89–91 °C; UV(MeOH) λ_max_: 273, 338 nm; IR *ν̃* [cm^−1^]: 1672 (conjugated C = O), 1603 (C = C), 1569 (C = C), 1068, and 1024 (C-O); ^1^H NMR (CDCl_3_, 300 MHz) *δ* 2.82 (1H, dd, *J* = 13.2 Hz, *J* = 16.5 Hz, H-3), 2.98 (1H, dd, *J* = 16.2 Hz, *J* = 2.7 Hz, H-3), 5.06 (2H, s, OCH_2_), 5.17 (2H, s, OCH_2_), 5.8 (1H, dd, *J* = 10.5 Hz, *J* = 2.4 Hz, H-2), 6.27 (2H, br s, H-6, H-8), 7.20–7.70 (14H, m, H-3’–H-6’, H-2’’’–H-6’’’, H-2’’–H-6’’); ^13^C NMR (CDCl_3_, 75 MHz) *δ* 44.6 (C-3), 70.3 (OCH_2_), 70.4 (OCH_2_), 78.4 (C-2), 94.7 (C-6), 95.3 (C-8), 106.4 (C-10), 121.5 (C-2), 126.5 (C-2’’’, C-6’’’), 127.3 (C-2’’, C-6’’), 127.5 (C-4’’’), 127.6 (C-4’’), 127.9 (C-5’), 128.3 (C-3’’’, C-5’’’), 128.5 (C-3’’, C-5’’), 128.7 (C-6’), 129.8 (C-4’), 132.9 (C-3’), 135.7 (C-1’’’), 136.3 (C-1’’), 138.3 (C-1’’), 161.3 (C-9), 164.9 (C-5), 165.0 (C-7), 188.4 (C-4); MS (EI, 70 eV) *m/z* (%) 516 (16) [M + 2]^+^, 514 [M]^+^ (18), 435 (12), 425 (14), 423 (12), 256 (4), 91 (100); HRMS (EI, M^+^) calculated for C_29_H_23_O_4_Br 514.0780 found 514.0777.

#### Synthesis of 5,7-dibenyloxy-4-(trifluoromethyl)flavanone (6g)


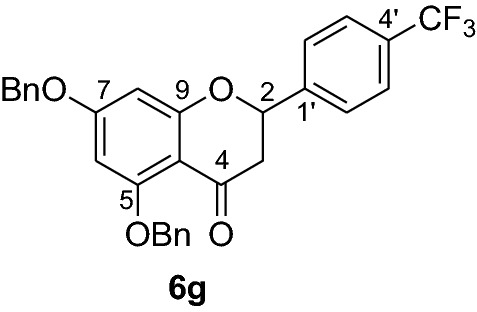


According to the general procedure II, 2’,4’-dibenyloxy-6’-hydroxy-4-(trifluoromethyl)chalcone (**5g**) (500 mg, 0.97 mmol) was dissolved in ethanol (75 mL). Sodium acetate (0.64 g, 7.8 mmol) was added and the solution was refluxed for 48 h. The product **6g** was obtained in 8% yield. ^1^H NMR (CD_3_COCD_3_, 300 MHz) *δ* 2.78 (1H, dd, *J* = 12.5 Hz, *J* = 3.3 Hz, H-3), 3.00 (1H, dd, *J* = 12.5 Hz, *J* = 13.3 Hz, H-3), 5.20 (2H, s, OCH_2_), 5.23 (2H, s, OCH_2_), 5.68 (1H, dd, *J* = 12.5 Hz, *J* = 2.5 Hz, H-2), 6.36 (1H, d, *J* = 2.2 Hz, H-8), 6.44 (1H, d, *J* = 2.2 Hz, H-6), 7.31–7.77 (14H, m, H-2’, H-3’, H-5’, H-6’, H-2’’’–H-6’’’, H-2’’–H-6’’); ^13^C NMR (CD_3_COCD_3_, 75 MHz) *δ* 45.7 (C-3), 70.2 (OCH_2_), 70.9 (OCH_2_), 78.7 (C-2), 95.7 (C-6), 95.6, 107.2 (C-10), 126.3 (CF_3_), 127.5 (C-3’, C-5’), 127.7 (C-2’’’, C-6’’’), 128.2 (C-2’’, C-6’’), 128.5 (C-4’’’), 128.9 (C-4’’), 129.0 (C-2’, C-6’), 129.3 (C-3’’’, C-5’’’), 129.4 (C-3’’, C-5’’), 132.0 (C-4’), 137.4 (C-1’’’), 138.0 (C-1’’), 144.8 (C-1’), 161.9 (C-9), 165.2 (C-5), 165.8 (C-7), 187.0 (C-4).

#### Synthesis and analytical data of 5,7-dibenzyloxy-3′4’5’-trimethoxyflavanone (6h)


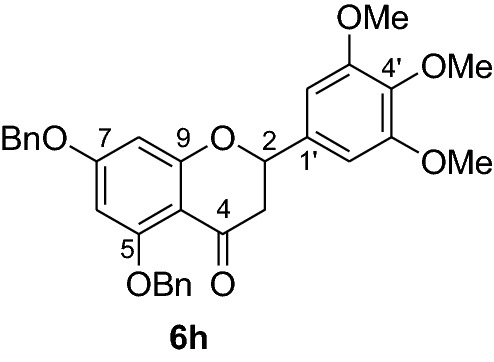


According to the general procedure II, 2’,4’-dibenyloxy-6’-hydroxy-3,4,5-trimethoxychalcone (**5h**) (400 mg, 0.76 mmol) was dissolved in ethanol (70 mL). Sodium acetate (0.5 g, 6.08 mmol) was added and the solution was refluxed for 48 h. The product **6h** was obtained in 38% yield. mp 84–86 °C; UV(MeOH) λ_max_: 274, 351 nm; IR *ν̃* [cm^−1^]: 1671 (conjugated C = O), 1607 (C = C), 1569, 1116, and 1032 (C-O); ^1^H NMR (CD_3_COCD_3_, 300 MHz) *δ* 2.68 (1H, dd, *J* = 16.5 Hz, *J* = 3 Hz, H-3), 3.06 (1H, dd, *J* = 12.9 Hz, *J* = 16.5 Hz, H-3), 3.75 (3H, s, OMe), 3.85 (6H, s, OMe), 5.19 (2H, s, OCH_2_), 5.22 (2H, s, OCH_2_), 5.44 (1H, dd, *J* = 12.9, *J* = 3 Hz, H-2), 6.32 (1H, d, *J* = 2.2 Hz, H-8), 6.41 (1H, d, *J* = 2.2 Hz), 7.28–7.66 (12H, m, H-2’, H-6’, H-2’’’–H-6’’’, H-2’’–H-6’’); ^13^C NMR (CD_3_COCD_3_, 75 MHz) *δ* 46.3 (C-3), 56.3 (OMe), 60.5 (OMe), 70.3 (OCH_2_), 70.8 (OCH_2_), 80.3 (C-2), 95.7 (C-6), 95.8 (C-8), 104.8 (C-2’, C-6’), 107.4 (C-10), 127.4 (C-2’’’, C-6’’’), 128.2 (C-2’’, C-6’’), 128.5 (C-4’’’), 128.9 (C-4’’), 129.0 (C-3’’’, C-5’’’), 129.3 (C-3’’, C-5’’), 135.7 (C-1’), 137.5 (C-4’), 138.1 (C-1’’’), 139.2 (C-1’’), 154.4 (C-3’, C-5’), 161.6 (C-9), 165.5 (C-5), 165.6 (C-7), 188.7 (C-4); MS (EI, 70 eV) *m/z* (%) 526 (40) [M]^+^, 435 (28), 407(16), 257 (16), 181 (26), 91 (100).

#### Synthesis and analytical data of 5,7-dibenzyloxy-2’-bromo-4’-fluoroflavanone (6i)


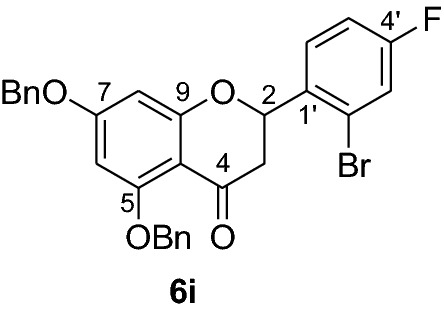


According to the general procedure II, 2’,4’-dibenyloxy-6’-hydroxy-2-bromo-4-fluorochalcone (**5i**) (400 mg, 0.75 mmol) was dissolved in ethanol (70 mL). Sodium acetate (0.49 g, 6.0 mmol) was added and the solution was refluxed for 48 h. The product **6i** was obtained as 48% yield. IR *ν̃* [cm^−1^]: 1668 (conjugated C = O), 1603 (C = C), 1154, and 1025 (C-O); ^1^H NMR (CDCl_3_, 300 MHz) *δ* 2.88 (1H, dd, *J* = 12.9 Hz, *J* = 3.3 Hz, H-3), 2.98 (1H, dd, *J* = 12.9 Hz, *J* = 16.8 Hz, H-3), 5.05 (2H, s, OCH_2_), 5.17 (2H, s, OCH_2_), 5.44 (1H, dd, *J* = 12.6, *J* = 3.3 Hz, H-2), 6.21 (2H, br s, H-6 and H-8), 7.28–7.66 (10H, m, H-2’’’–H-6’’’, H-2’’–H-6’’), 7.61 (2H, d, *J* = 7.5 Hz, H-2’ and H-3’), 7.72 (1H, d, *J* = 9.3 Hz, H-5’); ^13^C NMR (CDCl_3_, 75 MHz) *δ* 44.5 (C-3), 70.3 (OCH_2_), 70.4 (OCH_2_), 76.2 (C-2), 94.7 (C-6), 95.2 (C-8), 106.4 (C-2’, C-6’), 107.4 (C-10), 126.5 (C-2’’’, C-6’’’), 127.1 (C-2’’, C-6’’), 127.3 (C-4’’’), 127.5 (C-4’’), 128.5 (C-3’’’, C-5’’’), 129.3 (C-3’’, C-5’’), 131.7 (C-1’), 135.7 (C-4’), 136.3 (C-1’’’), 136.7 (C-1’’), 161.6 (C-9), 164.9 (C-5), 164.9 (C-7), 188.5 (C-4); MS (EI, 70 eV) *m/z* (%) 534 (10) [M + 2]^+^, 532 (9) [M]^+^, 442 (6), 359 (4), 256 (3), 91 (100); HRMS (EI, M^+^) calculated for C_29_H_22_O_4_BrF 532.0685 found 532.0671.

#### Synthesis and analytical data of 5,7-dibenzyloxy-2’-chloroflavanone (6l)


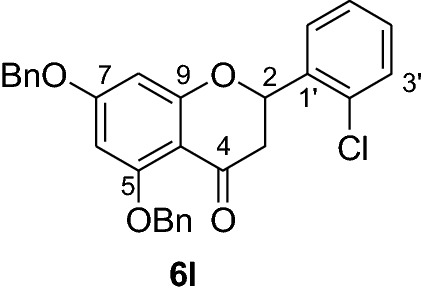


According to the general procedure II, 2’,4’-dibenzyloxy-6’-hydroxy-2-chlorochalcone (**5l**) (1.3 g, 2.9 mmol) was dissolved in ethanol (100 mL). Sodium acetate (1.9 g, 23 mmol) was added and the solution was refluxed for 48 h. The product **6l** was obtained in 42% yield. mp 127–128 °C; *R*_f_ 0.55 (petroleum ether:EtOAc 4:1); UV(MeOH) λ_max_: 272, 337 nm; IR *ν̃* [cm^−1^]: 1672 (conjugated C = O), 1604 (C = C), 1570 (C = C), 1162, 1068, and 1033 (C-O); ^1^H NMR (CDCl_3_, 300 MHz) *δ* 2.85 (1H, dd, *J* = 16.5, *J* = 3.3 Hz, H-3), 2.94 (1H, dd, *J* = 15.5, *J* = 3.3 Hz, H-3), 5.06 (2H, s, OCH_2_), 5.17 (2H, s, OCH_2_), 5.80 (1H, dd, *J* = 12.9 J = 3.3 Hz, H-2), 6.27 (2H, br s, H-6, H-8), 7.38 (11H, m, H-5’, H-2’’’–H-6’’’, H-2’’–H-6’’), 7.58 (1H, br s, H-6’), 7.60 (1H, d, *J* = 9.3 Hz, H-4’), 7.71 (1H, d, *J* = 9.3 Hz, H-3’); ^13^C NMR (CDCl_3_, 75 MHz) *δ* 44.5 (C-3), 70.3 (OCH_2_), 70.4 (OCH_2_), 76.4 (C-2), 94.7 (C-6), 95.2 (C-8), 106.4 (C-10), 126.3 (C-5’), 127.3 (C-2’’’, C-6’’’), 127.5 (C-2’’, C-6’’), 127.6 (C-4’’’), 128.3 (C-4’’), 128.5 (C-6’), 128.7 (C-3’’’, C-5’’’), 128.8 (C-3’’, C-5’’), 129.4 (C-4’), 129.6 (C-3’), 131.7 (C-2’), 135.7 (C-1’), 136.3 (C-1’’’), 136.7 (C-1’’), 161.1 (C-9), 164.9 (C-5), 165.2 (C-7), 188.5 (C-4); MS (EI, 70 eV) *m/z* (%) 470 (16) [M]^+^, 435 (4), 379 (11), 255 (4), 165 (3); HRMS (EI, M^+^) calculated for C_29_H_23_O_4_Cl 470.1285 found 470.1264.

#### Synthesis and analytical data of 5,7-dibenzyloxy-3’-chloroflavanone (6m)


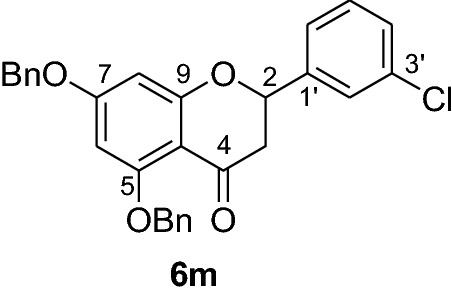


According to the general procedure II, 2’,4’-dibenzyloxy-6’-hydroxy-3-chlorochalcone (**5m**) (0.8 g, 1.8 mmol) was dissolved in ethanol (100 mL). Sodium acetate (1.2 g, 14.4 mmol) was added and the solution was refluxed for 48 h. The product **6m** was obtained in 50% yield. mp 129 °C; *R*_f_ 0.56 (petroleum ether:EtOAc 4:1); UV(MeOH) λ_max_: 274, 334 nm; IR *ν̃* [cm^−1^]: 1673 (conjugated C = O), 1604 (C = C), 1571 (C = C), 1428, 1163, 1117, and 1025 (C-O); ^1^H NMR (CDCl_3_, 300 MHz) *δ* 2.78 (1H, dd, *J* = 16.5 Hz, *J* = 3 Hz, H-3), 2.98 (1H, dd, *J* = 16.5 Hz, *J* = 13.2 Hz, H-3), 5.06 (2H, s, OCH_2_), 5.16 (2H, s, OCH_2_), 5.40 (1H, dd, *J* = 3 Hz, *J* = 12.9 Hz, H-2), 6.25 (2H, s, H-6, H-8), 7.36 (11H, m, H-6’, H-2’’’–H-6’’’, H-2’’–H-6’’), 7.49 (1H, br s, H-2’), 7.59 (2H, d, *J* = 7.8 Hz, H-4’, H-5’); ^13^C NMR (CDCl_3_, 75 MHz) *δ* 45.6 (C-3), 70.3 (OCH_2_), 70.3 (OCH_2_), 78.4 (C-2), 94.7 (C-6), 95.2 (C-8), 106.3 (C-10), 124.1 (C-6’), 126.2 (C-2’), 126.4 (C-2’’’, C-6’’’), 127.5 (C-2’’, C-6’’), 127.6 (4’’’), 128.1 (C-4’’), 128.3 (C-4’), 128.5 (C-3’’’, C-6’’’), 128.7 (C-3’’, C-5’’), 130.0 (C-5’), 134.7 (C-3’), 135.6 (C-1’’’), 136.2 (C-1’’), 140.8 (C-1’), 161.0 (C-9), 164.5 (C-5), 164.9 (C-7), 188.1 (C-4); MS (EI, 70 eV) *m/z* (%) 470 (26) [M]^+^, 379 (9), 348 (12), 306 (6), 257 (5), 215 (6), 91 (100); HRMS (EI, M^+^) calculated for C_29_H_23_O_4_Cl 470.1285 found 470.1270.

#### Synthesis and analytical data of 5,7-dibenzyloxy-2’,4’-dichloroflavanone (6n)


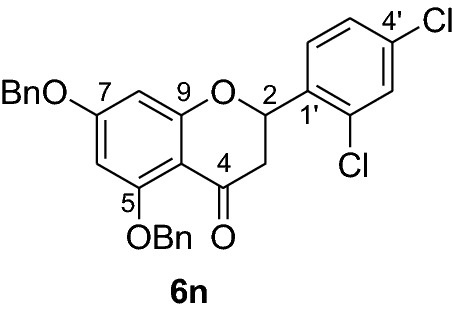


According to the general procedure II, 2’,4’-dibenzyloxy-6’-hydroxy-2,4-dichlorochalcone (**5n**) (0.6 g, 1.2 mmol) was dissolved in ethanol (100 mL). Sodium acetate (0.8 g, 9.6 mmol) and the solution was refluxed for 48 h. The product **6n** was obtained in 54% yield. mp 117–118 °C; *R*_f_ 0.57 (petroleum ether:EtOAc 4:1); UV(MeOH) λ_max_: 275, 339 nm; IR *ν̃* [cm^−1^]: 1669 (conjugated C = O), 1604 (C = C), 1570 (C = C), 1430, 1163, 1116, and 1040 (C-O); ^1^H NMR (CDCl_3_, 300 MHz) *δ* 2.79 (1H, dd, *J* = 12.9 Hz, *J* = 16.5 Hz, H-3), 2.93 (1H, dd, *J* = 16.5 Hz, *J* = 3.6 Hz, H-3), 5.08 (2H, s, OCH_2_), 5.17 (2H, s, OCH_2_), 5.77 (1H, dd, *J* = 12.9 Hz, *J* = 3.0 Hz, H-2), 6.25 (1H, d, *J* = 2.4 Hz, H-8), 6.27 (1H, d, *J* = 2.4 Hz, H-6), 7.39 (10H, m, H-2’’’–H-6’’’, H-2’’–H-6’’), 7.59 (2H, d, *J* = 7.2 Hz, H-3, H-5’), 7.64 (1H, d, *J* = 8.4 Hz, H-3’); ^13^C-NMR (CDCl_3_, 75 MHz) *δ* 44.4 (C-3), 70.3 (OCH_2_), 70.4 (OCH_2_), 75.7 (C-2), 94.7 (C-6), 95.3 (C-8), 106.4 (C-10), 126.5 (C-5’), 127.5 (C-2’’’, C-6’’’), 127.7 (C-3’’, C-6’’), 128.1 (C-4’’’), 128.3 (C-4’’), 128.5 (C-3’’’, C-5’’’), 128.7 (C-3’’, C-5’’), 128.9 (C-3’), 129.4 (C-6’), 132.3 (C-2’), 134.7 (C-4’), 135.4 (C-1’), 135.6 (C-1’’’), 136.2 (C-1’’), 161.1 (C-9), 164.6 (C-5), 164.9 (C-7), 188.0 (C-4); MS (EI, 70 eV) *m/z* (%) 506 (6) [M + 2]^+^, 504 (12) [M]^+^, 470 (3), 413 (7), 256 (3), 199 (6), 91 (100); HRMS (EI, M^+^) calculated for C_29_H_22_O_4_Cl_2_ 504.0895 found 504.0908.

#### Synthesis and analytical data of 5,7-dibenzyloxy-3’,5’-dichloroflavanone (6o)


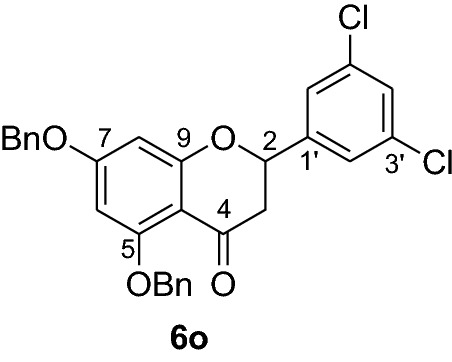


According to the general procedure II, 2’,4’-dibenzyloxy-6’-hydroxy-3,5-dichlorochalcone (**5o**) (0.7 g, 1.4 mmol) was dissolved in ethanol (100 mL). Sodium acetate (0.9 g, 11.2 mmol) was added and the solution was refluxed for 48 h. The product **6o** was obtained in 36% yield. mp 115–116 °C; *R*_f_ 0.58 (petroleum ether:EtOAc 4:1); UV(MeOH) λ_max_: 271, 338 nm; IR *ν̃* [cm^−1^]: 1668 (conjugated C = O), 1614 (C = C), 1572 (C = C), 1374, 1216, 1068, and 1018 (C-O); ^1^H NMR (CDCl_3_, 300 MHz) *δ* 2.80 (1H, dd, *J* = 3.3 Hz, *J* = 16.5 Hz, H-3), 2.95 (1H, dd, *J* = 16.5 Hz, *J* = 12.9 Hz), 5.06 (2H, s, OCH_2_), 5.16 (2H, s, OCH_2_), 5.36 (1H, dd, *J* = 12.9 Hz, *J* = 3.3 Hz, H-2), 6.21 (2H, br s, H-6, H-8), 7.33 (11H, m, H-4’, H-2’’’–H-6’’’, H-2’’–H-6’’), 7.57 (2H, d, *J* = 7.5 Hz, H-2’, H-6’); ^13^C NMR (CDCl_3_, 75 MHz) *δ* 45.5 (C-3), 70.4 (OCH_2_), 70.4 (OCH_2_), 77.7 (C-2), 94.7 (C-6), 95.5 (C-8), 106.3 (C-10), 124.5 (C-2’, C-6’), 126.5 (C-2’’’, C-6’’’), 127.6 (C-2’’, C-6’’), 127.7 (C-4’’’), 128.4 (C-4’’), 128.6 (C-3’’’, C-5’’’), 128.7 (C-3’’, C-5’’), 128.8 (C-4’), 135.4 (C-3’, C-5’), 135.6 (C-1’’’), 136.2 (C-1’’), 142.2 (C-1’), 161.1 (C-9), 164.2 (C-5), 165.0 (C-7), 187.6 (C-4); MS (EI, 70 eV) *m/z* (%) 506 (9) [M + 2]^+^, 504 (16) [M]^+^, 413(7), 384 (4), 359 (3), 303 (4), 256 (4), 199 (5), 91 (100); HRMS (EI, M^+^) calculated for C_29_H_22_O_4_Cl_2_ 504.0895 found 504.0889.

#### Synthesis and analytical data of 5,7-dibenzyloxy-2’,6’-dichloroflavanone (6p)


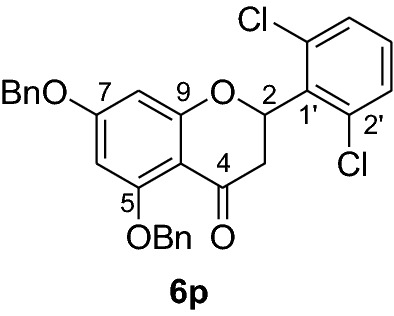


According to the general procedure II, 2’,4’-dibenzyloxy-6’-hydroxy-2,6-dichlorochalcone (**5p**) (0.7 g, 1.4 mmol) was dissolved in ethanol (100 mL). Sodium acetate (0.9 g, 11.2 mmol) was added and the solution was refluxed for 48 h. The product **6p** was obtained in 32% yield. mp 109–111 °C; *R*_f_ 0.53 (petroleum ether:EtOAc 4:1); UV(MeOH) λ_max_: 275, 339 nm; IR *ν̃* [cm^−1^]: 1663 (conjugated C = O), 1607 (C = C), 1570 (C = C), 1241, 1055, and 1029 (C-O);^1^H NMR (CDCl_3_, 500 MHz) *δ* 2.56 (1H, dd, *J* = 3.3 Hz, *J* = 16.5 Hz, H-3), 3.68 (1H, dd, *J* = 14.4 Hz, *J* = 16.8 Hz, H-3), 5.0 (2H, s, OCH_2_), 5.13 (2H, s, OCH_2_), 6.19 (1H, d, *J* = 1.8 Hz, H-8), 6.20 (1H, d, *J* = 1.8 Hz, H-6), 7.20 (1H, m, H-4), 7.35 (10H, m, H-2’’’–H-6’’’, H-2’’–H-6’’), 7.56 (2H, d, *J* = 7.5 Hz, H-3’, H-5’); ^13^C NMR (CDCl_3_, 125 MHz) *δ* 40.5 (C-3), 70.3 (OCH_2_), 70.4 (OCH_2_), 75.7 (C-2), 94.5 (C-6), 95.1 (C-8), 106.2 (C-10), 126.5 (C-3’, C-5’), 127.6 (C-2’’’, C-6’’’), 128.3 (C-2’’, C-6’’), 128.5 (C-4’’’), 128.6 (C-4’’), 129.6 (C-3’’’, C-5’’’), 130.1 (C-3’’, C-5’’), 132.4 (C-4’), 132.5 (C-2’, C-6’), 135.1 (C-1’), 135.7 (C-1’’’), 136.3 (C-1’’), 161.2 (C-9), 164.6 (C-5), 164.9 (C-7), 188.2 (C-4); MS (EI, 70 eV) *m/z* (%) 506 (10) [M + 2]^+^, 504 (17) [M]^+^, 469 (6), 413 (11), 359 (14), 305 (6), 256 (4), 199 (16), 91 (100); HRMS (EI, M^+^) calculated for C_29_H_22_O_4_Cl_2_ 504.0895 found 504.0881.

#### Synthesis and analytical data of 5,7-dibenzyloxy-2’,5’-dichloroflavanone (6q)


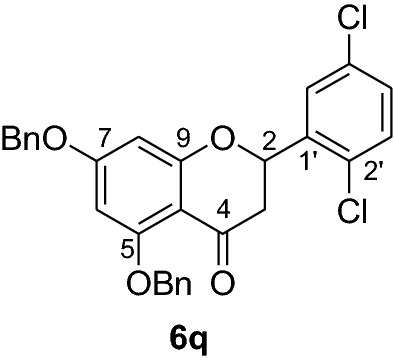


According to the general procedure II, 2’,4’-dibenzyloxy-6’-hydroxy-2,6-dichlorochalcone (**5q**) (0.7 g, 1.4 mmol) was dissolved in ethanol (100 mL). Sodium acetate (0.9 g, 11.2 mmol) was added and the solution was refluxed for 48 h. The product **6q** was obtained in 43% yield. mp 121–124 °C; *R*_f_ 0.55 (petroleum ether:EtOAc 4:1); UV(MeOH) λ_max_: 273, 338 nm; IR *ν̃* [cm^−1^]:1671 (conjugated C = O), 1606 (C = C), 1570 (C = C), 1466, 1209, 1108, and 1032 (C-O); ^1^H NMR (CDCl_3_, 500 MHz) *δ* 2.78 (1H, dd, *J* = 13.5 Hz, *J* = 17.0 Hz, H-3), 2.96 (1H, dd, *J* = 16.5 Hz, *J* = 2.5 Hz, H-3), 5.07 (2H, s, OCH_2_), 5.17 (2H, s, OCH_2_), 5.75 (1H, dd, *J* = 13.5 Hz, *J* = 3 Hz, H-2), 6.28 (1H, d, *J* = 2 Hz, H-8), 6.29 (1H, d, *J* = 2 Hz, H-6), 7.29 (1H, dd, *J* = 8.5 Hz, *J* = 2.5 Hz, H-4’), 7.39 (10H, m, H-2’’’–H-6’’’, H-2’’–H-6’’), 7.59 (1H, d, *J* = 7.5 Hz, H-3’), 7.72 (1H, d, *J* = 2.5 Hz, H-6’); ^13^C NMR (CDCl_3_, 125 MHz) *δ* 44.4 (C-3), 70.4 (OCH_2_), 70.4 (OCH_2_), 75.9 (C-2), 94.8 (C-6), 95.5 (C-8), 106.4 (C-10), 126.5 (C-2’’’, C-6’’’), 126.5 (C-2’’, C-6’’), 127.2 (C-4’’’), 127.5 (C-4’’), 127.7 (C-6’), 128.4 (C-3’’’, C-5’’’), 128.6 (C-3’’, C-5’’), 129.4 (C-4’), 129.6 (C-3’), 130.8 (C-2’), 133.4 (C-5’), 135.7 (C-1’), 136.2 (C-1’’’), 138.5 (C-1’’), 161.2 (C-9), 164.5 (C-5), 165.0 (C-7), 187.9 (C-4); MS (EI, 70 eV) *m/z* (%) 506 (34) [M + 2]^+^, 504 (49) [M]^+^, 469 (16), 413 (31), 385 (9), 359 (12), 305 (15), 256 (10), 199 (14), 91 (100); HRMS (EI, M^+^) calculated for C_29_H_22_O_4_Cl_2_ 504.0895 found 504.0863.

#### Synthesis and analytical data of 5,7-dibenzyloxy-3’,4’-dichloroflavanone (6r)


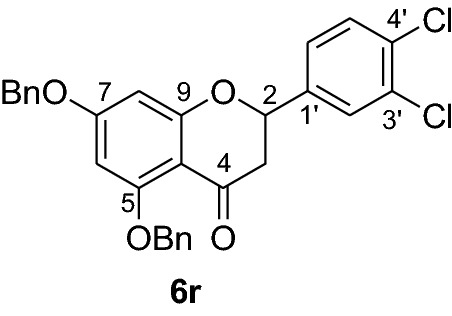


According to the general procedure II, 2’,4’-dibenzyloxy-6’-hydroxy-2,6-dichlorochalcone (**5r**) (0.9 g, 1.8 mmol) was dissolved in ethanol (100 mL). Sodium acetate (1.2 g, 14.4 mmol) was added and the solution was refluxed for 48 h. The product **6r** was obtained in 44% yield. mp 123–125 °C; *R*_f_ 0.54 (petroleum ether:EtOAc 4:1); UV(MeOH) λ_max_: 271, 337 nm; IR *ν̃* [cm^−1^]: 1666 (conjugated C = O), 1606 (C = C), 1431, 1165, 1118, and 1028 (C-O); ^1^H NMR (CDCl_3_, 500 MHz) *δ* 2.81 (1H, dd, *J* = 16.5 Hz, *J* = 3.0 Hz, H-3), 2.96 (1H, dd, *J* = 13 Hz, *J* = 16.5 Hz, H-3), 5.06 (2H, s, OCH_2_), 5.16 (2H, s, OCH_2_), 5.38 (1H, dd, *J* = 13 Hz, *J* = 3 Hz, H-3), 6.24 (1H, d, *J* = 1.5 Hz, H-8), 6.26 (1H, d, *J* = 1.5 Hz, H-6), 7.28 (1H, d, *J* = 2 Hz, H-2’), 7.29–7.39 (10H, m, H-2’’’–H-6’’’, H-2’’–H-6’’), 7.50 (1H, d, *J* = 8.5 Hz, H-5’), 7.58 (1H, dd, *J* = 7.5 Hz, *J* = 2 Hz, H-6’); ^13^C NMR (CDCl_3_, 125 MHz) *δ* 45.5 (C-3), 70.3 (OCH_2_), 70.4 (OCH_2_), 77.8 (C-2), 94.7 (C-6), 95.4 (C-8), 106.3 (C-10), 125.2 (C-6’), 126.5 (C-2’’’, C-6’’’), 127.5 (C-2’’, C-6’’), 127.6 (C-4’’’), 127.7 (C-4’’), 128.1 (C-2’), 128.4 (C-3’’’, C-5’’’), 128.5 (C-3’’, C-5’’), 128.6 (C-5’), 128.7 (C-4’), 130.7 (C-3’), 135.2, (C-1’), 136.2 (C-1’’’), 139.0 (C-1’’), 161.1 (C-9), 164.3 (C-5), 165.0 (C-7), 187.8 (C-4); MS (EI, 70 eV) *m/z* (%) 506 (1) [M + 2]^+^, 504 (2) [M]^+^, 413 (1), 348 (17), 306 (7), 257 (7), 215 (8), 180 (7), 91 (100); HRMS (EI, M^+^) calculated for C_29_H_22_O_4_Cl_2_ 504.0895 found 504.0871.

### Synthesis of flavanones 7a-l by deprotection of the dibenzylated flavanones 6a-r

#### General Procedure III for the synthesis of 7a-l

A dibenzylated flavanone **6** was dissolved in sufficient EtOAc:EtOH (1:1) to produce a 0.01 M solution. The resulting solution was subjected to hydrogenolysis using a H-Cube Pro over 10%Pd/C at a flow rate of 1 mL/min at 70 °C and 1 bar. The solution was concentrated *in vacuo* and purified by column chromatography on silica gel.

#### Synthesis and analytical data of pinocembrin (7a)


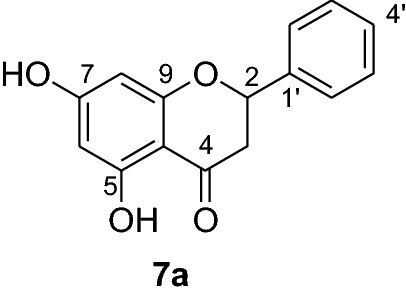


Following general procedure III, 5,7-dibenzyloxyflavanone (**6a**) (100 mg, 0.23 mmol) was debenzylated and the crude product was purified by column chromatography over silica gel with petroleum ether:EtOAc (7:3) as the eluent to furnish **7a** as white solid in 93% yield. mp 193–194 °C; *R*_f_ 0.68 (petroleum ether:EtOAc 4:1); UV(MeOH) λ_max_: 290, 335 nm; IR *ν̃* [cm^−1^]: 1628 (C = C), 1581 (C = C), 1453, 1300, 1085, and 1063 (C-O); ^1^H NMR (CD_3_OD, 300 MHz) *δ* 2.66 (1H, dd, *J* = 3.3 Hz, *J* = 17.4 Hz, H-3), 2.92 (1H, dd, *J* = 12.9 Hz, *J* = 17.1 Hz, H-3), 5.25 (2H, dd, *J* = 3 Hz, *J* = 12.9 Hz, H-2), 5.96 (1H, d, *J* = 2.4 Hz, H-6), 6.01 (1H, d, *J* = 2.1 Hz, H-8), 7.30 (5H, m, H-2’–H-6’); ^13^C NMR (CD_3_OD, 75 MHz) *δ* 42.7 (C-3), 78.9 (C-2), 95.6 (C-8), 96.2 (C-6), 101.6 (C-10), 125.4 (C-3’, C-5’), 128.5 (C-2’, C-4’, C-6’), 137.3 (C-1’), 162.5 (C-9), 163.9 (C-5), 167.4 (C-7), 195.4 (C-4); MS (EI, 70 eV) *m/z* (%) 256 (100) [M]^+^, 179 (66), 152 (46), 124 (16).

#### Synthesis and analytical data of 2’,6’-dimethoxyflavanone (7b)


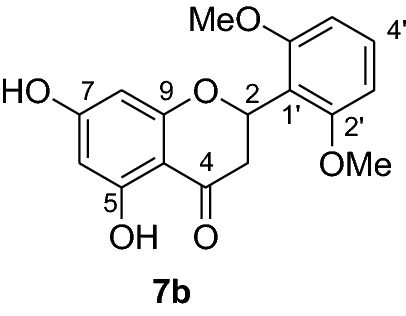


Following general procedure III, 5,7-dibenzyloxy-2’,6’-dimethoxyflavanone (**6d**) (60 mg, 0.12 mmol) was debenzylated and the crude product was purified by column chromatography over silica gel with petroleum ether:EtOAc (7:3) as the eluent to furnish **7b** in 78% yield as a white solid. mp 193–194 °C; UV(MeOH) λ_max_: 291, 343 nm; ^1^H NMR (DMSO-d_6_, 500 MHz) *δ* 2.78, (1H, dd, *J* = 3 Hz, *J* = 17 Hz, H-3), 3.05 (1H, dd, *J* = 17.5 Hz, *J* = 13 Hz, H-3), 3.78 (3H, s, OMe), 3.83 (3H, s, OMe), 5.75 (1H, dd, *J* = 2.5 Hz, *J* = 12.5 Hz, H-2), 5.98 (1H, d, *J* = 2 Hz, H-6), 6.02 (1H, d, *J* = 2.5 Hz, H-8), 6.92 (1H, dd, *J* = 3 Hz, *J* = 9 Hz, H-3’), 7.01 (1H, d, *J* = 9 Hz, H-4’), 7.81 (1H, d, *J* = 3 Hz, H-5’), 12.16 (1H, s, 5-OH); MS (EI, 70 eV) *m/z* (%) 316 (56) [M]^+^, 285 (100), 179 (11), 164 (24), 151 (28), 121 (25), 77 (21), 73 (24), 60 (33).

#### Synthesis and analytical data of 4’-chloroflavanone (7c)

Following general procedure III, 5,7-dibenzyloxy-4’-chloroflavanone (**6c**) (180 mg, 0.38 mmol) was debenzylated and the crude product was purified by column chromatography over silica with petroleum ether:EtOAc (7:3) as the eluent to furnish **7c** as a white solid in 91% yield. MS (EI, 70 eV) *m/z* (%) 290 (33) [M]^+^, 256 (73), 255 (100), 179 (73), 152 (51), 124 (27).

#### Synthesis and analytical data of 3’,4’,5’-trimetoxyflavanone (7d)


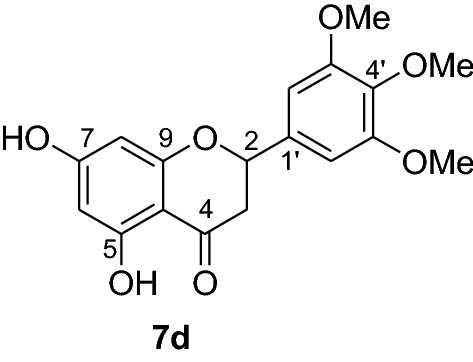


Following general procedure III, 5,7-dibenzyloxy-3’,4’,5’-trimetoxyflavanone (**6h**) (60 mg, 0.12 mmol) was debenzylated and the crude product was purified by column chromatography over silica gel with petroleum ether:EtOAc (7:3) as the eluent to furnish **7d** as a white solid in 89% yield. mp 178–179 °C; UV(MeOH) λ_max_: 293, 347 nm; ^1^H NMR (DMSO-d_6_, 500 MHz) *δ* 2.78, (1H, dd, *J* = 3 Hz, *J* = 17.5 Hz, H-3), 3.21 (1H, dd, *J* = 13 Hz, *J* = 14 Hz, H-3), 3.74 (3H, s, OMe), 3.86 (6H, s, OMe), 5.47 (1H, dd, *J* = 3 Hz, *J* = 13 Hz, H-2), 5.96 (1H, d, *J* = 2 Hz, H-6), 5.99 (1H, d, *J* = 2 Hz, H-8), 6.90 (2H, s, H-2’, H-6’), 12.16 (1H, s, 5-OH); MS (EI, 70 eV) *m/z* (%) 346 (39) [M]^+^, 303 (4), 194 (12), 181 (100).

#### Synthesis and analytical data of 2’,3’-dichloroflavanone (7e)


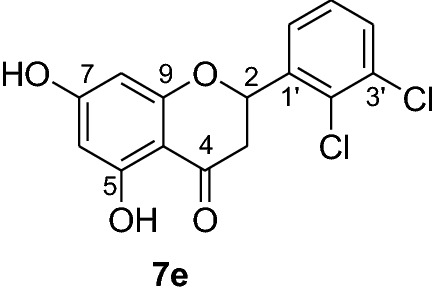


Following general procedure III, 5,7-dibenzyloxy-2’,3’-dichloroflavanone (**6j**) (80 mg, 0.16 mmol) was debenzylated and the crude product was purified by column chromatography over silica gel with petroleum ether:EtOAc (7:3) as the eluent to furnish **7e** as a white solid in 88% yield. ^1^H NMR (DMSO-d_6_, 500 MHz) *δ* 2.91 (1H, dd, *J* = 3 Hz, *J* = 17.5 Hz, H-3), 3.11 (1H, dd, *J* = 13 Hz, *J* = 17 Hz, H-3), 5.94 (1H, dd, *J* = 3 Hz, *J* = 13 Hz, H-2), 6.00 (1H, d, *J* = 2 Hz, H-6), 6.03 (1H, d, *J* = 2 Hz, H-8), 7.53 (1H, t, *J* = 8 Hz, H-5’), 7.65 (1H, dd, *J* = 1.5 Hz, *J* = 8 Hz, H-4’), 7.78 (1H, dd, *J* = 1 Hz, *J* = 7 Hz, H-6’), 12.05 (1H, s); MS (EI, 70 eV) *m/z* (%) 326 (31) [M + 2]^+^, 324 (51) [M]^+^, 289 (94), 255 (14), 179 (100), 152 (66), 124 (46).

#### Synthesis and analytical data of 2’-chloroflavanone (7f)


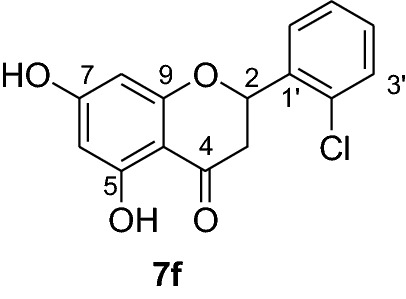


Following general procedure III, 5,7-dibenzyloxy-2’-chloroflavanone (**6 l**) (270 mg, 0.57 mmol) was debenzylated and the crude product was purified by column chromatography over silica gel with petroleum ether:EtOAc (7:3) as the eluent to furnish **7f** as a white solid in 92% yield. ^1^H NMR (CD_3_OD, 300 MHz) *δ* 2.84 (1H, dd, *J* = 3 Hz, *J* = 17.1 Hz, H-3), 3.08 (1H, dd, *J* = 12.3 Hz, *J* = 17.1 Hz, H-3), 5.52 (1H, dd, *J* = 2.4 Hz, *J* = 12.3 Hz, H-2), 5.83 (1H, d, *J* = 3 Hz, H-6), 5.86 (1H, d, *J* = 3 Hz, H-8), 5.97 (4H, m, H-3’–H-6’); ^13^C-NMR (CD_3_OD, 75 MHz) *δ* 44.3 (C-3), 79.7 (C-2), 96.2 (C-6), 96.3 (C-8), 103.3 (C-10), 125.5 (C-5’), 127.5 (C-6’), 129.5 (C-3’), 131.3 (C-4’), 135.6 (C-2’), 142.7 (C-1’), 164.2 (C-9), 165.5 (C-5), 168.3 (C-7), 196.8 (C-4); MS (EI, 70 eV) *m/z* (%) 290 (33) [M]^+^, 256 (73), 255 (100), 179 (73), 152 (51), 124 (27).

#### Synthesis and analytical data of 3’-chloroflavanone (7g)


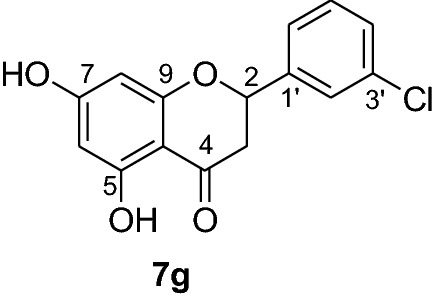


Following general procedure III, 5,7-dibenzyloxy-3’-chloroflavanone (**6m**) (10 mg, 0.02 mmol) was debenzylated and the crude product was purified by column chromatography over silica gel with petroleum ether:EtOAc (7:3) as the eluent to furnish **7g** as a white solid in 78% yield. *R*_f_ 0.68 (petroleum ether:EtOAc 4:1); IR ʋ_cm-1:_ 1628 (C = C), 1581 (C = C), 1453, 1300, 1085 and 1063 (C-O); ^1^H NMR (CD_3_OD, 300 MHz) *δ* 2.84, (1H, m, H-3), 3.11 (1H, m, H-3), 5.50 (1H, dd, *J* = 4.5 Hz, *J* = 9.6 Hz, H-2), 5.97 (1H, d, *J* = 1.8 Hz, H-6), 6.00 (1H, d, *J* = 1.8 Hz, H-8), 7.52 (4H, m, H-2’, H-3’–H-6’); ^13^C NMR (CD_3_OD, 75 MHz) *δ* 42.6 (C-3), 78.3 (C-2), 94.7 (C-6), 95.8 (C-8), 102.1 (C-10), 124.1 (C-6’), 126.2 (C-2’), 128.7 (C-4’), 133.9 (C-5’), 138.9 (C-3’), 141.3 (C-1’), 162.9 (C-9), 164.4 (C-5), 166.8 (C-7), 195.9 (C-4); MS (EI, 70 eV) *m/z* (%) 290 (33) [M]^+^, 256 (63), 179 (100), 152 (62), 124 (37).

#### Synthesis and analytical data of 2’,4’-dichloroflavanone (7h)


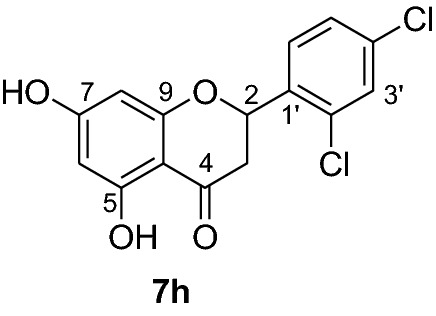


Following general procedure III, 5,7-dibenzyloxy-2’,4’-dichloroflavanone (**6n**) (160 mg, 0.12 mmol) was debenzylated and the crude product was purified by column chromatography over silica gel with petroleum ether:EtOAc (7:3) as the eluent to furnish **7h** as a white solid in 87% yield. ^1^H NMR (CD_3_OD, 500 MHz) *δ* 2.87, (1H, dd, *J* = 2.5 Hz, *J* = 14 Hz, H-3), 3.02 (1H, dd, *J* = 14 Hz, *J* = 16 Hz, H-3), 5.80 (1H, dd, *J* = 2.5 Hz, *J* = 13 Hz, H-2), 5.96 (1H, br s, H-6), 5.99 (1H, br s, H-8), 7.48 (1H, d, *J* = 8.5 Hz, H-6’), 7.57 (1H, br s, H-3’), 7.77 (1H, dd, *J* = 1.2 Hz, *J* = 8.5 Hz, H-5’); ^13^C NMR (CD_3_OD, 125 MHz) *δ* 42.6 (C-3), 76.9 (C-2), 95.8 (C-6), 97.5 (C-8), 129.0 (C-5’), 129.8 (C-6’), 130.8 (C-3’), 133.2 (C-2’), 135.7 (C-4’), 136.7 (C-1’), 164.9 (C-9), 165.8 (C-5), 168.2 (C-7), 196.2 (C-4); MS (EI, 70 eV) *m/z* (%) 326 (24) [M + 2]^+^, 324 (32) [M]^+^, 289 (74), 256 (68), 179 (100), 152 (83), 124 (52), 69 (54).

#### Synthesis and analytical data of 3’,5’-dichloroflavanone (7i)


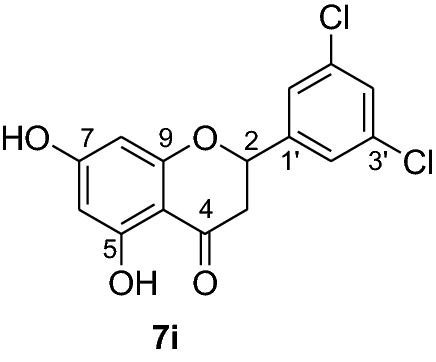


Following general procedure III, 5,7-dibenzyloxy-3’,5’-dichloroflavanone (**6o**) (200 mg, 0.4 mmol) was debenzylated and the crude product was purified by column chromatography over silica gel with petroleum ether:EtOAc (7:3) as the eluent to furnish **7i** as a white solid in 89% yield. ^1^H NMR (CD_3_OD, 500 MHz) *δ* 2.87, (1H, dd, *J* = 3 Hz, *J* = 17 Hz, H-3), 3.06 (1H, dd, *J* = 12.5 Hz, *J* = 17 Hz, H-3), 5.51 (1H, dd, *J* = 3 Hz, *J* = 12.5 Hz, H-2), 5.95 (1H, d, *J* = 2 Hz, H-6), 6.02 (1H, d, *J* = 2.5 Hz, H-8), 7.49 (1H, t, *J* = 1.75 Hz, H-4’), 7.53 (2H, m, H-2’, H-6’); ^13^C NMR (CD_3_OD, 125 MHz) *δ* 42.2 (C-3), 77.2 (C-2), 94.7 (C-6), 96.0 (C-8), 124.5 (C-2’, C-6’), 127.9 (C-4’), 135.0 (C-3’, C-5’), 143.0 (C-1’), 162.6 (C-9), 164.1 (C-5), 167.1 (C-7), 196.8 (C-4); MS (EI, 70 eV) *m/z* (%) 326 (22) [M + 2]^+^, 324 (36) [M] + , 289 (79), 256 (64), 255 (68), 179 (100), 152 (86), 124 (48),, 69 (52), 57 (97).

#### Synthesis and analytical data of 2’,6’-dichloroflavanone (7j)


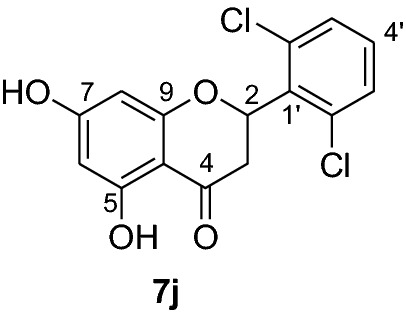


Following general procedure III, 5,7-dibenzyloxy-2’,6’-dichloroflavanone (**6p**) (140 mg, 0.28 mmol) was debenzylated and the crude product was purified by column chromatography over silica gel with petroleum ether:EtOAc (7:3) as the eluent to furnished **7j** as a white solid in 91% yield. ^1^H NMR (CD_3_OD/CDCl_3_, 500 MHz) *δ* 2.59, (1H, dd, *J* = 3.5 Hz, *J* = 17.5 Hz, H-3), 3.74 (1H, dd, *J* = 14.5 Hz, *J* = 17.5 Hz, H-3), 5.92 (1H, d, *J* = 2 Hz, H-6), 5.94 (1H, d, *J* = 2 Hz, H-8), 6.22 (1H, dd, *J* = 3.5 Hz, *J* = 14.5 Hz, H-2), 7.35 (1H, dd, *J* = 7.5 Hz, *J* = 7.5 Hz, H-4’), 7.45 (2H, br d, *J* = 7.5 Hz, H-3’, H-5’); ^13^C NMR (CD_3_OD/CDCl_3_, 125 MHz) *δ* 39.2 (C-3), 76.2 (C-2), 95.8 (C-6), 97.2 (C-8), 102.8 (C-10), 126.3 (C-5’, C-5’), 130.5 (C-4’), 131.8 (C-1’), 136.5 (C-2’, C-6’), 164.1 (C-9), 165.2 (C-5), 168.2 (C-7), 195.9 (C-4); MS (EI, 70 eV) *m/z* (%) 325 (62) [M]^+^, 323 (75), 289 (96), 256 (31), 179 (100), 151 (70), 124 (36).

#### Synthesis and analytical data of 2’,5’-dichloroflavanone (7k)


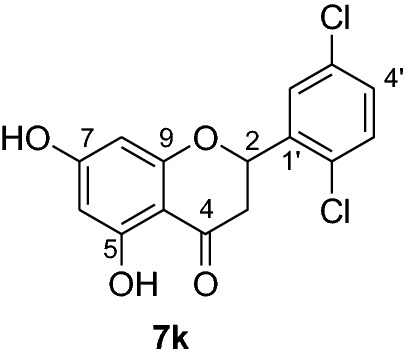


Following general procedure III, 5,7-dibenzyloxy-2’,5’-dichloroflavanone (**6q**) (115 mg, 0.23 mmol) was debenzylated and the crude product was purified by column chromatography over silica gel with petroleum ether:EtOAc (7:3) as the eluent to furnish **7k** as a white solid in 94% yield. ^1^H NMR (CD_3_OD, 500 MHz) *δ* 2.90, (1H, dd, *J* = 3 Hz, *J* = 17 Hz, H-3), 3.01 (1H, dd, *J* = 13 Hz, *J* = 17 Hz, H-3), 5.80 (1H, dd, *J* = 3 Hz, *J* = 13 Hz, H-2), 5.98 (1H, d, *J* = 2 Hz, H-6), 6.04 (1H, d, *J* = 2.5 Hz, H-8), 7.43(1H, dd, *J* = 2.5 Hz, *J* = 8.5 Hz, H-4’), 7.50 (1H, d, *J* = 9 Hz, H-3’), 7.78 (1H, d, *J* = 2.5 Hz, H-6’); ^13^C NMR (CD_3_OD, 125 MHz) *δ* 41.2 (C-3), 75.5 (C-2), 95.1 (C-6), 96.1 (C-8), 102.1 (C-10), 127.2 (C-2’), 130.8 (C-4’), 131.1 (C-3’), 133.5 (C-2’), 133.6 (C-5’), 138.8 (C-1’), 163.0 (C-9), 164.1 (C-5), 167.1 (C-7), 194.3 (C-4); MS (EI,7 0 eV) *m/z* (%) 326 (24) [M + 2]^+^, 324 (32) [M]^+^, 290 (17), 256 (34), 179 (100), 152 (70), 124 (42).

#### Synthesis and analytical data of 3’,4’-dichloroflavanone (7l)


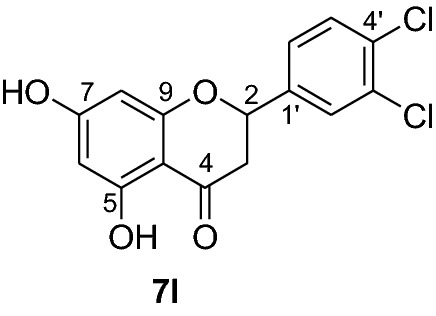


Following general procedure III, 5,7-dibenzyloxy-3’,4’-dichloroflavanone (**6r**) (120 mg, 0.24 mmol) was debenzylated and the crude product purified by column chromatography over silica gel with petroleum ether:EtOAc (7:3) as the eluent to furnish **7 l** as a white solid in 88% yield. ^1^H NMR (CD_3_OD, 500 MHz) *δ* 2.85, (1H, dd, *J* = 3.5 Hz, *J* = 17 Hz, H-3), 3.09 (1H, dd, *J* = 12.5 Hz, *J* = 17 Hz, H-3), 5.52 (1H, dd, *J* = 3.5 Hz, *J* = 13 Hz, H-2), 5.95 (1H, d, *J* = 2.5 Hz, H-6), 6.00 (1H, d, *J* = 2 Hz, H-8), 7.47 (1H, dd, *J* = 2.5 Hz, *J* = 8.5 Hz, H-6’), 7.61 (1H, d, *J* = 8.5 Hz, H-5’), 7.75 (1H, d, *J* = 2 Hz, H-2’); MS (EI, 70 eV) *m/z* (%) 326 (24) [M + 2]^+^, 324 (32) [M]^+^, 290 (17), 256 (34), 179 (100), 152 (70), 124 (42).

### Experimental design for antiplasmodial assay

This experiment was done on Swiss albino mice of the age of 6–8 weeks and body weight ranges from 22–28 g which were reared in the Animal House of College of Natural and Computational Science, Addis Ababa University. The mice were handled according to Guide for the Care and Use of Laboratory Animals [31].They were given a standard food and water (ad libitum) and maintained in 12 h dark and 12 h light (artificial light). The experiment was done on male mice. The mice have been acclimatized for a week before the experiment started.

#### Preparation of *Plasmodium* parasite

The *Plasmodium berghei* parasite was obtained from Ethiopian Public Health Institute (EPHI) and was passaged on a weekly basis from infected mice to healthy mice. For the experiment four donor mice were prepared by infecting *P. berghei* through intraperitoneal injection of the blood of infected mice with normal saline. When the parasitaemia was confirmed to be 30–40% by preparing a blood smear, blood was taken from the hearts of the donor mice after anesthetized them. The blood was taken from each donor mouse and diluted with physiological saline (0.85%) in 1:4 ratio.

#### In vivo antiplasmodial experiment

Standard four days suppression test was used to evaluate the antiplasmodial activity of the synthetic compounds [18]. The mice in this study were grouped into treatment, positive and negative control groups. Each group contains five mice. On D0, all the mice were weighted on a sensitive balance and the packed cell volume (PCV) were measured. Then each mouse in the treatment and control group was injected with 0.2 ml of blood diluted with physiological saline (intraperitoneally) which contains approximately 1 × 10^7^ *P. berghei* parasitized RBCs.

#### Administration of pinocembrin and its analogs

After three hours of injection of *P. berghei* infected blood, synthetic pinocembrin and its analogs were administered each separately. Compound **6a** was administered at doses of 15, 20, 25, 30 and 35 mg/kg b. wt. Compounds **6b**, **6e** and **6c** were tested at a dose of 20 mg/kg b. wt each. Compound **6i** was given at doses of 15, 25 and 35 mg/kg b. wt. Compound **7a** was given at doses of 20 and 30 mg/kg b. wt. Chloroquine (CQ) 25 mg/kg b.wt and 1 ml/100 g of 10% DMSO were given as a positive and a negative control respectively. The administration continued for 4 days (D0–D3) based on modified procedure of Peters (1965) [18]. The amount of the compounds administration was based on the availability of the synthesized quantity.

#### Percent parasitaemia and percent suppression

On D4, blood was taken gently from the tail of each mouse on a slide to make a blood smear. The smear was fixed with methanol and stained with 10% Giemsa stain for 30 min, then washed with water. Each blood smear was examined under the compound microscope with oil emulsion at magnification power of 100 × (together with ocular lens 10,000 ×) and each slide was counted for 5–10 times. The percent parasitaemia and percent suppression was calculated by the following formula as given in Peters [18].

$$\% {\text{ Parasitaemia}} = \frac{{number\,of\,infected\,RBC}}{{Number\,of\,infected\,RBC\, + \,number\,of\,uninfected\,RBC}} \times { 1}00.$$

$$\% {\text{ Suppression}}\, = \,\frac{{parasitaemia\,in\,negative\,control - \,parasitaemia\,in\,treatment\,group}}{{Parasitaemia\,in\,negative\,control}} \times { 1}00.$$

#### Determination of mean survival time

All the mice in the treatment and control groups were followed daily starting from the day of parasite injection and MST was calculated by using the following formula given below.

$${\text{MST}} = \frac{{sum\,of\,survival\,day\,of\,all\,mice\,in\,a\,group\,\left( {day} \right)}}{{Total\,number\,of\,mice\,in\,that\,group}}$$

#### Body weight and PCV determination

Body weight and PCV were measured on D0 and D4 to evaluate the effect of the compounds on the mice. The PCV was measured by taking blood ¾ of heparinized microhematocrit capillary tube (75 mm) from the tail of the mice. The tube was sealed with sealant and centrifuged on microhematocrit centrifuge (MK IV, England) by 13,000 rpm for 4 min. Then, the total blood volume and the volume of erythrocyte were measured by using a ruler. Mean body weight was also calculated using the following formula.

$${\text{Meanbody weight}}\, = \,\frac{{Total\,weight\,of\,mice\,in\,a\,group}}{{Total\,number\,of\,mice\,in\,that\,group}}$$

The PCV was calculated using the formula given by Gilmour and Sykes (1951).

$${\text{PCV}}\, = \,\frac{{Volume\,of\,erythrocyte\,in\,a\,given\,blood}}{{Total\,blood\,volume}} \times {1}00.$$

### Molecular docking simulation of compounds 7a-l against *Plasmodium falciparum* dihydrofolate reductase-thymidylate synthase

AutoDock Vina with standard protocol was used to dock the proteins (PfDHFR-TS) (PDB ID 1J3I) and compounds synthesized (**7a-l**) into the active site of proteins [32, 33]. ChemOffice tool (Chem Draw 16.0) was used to draw the chemical structures of the synthesized compounds (**7a-l**) while the proper 2D orientation, and energy of each molecule was minimized using ChemBio3D. The energy minimized ligand molecules were then used as input for AutoDock Vina, in order to carry out the docking simulation. The crystal structure of *Plasmodium falciparum* dihydrofolate reductase-thymidylate synthase was downloaded from protein data bank. The protein preparation was by removing the co-crystallized ligand, selected water molecules and cofactors, the target protein file was prepared by leaving the associated residue with protein by using Auto preparation of target protein file AutoDock 4.2 (MGL tools1.5.7). The grid box for docking simulations was setted using graphical user interface program. The grid was set so that it surrounds the region of interest in the macromolecule. The best docked conformation between ligand and protein was searched using the docking algorithm with AutoDock Vina. During the docking process, a maximum of nine conformers were considered for each ligand. The conformations with the most favorable (least) free binding energy were selected for analyzing the interactions between the target receptor and ligands by Discovery studio visualizer and PyMOL. The ligands are represented in different color, H-bonds and the interacting residues are represented in stick model representation.

### In silico drug-likeness predictions

In silico Drug-likeness helps to know whether a particular pharmacological agent has properties consistent with being an orally active drug. This prediction is based on an already established rule called Lipinski rule of five [29]. The structures of compounds synthesized (**7a-l**) were changed to their canonical simplified molecular input line entry system (SMILE) then submitted to SwissADME tool to estimate in silico pharmacokinetic parameters and other molecular properties based on the methodology reported by Amina et al. 2016. The data obtained were compared with chloroquine (standard drug), and only compounds without violation of any of the screenings were used for the molecular docking analysis.

### Ethical consideration

To conduct this study, ethical clearance was obtained from Institutional Ethics Review Board of College of Natural Sciences, Addis Ababa University (IRB/024/2017). The study is reported in accordance with the Animal Research Reporting of in vivo Experiments (ARRIVE) guidelines (https://arriveguidelines.org) and were handled according to the Guide for the Care and Use of Laboratory Animals (https://grants.nih.gov/grants/olaw/guide-for-the-care-and-use-of-laboratory-animals).

### Data analysis

The data generated in this study were analyzed using IBM SPSS, version 20 statistical package. The results were presented as mean ± SEM (standard error of the mean). One-way ANOVA (analysis of variance), paired Student’s *t*-test and Kaplan–Meier analyses were used for the statistical analysis. A statistically significant difference was taken at P-value less than 0.05 (P < 0.05).

## Supplementary Information

**Additional file 1. **The NMR spectra and molecular docking simulations for the synthetic compounds are included within Additional materials (Additional files 1 and 2).

**Additional file 2. **The NMR spectra and molecular docking simulations for the synthetic compounds are included within Additional materials (Additional file 1 and 2).

## Data Availability

The datasets supporting the findings of this article are presented in the main manuscript. The MS and IR spectra of the synthesized compounds along with the molecular docking simulations of compound **7a-j** can be accessed from the corresponding author on reasonable request. "The X-ray structure of Wild-type *Plasmodium falciparum* dihydrofolate reductase-thymidylate synthase (PfDHFR-TS) complexed with WR99210, NADPH, and dUMP (PDB code 1J3I) was obtained from the Protein Data Bank. The target protein was downloaded from the protein data bank and no homology or structural determination was done. The structures are searchable in the protein data bank repository using (PDB ID: 1J3I) (https://www.rcsb.org/structure/1J3I). The PDB ID was given in the method section. The NMR spectra and molecular docking interaction of the compounds synthesized against the protein PfDHFR-TS (https://www.rcsb.org/structure/1J3I) are provided as Additional files 1 and 2, respectively.
