# Supplementary material for: Synthesis, antiplasmodial activity and in silico molecular docking study of pinocembrin and its analogs
Source: BMC Chem. 2022 May 24;16(1):36. doi: 10.1186/s13065-022-00831-z (PMC9128099; doi:10.1186/s13065-022-00831-z)
Supplement: Supplementary file 1 — Additional file 1. The NMR spectra and molecular docking simulations for the synthetic compounds are included within Additional materials (Additional files 1 and 2). [file 13065_2022_831_MOESM1_ESM.docx]

**SUPPLEMENTARY DATA**

**Synthesis, Antiplasmodial Activity and *In silico* Molecular Docking Study of Pinocembrin and Its Analogs**

Yadessa Melaku^1^, Melat Solomon^2^, Rajalakshmanan Eswaramoorthy^1^,Uwe Beifuss^3^, Vladimir Ondrus^4^, Yalemtsehay Mekonnen^2*^

^1^Chemistry Department, Adama Science and Technology University, 1888, Adama Ethiopia.

^2^Biology Department, Addis Ababa University, Addis Ababa, Ethiopia

^3^ Bioorganische Chemie, Institut für Chemie, Universität Hohenheim, Garbenstraße 30, D-70599 Stuttgart, Germany

^4^Department of Chemical Engineering, FH Münster-University of Applied Sciences, Stegerwaldstrasse 39, D-48565 Steinfurt, Germany

**List of Appendices**

1. ^1^H and ^13^C NMR spectra of **3** in CDCl_3_…………………………………………………...……5

2. ^1^H and ^13^C NMR spectra of **5a** in CDCl_3_…………………………………………………...…..7

3. ^1^H and ^13^C NMR spectra of **5b** in CDCl_3_ …………………………………...………………….9

4. ^1^H and ^13^C NMR spectra of **5c** in CDCl_3_………………………………………………….…..11

5. ^1^H and ^13^C NMR spectra of **5d** in CD_3_COCD_3_………………………………………………..13

6. ^1^H and ^13^C NMR spectra of **5e** in CD_3_CD…………………………………………………….15

7. ^1^H and ^13^C NMR spectra of **5f** in CDCl_3_………………………………………………….…..17

8. ^1^H and ^13^C NMR spectra of **5g** in CDCl_3_………………………………………………….....19

9. ^1^H and ^13^C NMR spectra of **5h** in CDCl_3_…………………………………………………..21

10. ^1^H and ^13^C NMR spectra of **5i** in CDCl_3_………………………………………………….....23

11. ^1^H and ^13^C NMR spectra of **5j** in CDCl_3_…………………………………………………....25

12. ^1^H and ^13^C NMR spectra of **5k** in CDCl_3_…………………………………………………..27

13. ^1^H and ^13^C NMR spectra of **5l** in CDCl_3_…………………………………………………....29

14. ^1^H and ^13^C NMR spectra of **5m** in CDCl_3_…………………………………………………...31

15. ^1^H and ^13^C NMR spectra of **5n** in CDCl_3_…………………………………………………...33

16. ^1^H and ^13^C NMR spectra of **5o** in CDCl_3_…………………………………………………....35

17. ^1^H and ^13^C NMR spectra of **5p** in CDCl_3_………………………………………………..…..37

18. ^1^H and ^13^C NMR spectra of **5q** in CDCl_3_…………………………………………………....39

19. ^1^H and ^13^C NMR spectra of **5r** in CDCl_3_…………………………………………………....41

20. ^1^H and ^13^C NMR spectra of **6a** in CDCl_3_…………………………………………………..43

21. ^1^H and ^13^C NMR spectra of **6b** in CD_3_COCD_3_………………………………………...…..45

22. ^1^H and ^13^C NMR spectra of **6c** in CDCl_3_………………………………………………...…..47

23. ^1^H and ^13^C NMR spectra of **6d** in CDCl_3_………………………………………………...….49

24. ^1^H and ^13^C NMR spectra of **6e** in acetone-d6………………………………………………..51

25. ^1^H and ^13^C NMR spectra of **6f** in CDCl_3_…………………………………..………………...53

26. ^1^H and ^13^C NMR spectra of **6g** in acetone-d6……………………………………………….55

27. ^1^H and ^13^C NMR spectra of **6h** in acetone-d6………………………………………………..57

28. ^1^H and ^13^C NMR spectra of **6l** in CDCl_3_……………………………………………………..59

29. ^1^H and ^13^C NMR spectra of **6m** in CDCl_3_…………………………………………………...61

30. ^1^H and ^13^C NMR spectra of **6n** in CDCl_3_……………………………………………….....63

31. ^1^H and ^13^C NMR spectra of **6o** in CDCl_3_………………………………………………….....65

32. ^1^H and ^13^C NMR spectra of **6p** in CDCl_3_……………………………………………….....67

33. ^1^H and ^13^C NMR spectra of **6q** in CDCl_3_……………………………………………….....69

34. ^1^H and ^13^C NMR spectra of **6r** in CDCl_3_………………………………………………….....71

35. ^1^H and ^13^C NMR spectra of **7a** in CDCl_3_………………………………………………….....73

36. ^1^H NMR spectrum of **7b** in CDCl_3_………………..………………………………………....74

37. ^1^H NMR spectrum of **7d** in CDCl_3_………………………………………………...………...75

38. ^1^H NMR spectrum of **7e** in CDCl_3_…………………………………………………...………76

39. ^1^H and ^13^C NMR spectra of **7f** in CDCl_3_………………………………………..…………...78

40. ^1^H and ^13^C NMR spectra of **7g** in CDCl_3_………………………………………………….....80

41. ^1^H and ^13^C NMR spectra of **7h** in CDCl_3_……………………………………………….....82

42. ^1^H and ^13^C NMR spectra of **7i** in CD_3_OD………………………………………………..…..84

43. ^1^H and ^13^C NMR spectra of **7j** in CD_3_OD…………………………………………………...86

44. ^1^H and ^13^C NMR spectra of **7k** in CD_3_OD………………………………………………...88

45. ^1^H NMR spectra of **7l** in CD_3_OD………………………………………………………...…..89

**1.**^1^H (300 MHz) and ^13^C (100 MHz) NMR spectra of **3** in CDCl_3_

^^

**2.**^1^H (300 MHz) and ^13^C (75 MHz) NMR spectra of **5a** in CDCl_3_

**3.**^1^H (300 MHz) and ^13^C (75 MHz) NMR spectra of **5b** in CDCl_3_

**
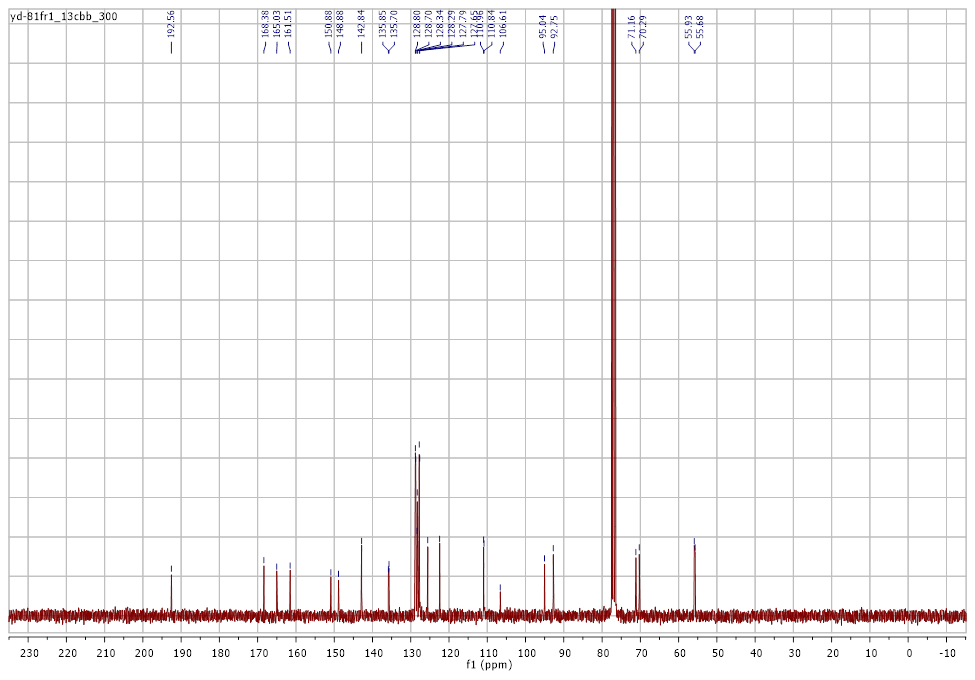
**

**4.**^1^H (300 MHz) and ^13^C (75 MHz) NMR spectra of **5c** in CDCl_3_

**5.**^1^H (300 MHz) and ^13^C (75 MHz) NMR spectra of **5d** in CD_3_COCD_3_

^^

**6.**^1^H (300 MHz) and ^13^C (75 MHz) NMR spectra of **5e** in CD_3_OD

^^

**7.**^1^H (300 MHz) and ^13^C (75 MHz) NMR spectra of **5f** in CDCl_3_

**8.**^1^H (300 MHz) and ^13^C (75 MHz) NMR spectra of **5g** in CDCl_3_

**9.**^1^H (300 MHz) and ^13^C (75 MHz) NMR spectra of **5h** in CDCl_3_

**10.**^1^H (300 MHz) and ^13^C (75 MHz) NMR spectra of **5i** in CDCl_3_

**11.**^1^H (300 MHz) and ^13^C (75 MHz) NMR spectra of **5j** in CDCl_3_

**12.**^1^H (300 MHz) and ^13^C (75 MHz) NMR spectra of **5k** in CDCl_3_

^^

**13.**^1^H (300 MHz) and ^13^C (75 MHz) NMR spectra of **5l** in CDCl_3_

**14.**^1^H (300 MHz) and ^13^C (75 MHz) NMR spectra of **5m** in CDCl_3_

**15.**^1^H (300 MHz) and ^13^C (75 MHz) NMR spectra of **5n** in CDCl_3_

**16.**^1^H (300 MHz) and ^13^C (75 MHz) NMR spectra of **5o** in CDCl_3_

**17.**^1^H (300 MHz) and ^13^C (75 MHz) NMR spectra of **5p** in CDCl_3_

**18.**^1^H (300 MHz) and ^13^C (75 MHz) NMR spectra of **5q** in CDCl_3_

**19.**^1^H (300 MHz) and ^13^C (75 MHz) NMR spectra of **5r** in CDCl_3_

**20.**^1^H (300 MHz) and ^13^C (75 MHz) NMR spectra of **6a** in CDCl_3_

**21.**^1^H (300 MHz) and ^13^C (75 MHz) NMR spectra of **6b** in CD_3_COCD_3_

**22.**^1^H (300 MHz) and ^13^C (75 MHz) NMR spectra of **6c** in CDCl_3_

**23.**^1^H (300 MHz) and ^13^C (75 MHz) NMR spectra of **6d** in CDCl_3_

^^

**24.**^1^H (300 MHz) and ^13^C (75 MHz) NMR spectra of **6e** in CD_3_COCD_3_

**25.**^1^H (300 MHz) and ^13^C (75 MHz) NMR spectra of **6f** in CDCl_3_

**26.**^1^H (300 MHz) and ^13^C (75 MHz) NMR spectra of **6g** in CD_3_COCD_3_

**27.**^1^H (300 MHz) and ^13^C (75 MHz) NMR spectra of **6h** in CD_3_COCD_3_

**28.**^1^H (300 MHz) and ^13^C (75 MHz) NMR spectra of **6i**in CDCl_3_

**29.**^1^H (300 MHz) and ^13^C (75 MHz) NMR spectra of **6m** in CDCl_3_

**30.**^1^H (300 MHz) and ^13^C (75 MHz) NMR spectra of **6n** in CDCl_3_

**31.**^1^H (300 MHz) and ^13^C (75 MHz) NMR spectra of **6o** in CDCl_3_

**32.**^1^H (500 MHz) and ^13^C (125 MHz) NMR spectra of **6p** in CDCl_3_

**33.**^1^H (500 MHz) and ^13^C (125 MHz) NMR spectra of **6q** in CDCl_3_

**34.**^1^H (500 MHz) and ^13^C (125 MHz) NMR spectra of **6r** in CDCl_3_

**35.**^1^H (500 MHz) and ^13^C (125 MHz) NMR spectra of **7a** in CDCl_3_

**36.**^1^H (500 MHz) and ^13^C (125 MHz) NMR spectra of **7b** in CDCl_3_

**37.**^1^H (500 MHz) and ^13^C (125 MHz) NMR spectra of **7d** in CDCl_3_

**38.**^1^H (500 MHz) and ^13^C (125 MHz) NMR spectra of **7e** in CDCl_3_

**39.**^1^H (500 MHz) and ^13^C (125 MHz) NMR spectra of **7f** in CDCl_3_

**40.**^1^H (500 MHz) and ^13^C (125 MHz) NMR spectra of **7g** in CDCl_3_

**41.**^1^H (500 MHz) and ^13^C (125 MHz) NMR spectra of **7h** in CDCl_3_

**42.**^1^H (500 MHz) and ^13^C (125 MHz) NMR spectra of **7i** in CD_3_OD

**43.**^1^H (500 MHz) and ^13^C (125 MHz) NMR spectra of **7j** in CD_3_OD

**44.**^1^H (500 MHz) and ^13^C (125 MHz) NMR spectra of **7k** in CD_3_OD

**45.**^1^H NMR spectra of **7l** in CD_3_OD
