# Supplementary material for: Synthesis, antiplasmodial activity and in silico molecular docking study of pinocembrin and its analogs
Source: BMC Chem. 2022 May 24;16(1):36. doi: 10.1186/s13065-022-00831-z (PMC9128099; doi:10.1186/s13065-022-00831-z)

Supplementary Material 2: Molecular docking analysis of the synthesized compounds

The 2D and 3D binding interactions of compound **7a-j** against *Plasmodium falciparum* dihydrofolate reductase-thymidylate synthase (PfDHFR-TS) (PDB ID 1J3I) are presented in Figure 1-11. The ribbon model shows the binding pocket structure of PfDHFR-TS with the compound**s**. Hydrogen bond between compounds and amino acids are shown as green dash lines, hydrophobic interaction are shown as pink lines.

Figure 1: Molecular docking of compound **7a** against *Plasmodium falciparum* dihydrofolate reductase-thymidylate synthase

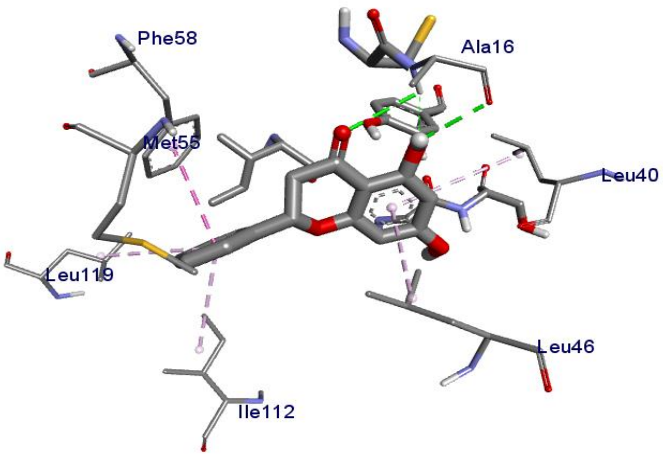

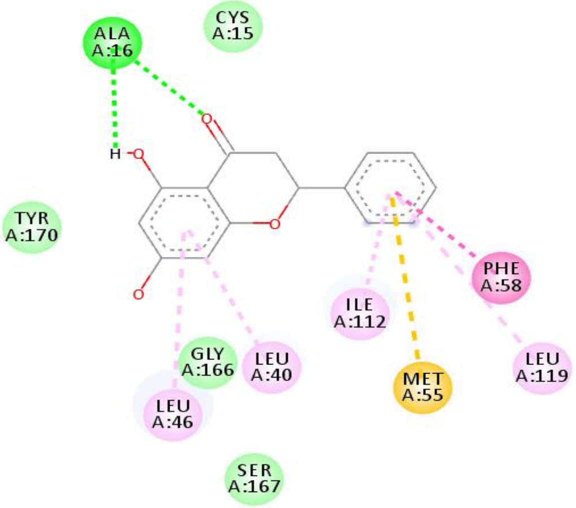

Figure 2: Molecular docking of compound **7b** against *Plasmodium falciparum* dihydrofolate reductase-thymidylate synthase

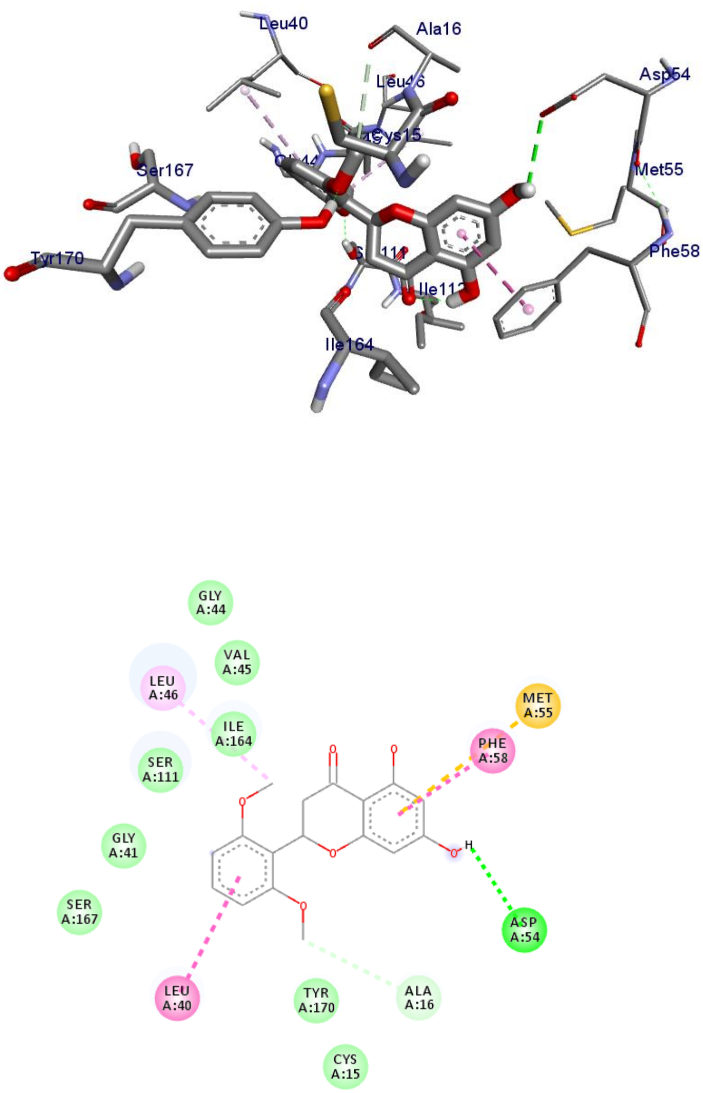

Figure 3: Molecular docking of compound **7c** against *Plasmodium falciparum* dihydrofolate reductase-thymidylate synthase

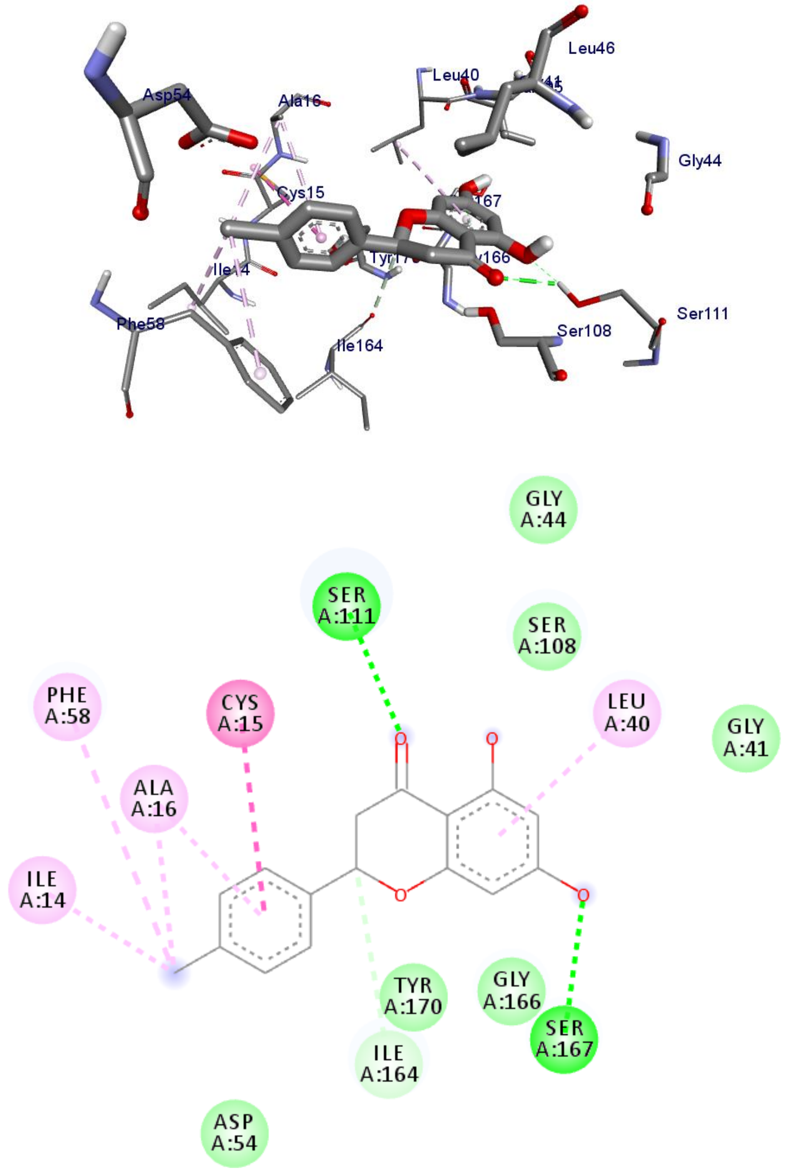

Figure 4: Molecular docking of compound **7d** against *Plasmodium falciparum* dihydrofolate reductase-thymidylate synthase

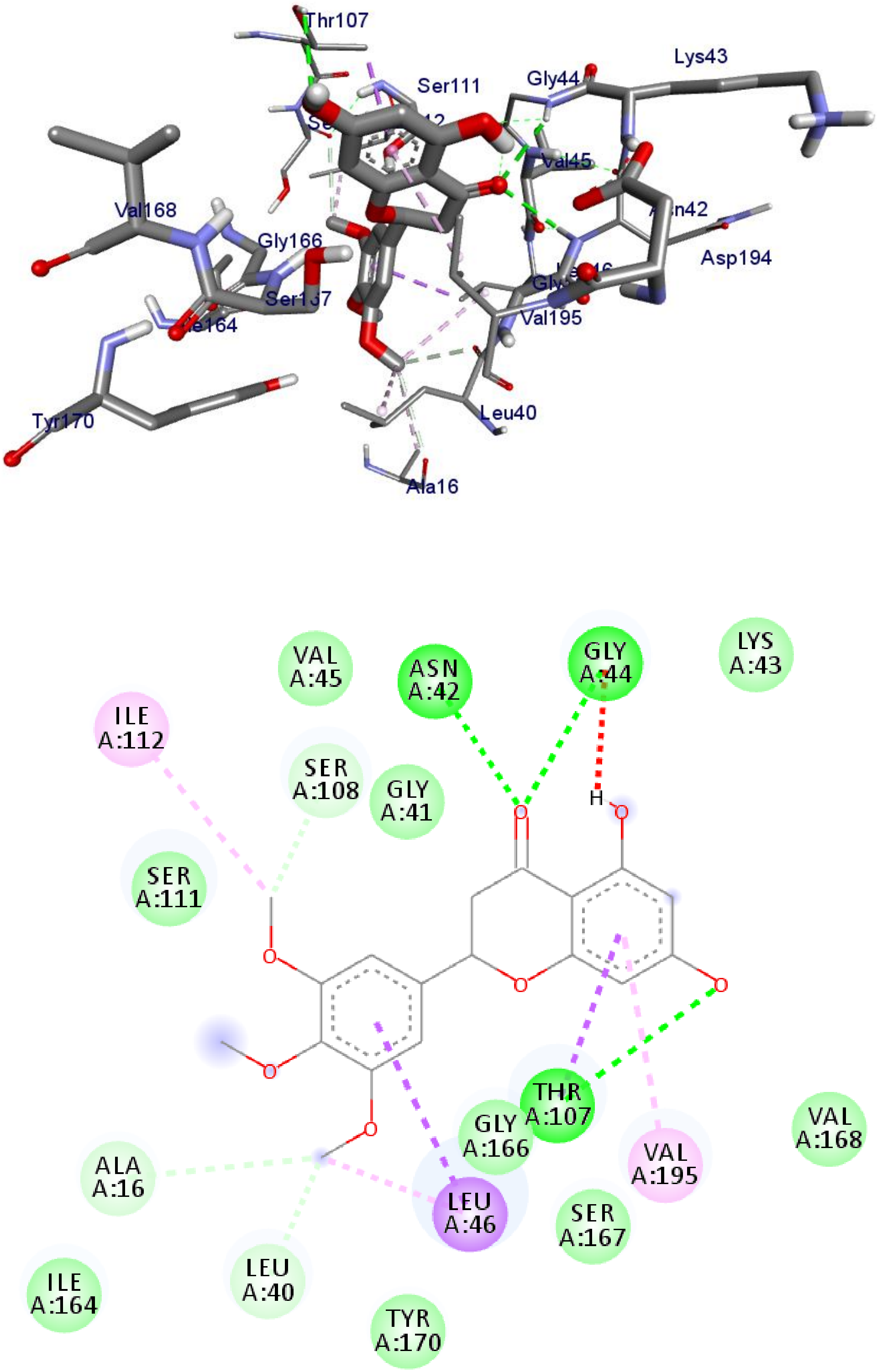

Figure 5: Molecular docking of compound **7e** against *Plasmodium falciparum* dihydrofolate reductase-thymidylate synthase

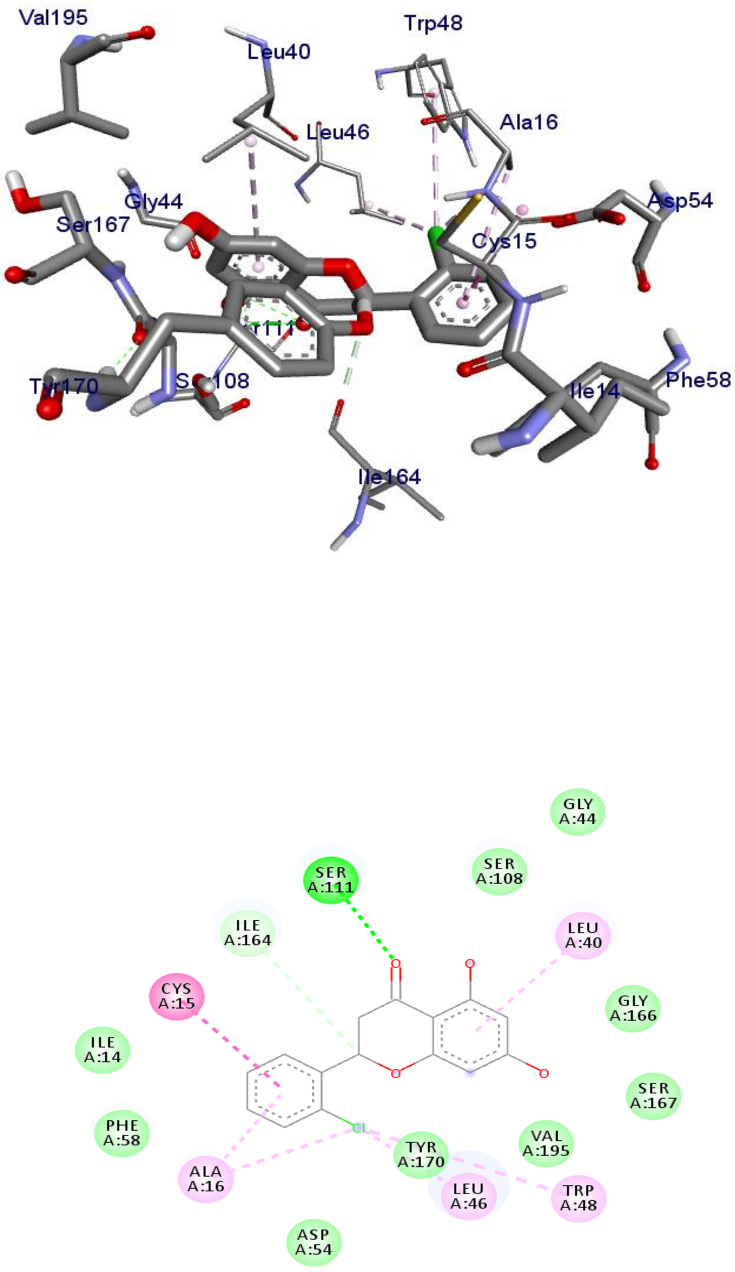

Figure 6: 2D and 3D binding interactions of 7f against *Plasmodium falciparum* dihydrofolate reductase-thymidylate synthase

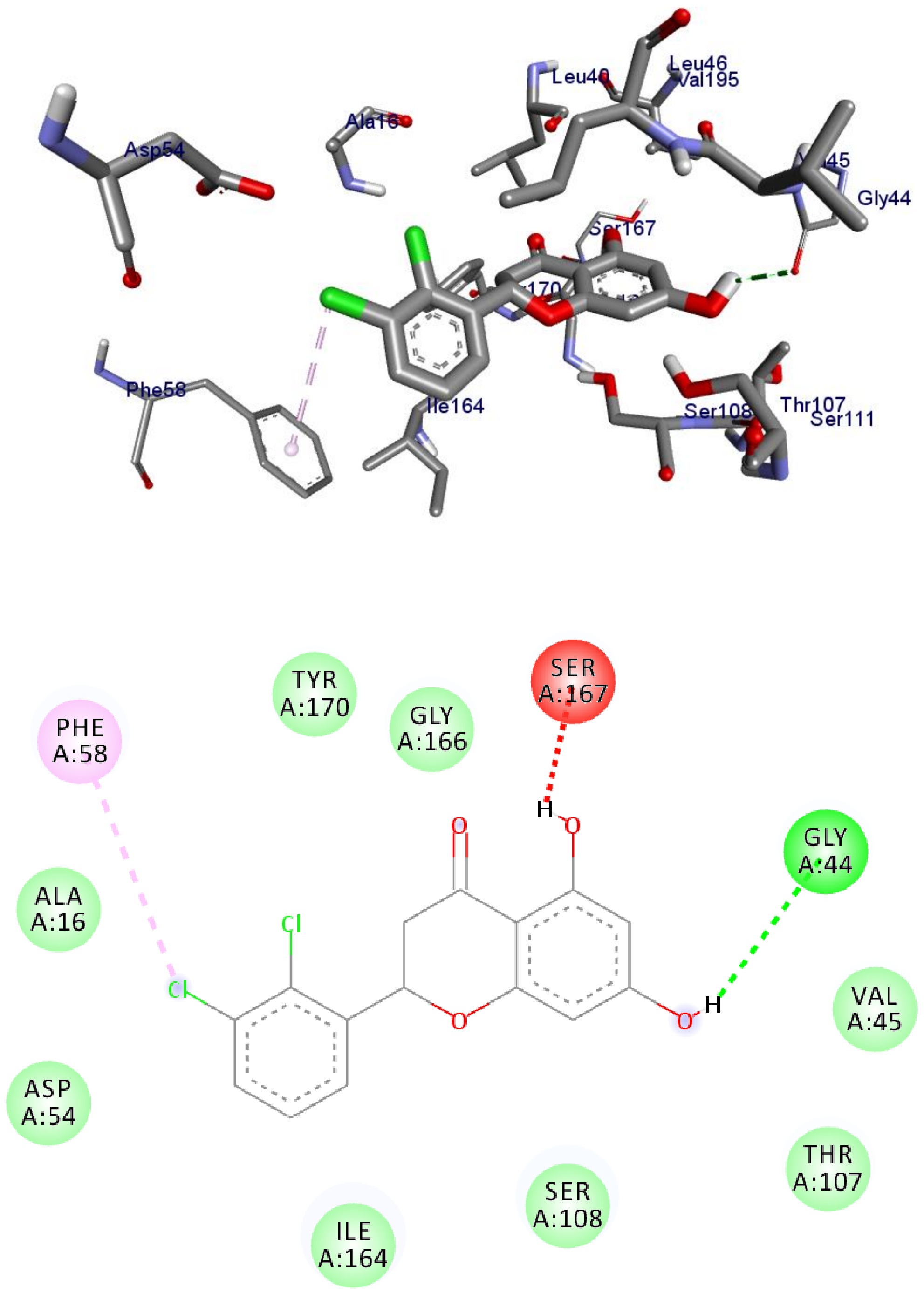

Figure 7: Molecular docking of compound **7g** against *Plasmodium falciparum* dihydrofolate reductase-thymidylate synthase

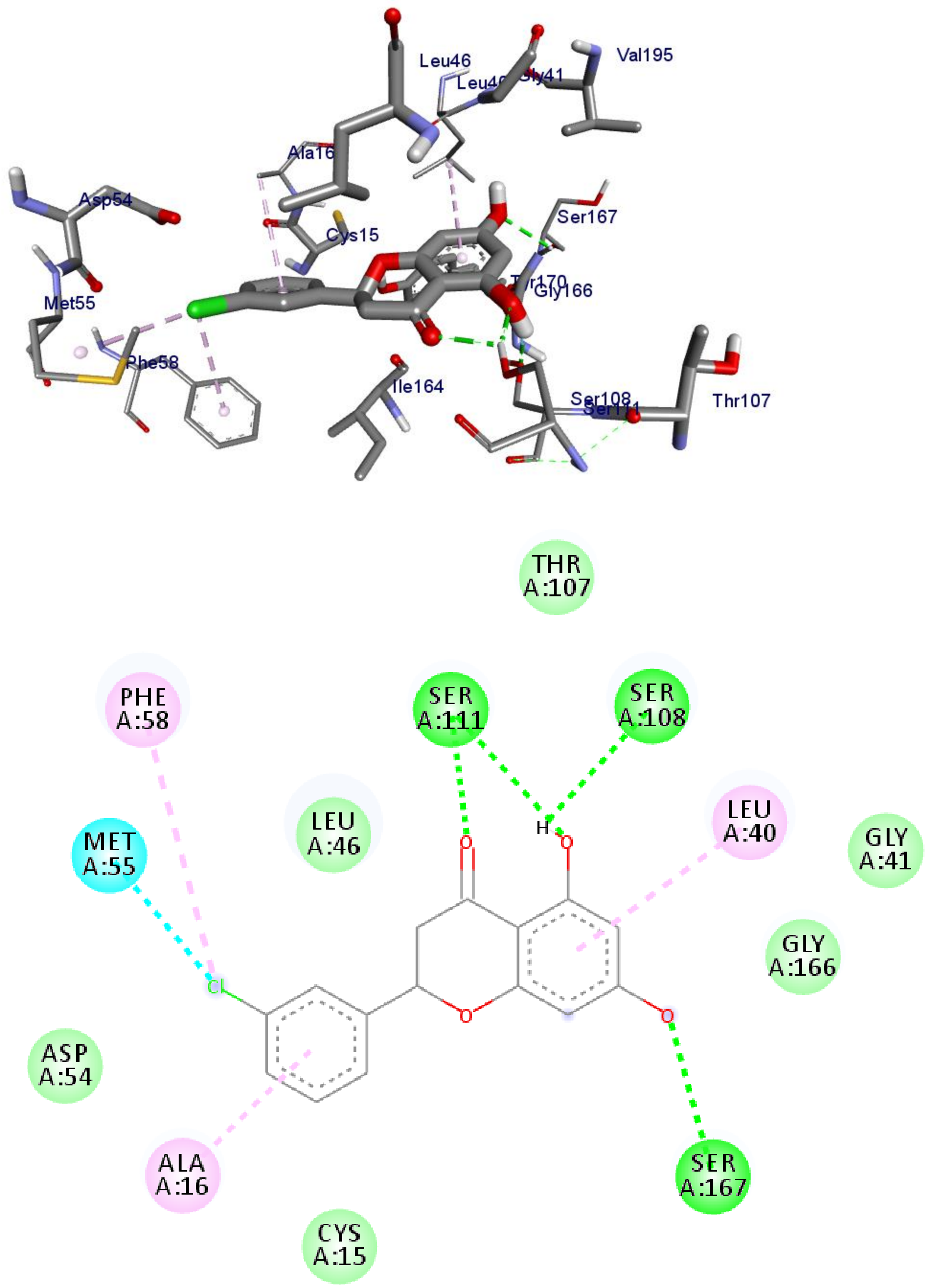

Figure 8: Molecular docking of compound **7h** against *Plasmodium falciparum* dihydrofolate reductase-thymidylate synthase

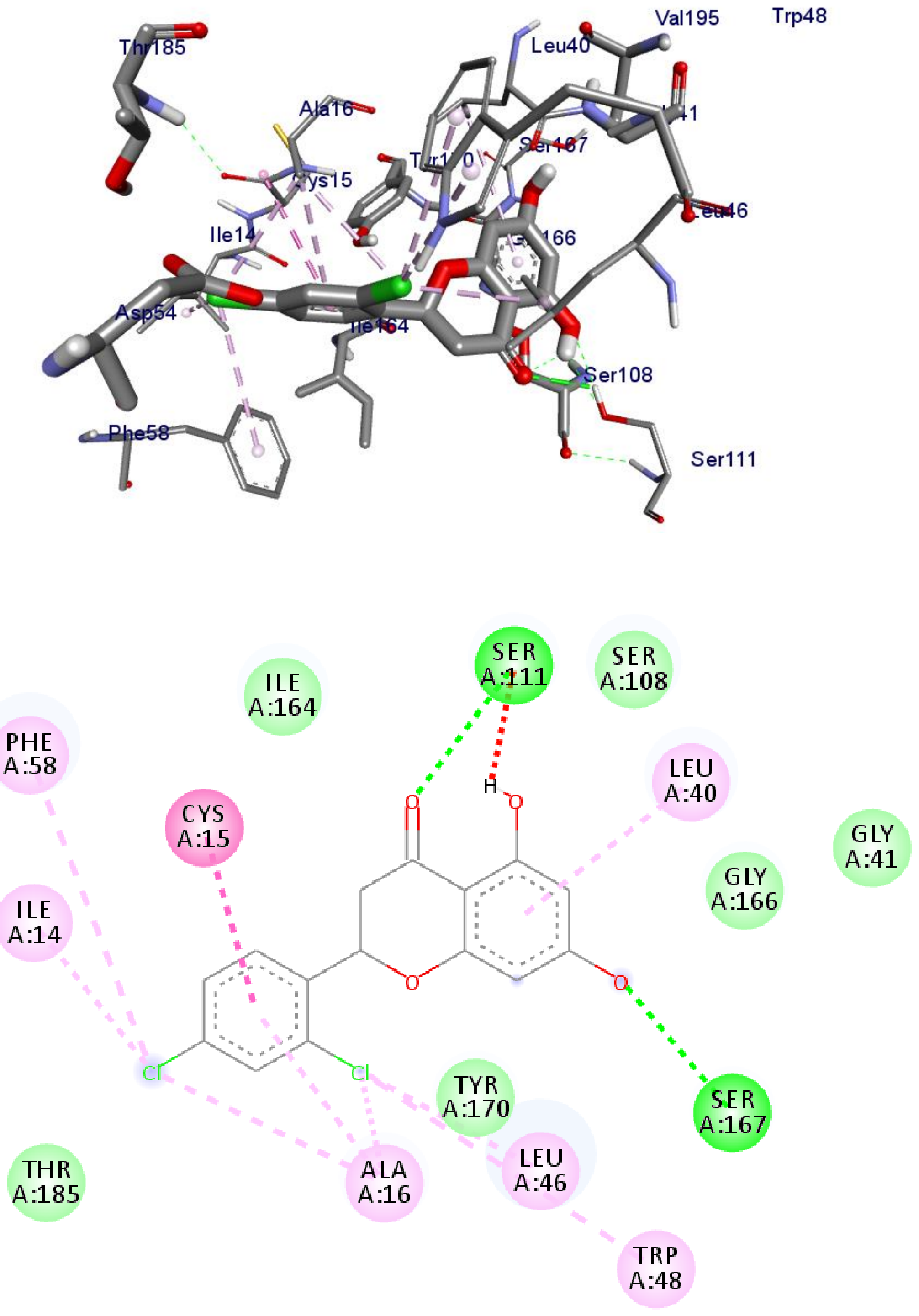

Figure 9: Molecular docking of compound **7j** against *Plasmodium falciparum* dihydrofolate reductase-thymidylate synthase

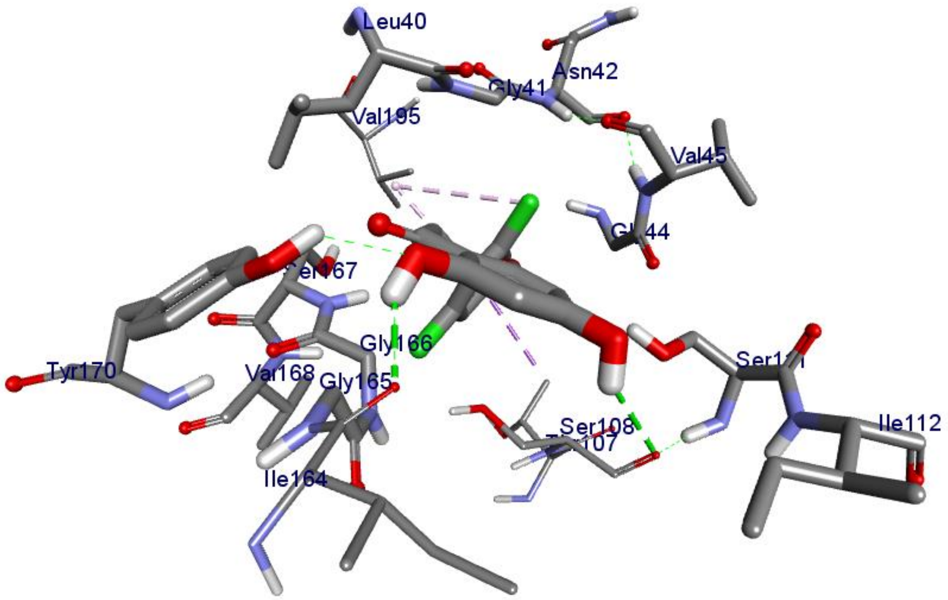
Leu40
Asn42
Gly41
Val195
Val45
Gly166
Tyr170
Val168
Gly165
Ser108
Ile112
Ile164

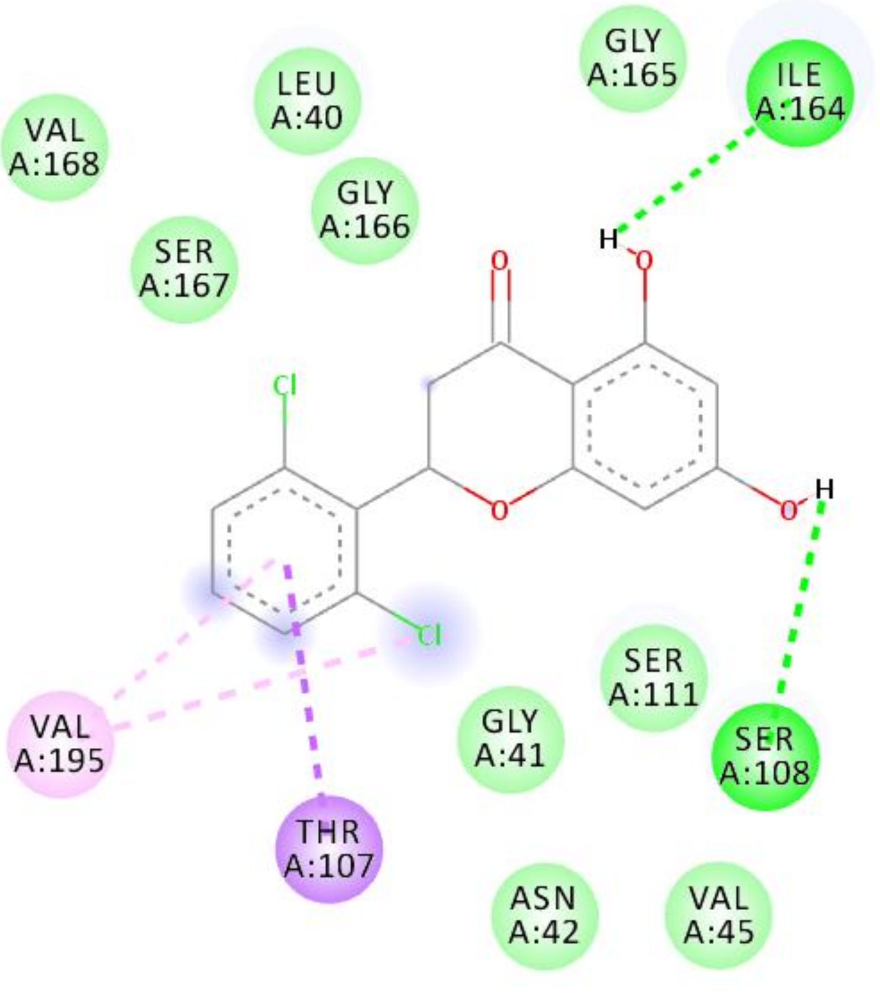
VAL A:168
LEU A:40
GLY A:165
ILE A:164
GLY A:166
SER A:167
H
O
O
Cl
O
O
H
Cl
SER A:111
VAL A:195
GLY A:41
SER A:108
THR A:107
ASN A:42
VAL A:45

Figure 10: Molecular docking of compound **7k** against *Plasmodium falciparum* dihydrofolate reductase-thymidylate synthase

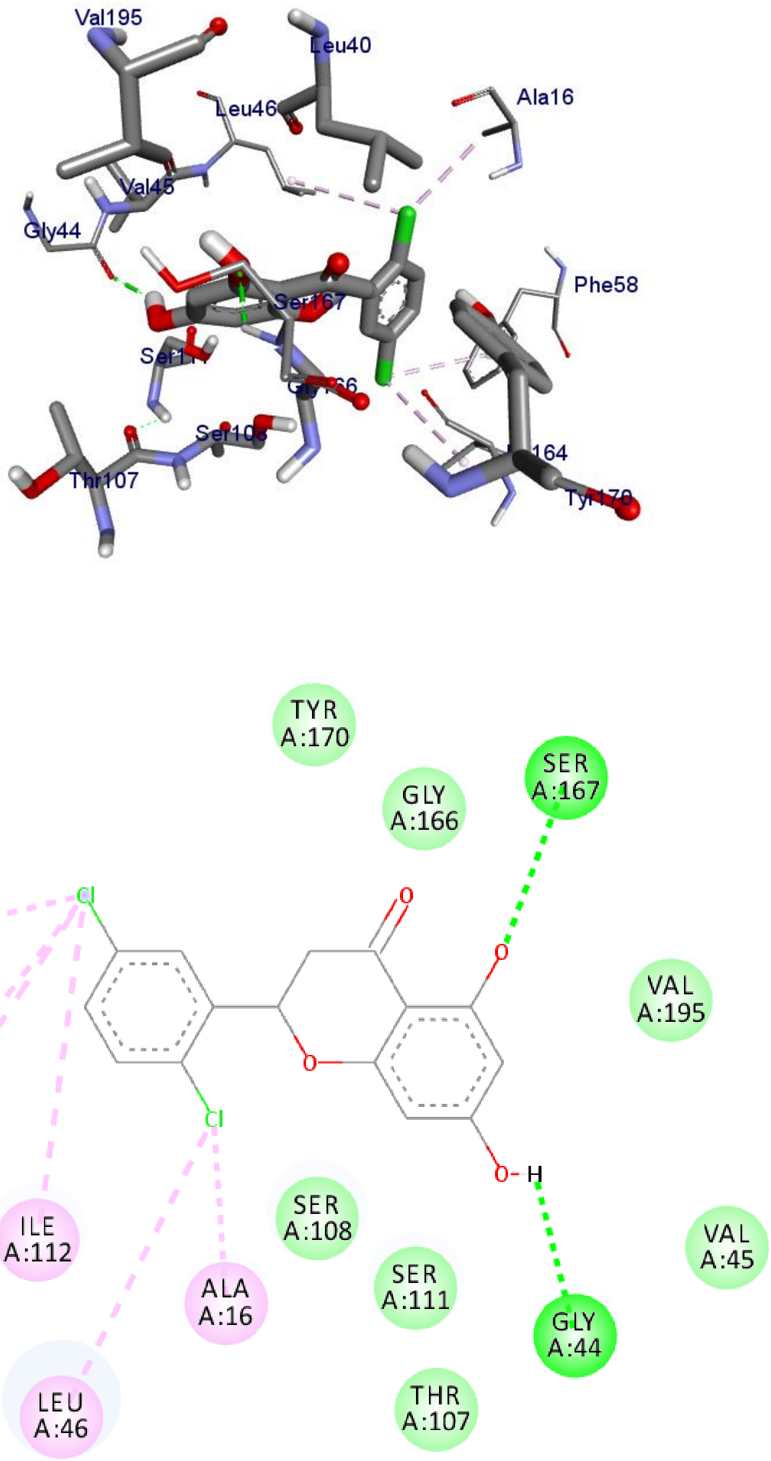

Figure 11: Molecular docking of compound **7l** against *Plasmodium falciparum* dihydrofolate reductase-thymidylate synthase

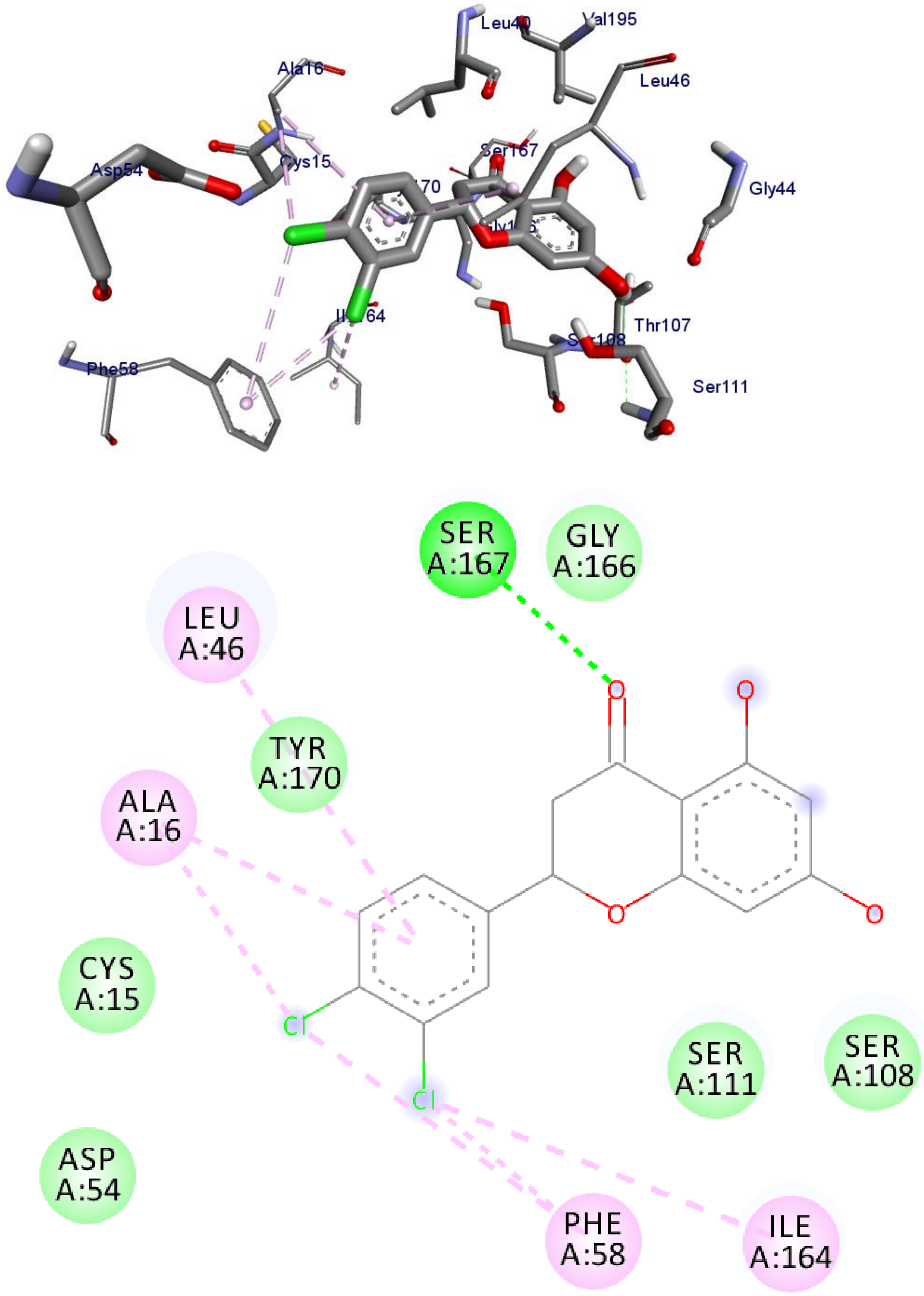

Figure 12: Molecular docking of compound chloroquine against *Plasmodium falciparum* dihydrofolate reductase-thymidylate synthase

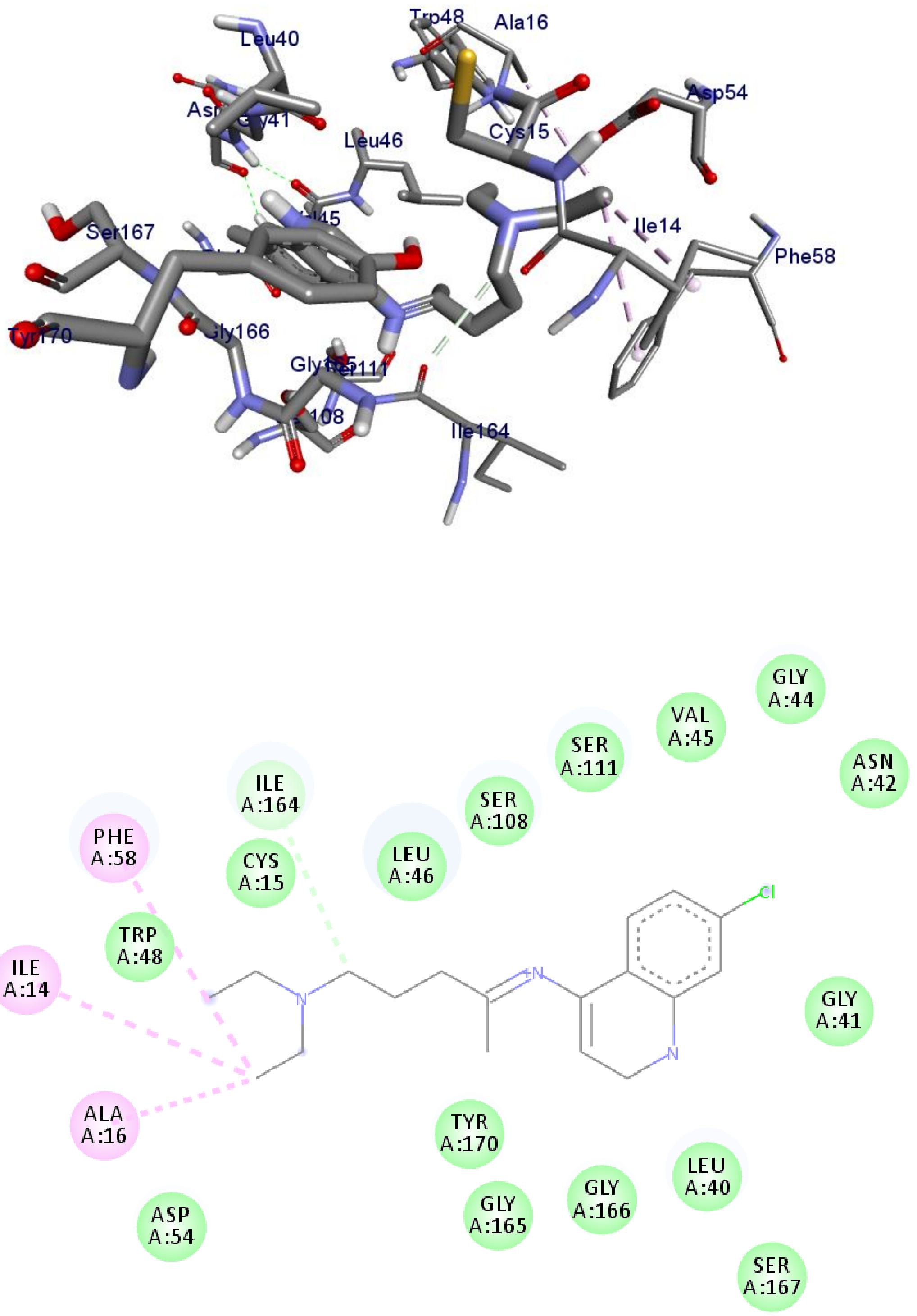

Supplement: Supplementary file 2 — Additional file 2. The NMR spectra and molecular docking simulations for the synthetic compounds are included within Additional materials (Additional file 1 and 2). [file 13065_2022_831_MOESM2_ESM.pdf]
